# High-quality haplotype-resolved genome assembly of cultivated octoploid strawberry

**DOI:** 10.1093/hr/uhad002

**Published:** 2023-01-04

**Authors:** Jianxin Mao, Yan Wang, Baotian Wang, Jiqi Li, Chao Zhang, Wenshuo Zhang, Xue Li, Jie Li, Junxiang Zhang, He Li, Zhihong Zhang

**Affiliations:** Liaoning Key Laboratory of Strawberry Breeding and Cultivation, College of Horticulture, Shenyang Agricultural University, 120 Dongling Road, Shenyang 110866, China; Liaoning Key Laboratory of Strawberry Breeding and Cultivation, College of Horticulture, Shenyang Agricultural University, 120 Dongling Road, Shenyang 110866, China; Laboratory of Protected Horticulture (Shenyang Agricultural University), Ministry of Education, Shenyang 110866, China; Liaoning Key Laboratory of Strawberry Breeding and Cultivation, College of Horticulture, Shenyang Agricultural University, 120 Dongling Road, Shenyang 110866, China; Liaoning Key Laboratory of Strawberry Breeding and Cultivation, College of Horticulture, Shenyang Agricultural University, 120 Dongling Road, Shenyang 110866, China; Liaoning Key Laboratory of Strawberry Breeding and Cultivation, College of Horticulture, Shenyang Agricultural University, 120 Dongling Road, Shenyang 110866, China; School of Information Science and Technology, ShanghaiTech University, 393 Middle Huaxia Road, Shanghai 201210, China; Liaoning Key Laboratory of Strawberry Breeding and Cultivation, College of Horticulture, Shenyang Agricultural University, 120 Dongling Road, Shenyang 110866, China; Liaoning Key Laboratory of Strawberry Breeding and Cultivation, College of Horticulture, Shenyang Agricultural University, 120 Dongling Road, Shenyang 110866, China; Liaoning Key Laboratory of Strawberry Breeding and Cultivation, College of Horticulture, Shenyang Agricultural University, 120 Dongling Road, Shenyang 110866, China; Laboratory of Protected Horticulture (Shenyang Agricultural University), Ministry of Education, Shenyang 110866, China; Liaoning Key Laboratory of Strawberry Breeding and Cultivation, College of Horticulture, Shenyang Agricultural University, 120 Dongling Road, Shenyang 110866, China; Laboratory of Protected Horticulture (Shenyang Agricultural University), Ministry of Education, Shenyang 110866, China; Liaoning Key Laboratory of Strawberry Breeding and Cultivation, College of Horticulture, Shenyang Agricultural University, 120 Dongling Road, Shenyang 110866, China; Laboratory of Protected Horticulture (Shenyang Agricultural University), Ministry of Education, Shenyang 110866, China

## Abstract

Cultivated strawberry (*Fragaria* × *ananassa*), a perennial herb belonging to the family Rosaceae, is a complex octoploid with high heterozygosity at most loci. However, there is no research on the haplotype of the octoploid strawberry genome. Here we aimed to obtain a high-quality genome of the cultivated strawberry cultivar, “Yanli”, using single molecule real-time sequencing and high-throughput chromosome conformation capture technology. The “Yanli” genome was 823 Mb in size, with a long terminal repeat assembly index of 14.99. The genome was phased into two haplotypes, Hap1 (825 Mb with contig N50 of 26.70 Mb) and Hap2 (808 Mb with contig N50 of 27.51 Mb). Using the combination of Hap1 and Hap2, we obtained for the first time a haplotype-resolved genome with 56 chromosomes for the cultivated octoploid strawberry. We identified a ~ 10 Mb inversion and translocation on chromosome 2-1. 104 957 and 102 356 protein-coding genes were annotated in Hap1 and Hap2, respectively. Analysis of the genes related to the anthocyanin biosynthesis pathway revealed the structural diversity and complexity in the expression of the alleles in the octoploid *F. × ananassa* genome. In summary, we obtained a high-quality haplotype-resolved genome assembly of *F. × ananassa*, which will provide the foundation for investigating gene function and evolution of the genome of cultivated octoploid strawberry.

## Introduction

The cultivation area of strawberry, which belongs to the family Rosaceae and genus *Fragaria*, is increasing worldwide [1]. So far, 24 species of *Fragaria* with different levels of ploidy, ranging from diploids to decaploid, have been identified [2]. The woodland strawberry (*Fragaria vesca*) is a diploid, which is one of the four subgenomes of the octoploid species [3]. Due to its relatively simple genome structure (~240 Mb, 2*n* = 2*x* = 14), self-compatibility, short life history, and easy genetic transformation, woodland strawberry is widely used for studying the gene function of horticultural crops. Shulaev *et al*. sequenced the woodland strawberry accession “Hawaii 4” and assembled the first strawberry reference genome using second-generation technology [4]. However, because of technical limitations, the quality of this genome is only that of the draft level. To complete the genome, researchers have updated the woodland strawberry genome version 1.1a2 annotation using a new genetic model prediction algorithm and the V2.0 assembled woodland strawberry genome with an interspecific high-density genetic map. Compared with the annotation obtained using GeneMark, the new Maker annotation acquired many more predicted protein-coding genes, and the number of coding regions was also increased as well as the total coding length [5, 6]. Using single molecule real-time (SMRT) sequencing technology, Patrick *et al*. sequenced and assembled a near-complete genome of woodland strawberry with contig N50 reach to ~7.9 million base pairs (Mb), which represented a ~ 300-fold improvement over the previous study [7]. Thus, the woodland strawberry genome has now been updated to version 4.0. In addition, the high-quality genomes of several other diploid strawberry species, such as *F. nilgerrensis*, *F. viridis* and *F. pentaphylla*, were also assembled using SMRT sequencing technology [8–10]. In addition to “Hawaii4”, the high-quality genomes of two other woodland strawberry accessions, “CFRA2339” and “Yellow Wonder”, were assembled [11, 12].

Cultivated strawberry (*F.* × *ananassa*) is planted worldwide. Because of the complex octoploid genome (2*n* = 8*x* = 56), with as many as four diploid ancestors [2], the genetic analysis of cultivated strawberry is extremely difficult. To better analyse the octoploid genetic background of *F.* × *ananassa*, Hirakawa *et al*. sequenced and assembled the first draft genome of the cultivated strawberry and those of three other wild diploid strawberry species, including *F. nipponica*, *F. iinumae* and *F. bucharica*, and a wild tetraploid strawberry *F. orientalis*. With these data, the study preliminarily identified two diploid contributors, *F. vesca* and *F. iinumae*, in the cultivated octoploid genome [13]. Edger *et al.* sequenced the cultivated octoploid strawberry cultivar “Camarosa”, assembled the chromosome-level genome of *F.* × *ananassa* for the first time with scaffold N50 equal to 5.98 Mb, and revealed that the four contributors, *F. vesca, F. iinumae*, *F. nipponica* and *F. viridis*, harboured subgenomes of *F.* × *ananassa*. At the same time, the study also found that the *F. vesca* genome plays a dominant role in the four subgenomes and in controlling the metabolism and disease resistance traits of cultivated octoploid strawberry [14]. And then, Hardigan *et al*. unravelled the complex ancestry and domestication history of cultivated strawberry and found that nucleotide diversity and heterozygosity were significantly lower in cultivated strawberries [15]. But the hypothesis on hybrid ancestry of octoploid cultivated strawberry was controversial [16, 17]. Cauret *et al*. assembled the octoploid *F. chiloensis* genome and challenged subgenome assignments of *F.* × *ananassa* [18]. Lee *et al*. assembled a chromosome-level genome of *F.* × *ananassa* for a homozygous inbred line, “Wongyo 3115”, using long- and short-read sequencing strategies. The quality of the “Wongyo 3115” genome was considerably better than that of “Camarosa”, as scaffold N50 reached 27.37 Mb. In addition, five quantitative trait loci located on chr3-3, chr5-1, chr6-1 and chr6-4, and the candidate genes of firmness were identified, which revealed the molecular and genetic mechanisms contributing to the firmness of strawberry [19].

Chromosomal structural variations, which occur frequently during polyploidisation and whole-genome duplication events, are the key behind the phenotypic differences between ancestors and descendants [20, 21]. Chromosomal structural variations including deletion, duplication, inversion, and translocation, may lead to trait transition and even genetic diseases such as the stick-eye and notched wing phenomenon in *Drosophila* and Down syndrome in humans [22–24]. In plant, Endo and Tsunewaki were the first to identify the molecular mechanism underlying variations in chromosome structure, which involved “gametocidal chromosomes” [25], a class of chromosomes with preferential transmission effects. Gametocidal chromosomes induce breakage and reconnection with other chromosomes in gametes that do not contain gametocidal chromosomes, resulting in deletion, translocation, and other chromosomal structural variations [26, 27]. So far, chromosomal structural variation has been identified in animals and plants, but not in strawberry. In addition, high-quality chromosome level genome assembly is also helpful for the study of haplotype of octoploid strawberry.

In this study, using a new cultivated strawberry cultivar, “Yanli”, as the material, and SMRT and high-throughput chromosome conformation capture (Hi-C) technology, we assembled a high-quality haplotype-resolved genome of *F. × ananassa* with 56 chromosomes, with contig N50 being 26.93 Mb. We found a ~ 10 Mb inversion and translocation on chromosome 2–1. Analysis of genes related to the anthocyanin biosynthesis pathway revealed structural diversity and complexity in the expression of alleles in the octoploid *F. × ananassa* genome.

## Results

### Sequencing and assembly of the “Yanli” (*F. × ananassa)* genome

Our goal was to obtain high-quality genomic information on “Yanli”, a high-quality cultivar of *F.* × *ananassa* obtained by crossing the Japanese strawberry “Tochiotome” as the female parent with a strawberry accession “08-A-01”, introduced from America, as the male parent. To obtain basic information regarding this genome, we performed a genome survey using Illumina-HiSeq. In total, ~115.58 Gb Illumina short reads were obtained from two sequencing lines (Supplementary Data Table S1). The results of k-mer analysis showed that the sample genome size was 850 Mb, heterozygosity rate was 1.04%, and repeat sequence ratio was 73.98% (Supplementary Data Table S2, Supplementary Data Fig. S1). After obtaining the draft genome sequence, we used SMRT sequencing technology to obtain genomic information of higher quality. SMRT yielded longer and more accurate reads than Illumina using the circular consensus sequencing (CCS) method. After checking the quality of gDNA using Qubit 3.0 and Nanodrop 2000, the SMRT bell library was constructed and sequenced on the Pacific Bioscience Sequel II platform. High quality HiFi reads were obtained after filtering low quality sequences and the PacBio HiFi data were assembled using HiFiasm. A 823 Mb of genome was assembled with 390 contigs and N50 reaching 26.93 Mb (Supplementary Data Table S3). Statistical analysis of the nucleotide content showed 39.63% GC contained no “N”, which represented high quality of single nucleotide sequence (Supplementary Data Fig. S2). By comparing the second and third generation data with the genome, the mapping rates of second and third generation data to the genome were 99.61% and 99.98%, while the coverage rates were 99.94% and 99.86%, respectively (Supplementary Data Table S4). Furthermore, Benchmarking Universal Single-Copy Orthology (BUSCO) analysis showed that the mapping rate with embryophyta_odb9 gene set was 93.26% (Supplementary Data Table S5). Long terminal repeat (LTR) analysis displayed that LTR assembly index (LAI) was 14.99, which represents a reference level of genome integrity (Supplementary Data Table S6). Homozygous single nucleotide polymorphism (SNP) and insertion/deletion (InDel) rate were zero, which is indicative of high genome accuracy (Supplementary Data Table S7).

We compared the “Yanli” genome with that of “Camarosa”, the first reported chromosomal-scale octoploid strawberry genome [11], “Wongyo 3115” [12], and “Royal Royce^”^ [28]. The length of contig N50 is an important parameter for evaluating genome quality, and the “Yanli” genome contig N50 length (26.93 Mb) was 2.7 times that of “Wongyo 3115” (9.85 Mb), 2.4 times that of “Royal Royce” (11.04 Mb) and 34 times that of “Camarosa” (0.79 Mb). Smaller L50 (the number of contigs, the sum of whose length produces N50) represents higher genome quality; the contig L50 of the “Yanli” genome (count of 14) was half of that of “Wongyo 3115” (count of 29) and “Royal Royce” (count of 23), and 3/1000th that of “Camarosa” (count of 4492). After assembly to the scaffold level, the scaffold N50 of the “Wongyo 3115” and “Royal Royce” genomes were almost of the same quality as the contig N50 of “Yanli”, whlie the scaffold N50 of the “Camarosa” genome only reached 5.98 Mb (Table 1).

**Table 1:** Genome assembly of “Camarosa”, “Wongyo3115”, “Royal Royce”, and “Yanli”

Genomic features	Camarosa (*Nat. Genet.*, 2019)	Wongyo 3115 (*Front. Plant Sci.*, 2021)	Royal Royce (*BioRxiv*, 2021)	Yanli
Total data (Gb)	94.85	93.62	83	298.86
**Contig**				
Number of contigs	48 802	323	136	390
Total size of contigs	1206.26	805.66	784.72	823.88
N50 contig length	0.79	9.85	11.04	26.93
L50 contig count	4492	29	23	14
**Scaffold**				
Number of scaffolds	25 426	208	65	-
Total size of scaffolds	1213.65	805.68	786.54	-
N50 scaffold length	5.98	27.37	25.98	-
L50 scaffold count	64	13	14	-

**Figure 1:**
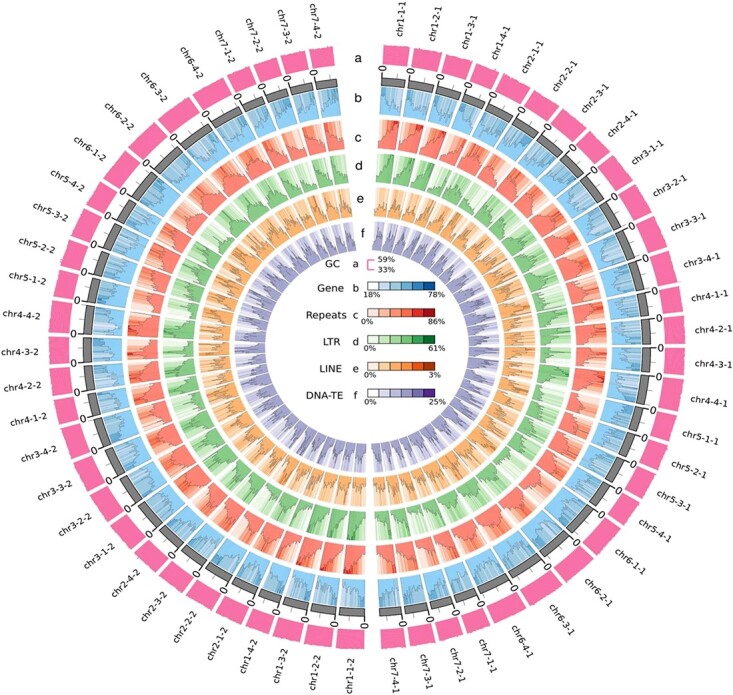
**Overview of the haplotype-resolved genome assembly of the “Yanli” (*Fragaria × ananassa)* genome.** The tracks (moving inwards) indicate the distribution of diverse genome features. **(a)** Guanine-cytosine (GC) content. **(b)** Gene density. **(c)** Repeat density. **(d)** Long terminal repeat (LTR) density. **(e)** Long interspersed nuclear element (LINE) density. **(f)** DNA-TE density. All statistics were computed for windows of 100 kb.

Next, we generated 123.28 GB of Illumina data to build a Hi-C library and then achieved global phasing by HiFiasm combined with precise local haplotype information in HiFi data and long-range interaction information in Hi-C data to phase the genome into two haplotypes named Hap1 and Hap2. Two sets of assembly results containing 56 chromosomes were constructed. The genome size of Hap1 was 824.84 Mb, with 628 contigs (N50 of 26.70 Mb), 647 scaffolds (N50 of 27.31 Mb), and 95.01% anchoring rate. The genome size of Hap2 was 808.07 Mb, with 278 contigs (N50 of 27.51 Mb), 316 scaffolds (N50 of 27.51 Mb), and 96.29% anchoring rate (Table 2, Supplementary Data Table S8). The Hi-C heat maps were used to validate interactions and the result showed that low-level interactions occurred between rather than within pseudochromosomes (Supplementary Data Fig. S3). The quality of the assembly was evaluated by BUSCO [29]. Hap1 showed 98.2% coverage of the embryophyta_odb 10 gene set, whereas Hap2 showed 98.0% coverage (Supplementary Data Table S9). Finally, we combined the haplotypes of Hap1 and Hap2 to obtain the first cultivated octoploid strawberry genome, with all 56 chromosomes assembled and visualized as a genome circle map (Fig. 1, Supplementary Data Table S10).

**Table 2:** Statistics of two haplotype assemblies

Assembly level	Name	Hap1	Hap2
Contig assembly	Contigs number	628	278
	Assembly size (bp)	824 838 780	808 073 877
	N50	26.70	27.51
Scaffold assembly	Scaffold number	647	316
	Assembly size (bp)	824 841 180	808 074 877
	N50	27.31	27.51
Chromosomes Number		28	28
Unanchored Number		619	288
Total Length (Mb)		825	808
Chromosome anchoring rate (%)		95.01	96.29

After comparing with the RepBase database (http://www.girinst.org/repbase), 45.05% and 43.75% repetitive sequences were identified in Hap1 and Hap2, respectively (Supplementary Data Table S11). Among transposable elements (TEs), the average content of class II elements (DNA transposons), long interspersed nuclear elements (LINEs), short interspersed nuclear elements (SINEs), and LTRs in Hap1 and Hap2 were 12.72%, 1.62%, 0.12%, and 24.55%, respectively (Supplementary Data Table S12). RepeatMasker and *de novo* prediction was performed to annotate the divergence of the four main TE types (Supplementary Data Fig. S4, Supplementary Data Fig. S5). Repeat density and LTR density between homologous chromosomes showed the same trend except that on chromosome 2, and this phenomenon was observed both in Hap1 and Hap2.

### Chromosomal structural variant on chromosome 2

**Figure 2:**
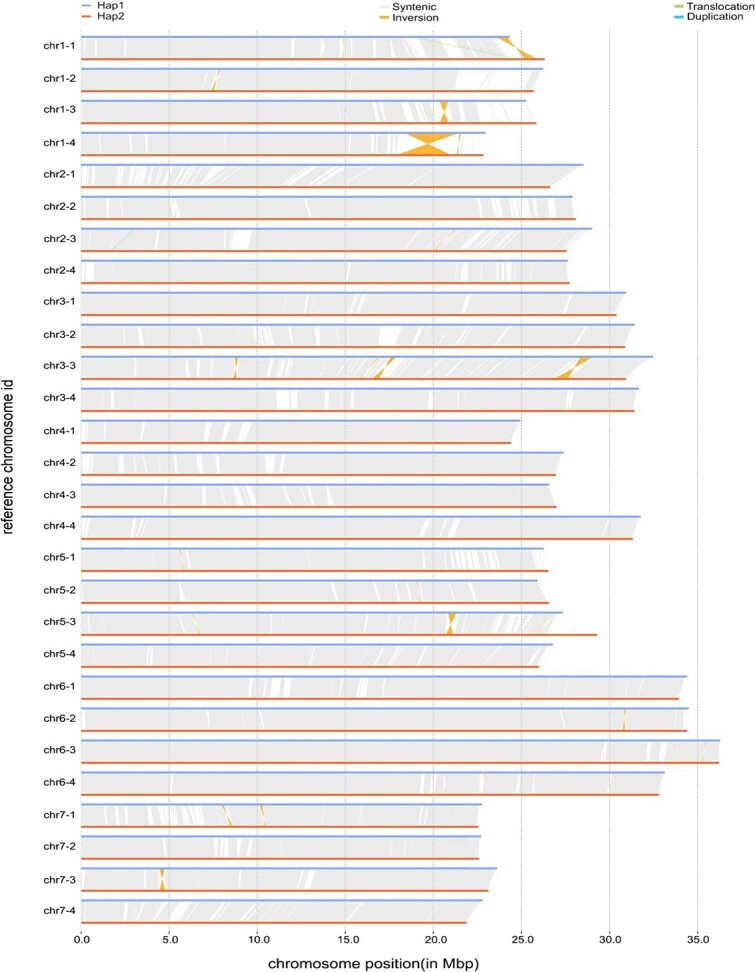
**Schematic diagram of collinearity analysis.** Collinearity analysis between Hap1 and Hap2.

As studies on chromosomal structure require high-quality chromosomally assembled genomes, we validated the quality of the assembled chromosomes. Collinearity analysis between Hap1 and Hap2 by SyRI version 1.6.3 [30] revealed high collinearity between each pair of homologous chromosomes (Fig. 2). We next compared Hap1 and Hap2 with the reported “Wongyo 3115” and “Hawaii 4” genomes, respectively. JCVI version 1.1.22 [31] was used for collinearity analysis and visualization. Both Hap1 and Hap2 showed high collinearity with the “Wongyo 3115” genome and relatively high homology with the “Hawaii 4” genome (Supplementary Data Fig. S6, Supplementary Data Fig. S7). The above results were indicative of successful haplotype phasing and a high-quality chromosomally assembled genome.

**Figure 3:**
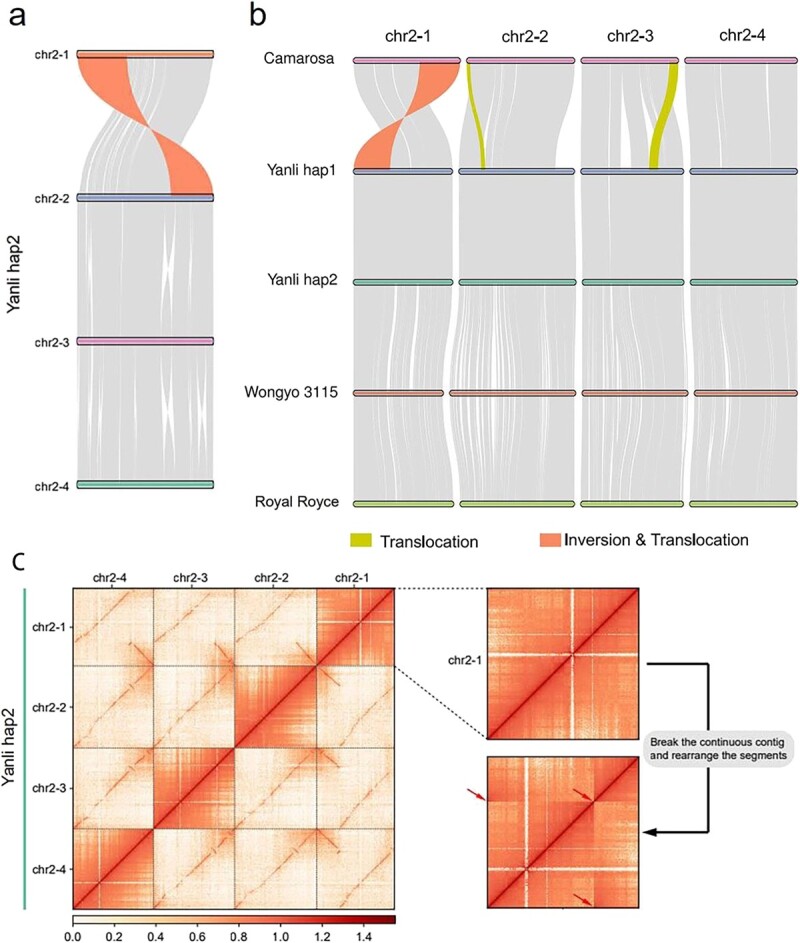
**Chromosome structural variation on chromosome 2–1. (a)** Collinearity analysis between chromosome 2–1 and chromosome 2–2, 2–3, and 2–4. **(b)** Collinearity analysis among chromosomes 2 of “Camarosa”, “Wongyo 3115”, “Royal Royce” and “Yanli” Hap1 and Hap2. **(c)** Hi-C heat map analysis on chromosome 2–1, 2–2, 2–3, and 2–4. Yellow block represents translocation; red block represents inversion and translocation; red arrow represents breakpoint.

Analysis of the basic structure of the assembled chromosomes revealed an inversion and translocation on chromosome 2 in both Hap1 and Hap2. We further studied the homologous chromosome of chromosome 2. Collinearity analysis revealed a ~ 10 Mb inversion and translocation on chromosome 2-1 compared to that on chromosomes 2-2, 2-3, and 2-4 (Fig. 3a). Since this has not been reported previously, we analysed whether this chromosomal structural variant exists. Collinearity analysis between chromosomes 2 of “Camarosa”, “Wongyo 3115”, “Royal Royce” and “Yanli” Hap1 and Hap2 revealed that the inversion and translocation existed on “Wongyo 3115” and “Royal Royce” chromosome 2–1, but not on “Camarosa” (Fig. 3b). Furthermore, the Hi-C heat map of chromosome 2–1 displayed the right interaction; however, when the continuous contig was disrupted and the segments were rearranged as in “Camarosa”, the Hi-C heat map showed the obvious breakpoint (location of red arrow), which represented the incorrect assembly (Fig. 3c). This suggested that the inversion and translocation on chromosome 2–1 exists. To further confirm this result, we downloaded “Camarosa” and “Wongyo 3115” genomic data from GDR (https://www.rosaceae.org/) and NCBI (https://www.ncbi.nlm.nih.gov/) databases and assembled the homologous chromosomes of chromosome 2 (Supplementary Data Fig. S8). Chromosome 2–1 of “Camarosa” was assembled using 270 contigs, each <1 Mb in length, displaying a fragmented chromosome assembly. On the contrary, chromosome 2–1 of “Yanli” consisted of one contig in both Hap1 and Hap2. Assembly of chromosomes with fragmented contigs possibly resulted in the loss of structural information of large chromosomal segments, leading to unsuccessful assembly of this structural variation. Moreover, the translocation on chr2–2 and chr2–3 in “Camarosa” and “Yanli” was possibly because of the same reason (Fig. 3b).

### Gene annotation of the “Yanli” genome and gene expression in different organs

Gene annotation includes structural and functional annotations. Homologue prediction of Hap1 with four relative species, including *Prunus avium*, *F. vesca*, *Malus **×** domestica*, and *Rosa chinensis,* annotated 113 191, 124 338, 115 173, and 134 862 genes, respectively. *De novo* prediction of the number of gene-coding proteins in Hap1 using the GlimmerHMM version 3.0.4 [32] and AUGUSTUS version 3.3.2 [33] yielded 124 383 and 88 667 genes, respectively. Using MAKER2 version 2.31.10 [34], the genes predicted using the above methods were integrated into a non-redundant and more complete gene set, and the final reliable gene set was obtained using the HiCESAP process. In total, 106 049 protein-coding genes were identified in Hap1, with average gene length of 4089 bp, whereas 103 213 protein-coding genes were identified in Hap2, with average gene length of 4243 bp (Supplementary Data Table S13). Untranslated regions (UTRs) were predicted in 55 298 genes in Hap1 and 55 502 genes in Hap2. Functional annotation showed that 88.81% and 89.69% of the proteins encoded by genes in Hap1 and Hap2 matched with the known proteins in public databases (Supplementary Data Table S14). BUSCO analysis showed 99.6% coverage of the embryophyta_odb 10 database in both Hap1 and Hap2 (Supplementary Data Table S15). Based on the structural characteristics of tRNA, tRNAscan-SE version 1.3.1 [35] was used to identify tRNA sequences in the genome. We selected rRNA sequences of closely related species as the reference sequence, and the rRNA in the genome was searched using BLASTN alignment. Rfam version 14.0 [36] was used to identify miRNAs and snRNAs sequence information in the genome. In total, 380 miRNAs, 5742 tRNAs, 6451 rRNAs, and 1353 snRNAs were annotated in Hap1 and 393 miRNAs, 3707 tRNAs, 7019 rRNAs, and 1331 snRNAs in Hap2, which represent 0.43% and 0.48% of the genome, respectively (Supplementary Data Table S16).

We collected the roots, shoot tips, mature leaves, and ripening fruits of “Yanli” and performed Illumina RNA-sequencing (Supplementary Data Table S17). Hap1 and Hap2 were selected as the reference genomes, respectively. Reads were aligned to the reference genome using STAR version 2.7.5a [37], transcripts were assembled using Stringtie version 1.3.5 [38], and the existing annotations were compared to obtain transcripts using Cuffcompare version 2.2.1 (http://cole-trapnell-lab.github.io/cufflinks/assets/downloads/cufflinks-2.2.1.tar.gz). Fragments per kilobase of exon per million mapped fragments (FPKM) [39] was used to detect the expression level of the protein-coding genes. The results showed that approximately 72% genes (75 199/104957 in Hap1 and 74 547/102356 in Hap2) were expressed in the root, shoot tip, leaf, or ripening fruit; among the expressed genes, 66% genes (49 236/75199 in Hap1 and 49 191/74547 in Hap2) were expressed in all four organs. Among the genes of Hap1, 1344 genes were expressed only in the leaf, 1753 only in the root, 4343 only in the shoot tip, and 2096 only in the ripening fruit, which were similar with those of Hap2 (Supplementary Data Fig. S9).

### Structural diversity and complexity in the expression of alleles in “Yanli” strawberry

*F.* × *ananassa*, arisen from four diploid species, is octoploid and usually cross-pollination, so the gene heterozygosity is higher. To investigate the structural diversity and complexity in the expression of alleles in the *F.* × *ananassa* genome, the genes related to anthocyanin biosynthesis pathway were analysed (Supplementary File 1).

**Figure 4:**
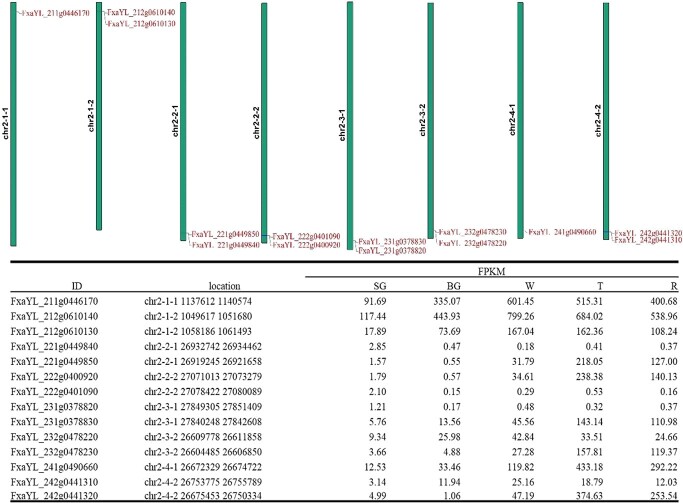
**Duplication of *FaDFR1* genes and their expression throughout fruit development.** SG **=** small green, BG = big green, W = white, T = turning, R = ripening.

We found that not all the alleles existed on the eight homologous chromosomes; for example, *FaCHS2*, *FaANS*, and *FaUFGT3* did not exist on chromosomes 7-2-1, 5-4-2, and 6-2-2, respectively, while, *FaF3H* only existed on five chromosomes, namely, chromosomes 7-1-1, 7-1-2, 7-3-1, 7-3-2, and 7-4-1 (Supplementary Data Figs. S10, S11, S12, and S13). Gene duplication occurred for some genes, which existed as gene clusters. For example, *FaDFR1* was duplicated in chromosome 2–1-2, 2–2-1, 2–2-2, 2–3-1, 2–3-2, and 2–4-2, but not on chromosomes 2–1-1 and 2–4-1 (Fig. 4). The lengths of the coding sequences (CDS) for the same genes of an allele differed; for example, the lengths of *FaDFR* were 1065 bp (FxaYL_311g0358350 on chromosome 3-1-1, FxaYL_312g0346790 on chromosome 3-1-2, FxaYL_322g0310330 on chromosome 3–2-2, and FxaYL_342g0201200 on chromosome 3-4-2), 819 bp (FxaYL_321g0320800 on chromosome 3-2-1), 411 bp (FxaYL_331g0205190 on chromosome 3-3-1), 972 bp (FxaYL_332g0274570 on chromosome 3-2-2), and 1047 bp (FxaYL_341g0279910 on chromosome 3–4-1) (Supplementary Data Fig. S14). The following might be responsible for the inter-allele differences in the length of the CDSs: (1) presence of insertions/deletions in CDS; (2) changes in translation start site; (3) changes in translation stop site. For all the genes related to the anthocyanin biosynthesis pathway, SNPs were ubiquitous.

To investigate the expression pattern of the alleles, we sequenced the whole transcriptomes of “Yanli” fruits at five different developmental stages, i.e. small green (SG), big green (BG), white (W), turning (T), and ripening (R) (Supplementary Data Table S17). The transcriptome data reflected the complex expression pattern of the alleles. For some alleles, few genes remained the dominant homologues throughout fruit development. For example, *FaMYB10* located on chromosome 1–2-1 (FxaYL_121g0707540) and chromosome 1–2-2 (FxaYL_122g0790550) represented 99.18% and 97.65% of the total *FaMYB10* expression in the T and R stages, respectively. However, the expression level of FxaYL_122g0790550 (represented 73.64% of total *FaMYB10* expression in the R stage) was higher than that of FxaYL_122g0790550 (represented 24.01% of total *FaMYB10* expression in R stage) (Supplementary Data Table S18). Two *FaANS* genes located on chromosome 5–1-1 (FxaYL_511g0695310) and chromosome 5–1-2 (FxaYL_512g0645780) represented 61.03% of the total *FaANS* expression in the R stage and were the dominant homologues throughout fruit development, while the genes located on chromosome 5–3-1 (FxaYL_531g0590000) and chromosome 5–3-2 (FxaYL_532g0366230) represented 36.89% of total *FaANS* expression in the R stage. The expression levels of FxaYL_511g0695310 and FxaYL_512g0645780, and those of FxaYL_531g0590000 and FxaYL_532g0366230 were similar (Supplementary Data Table S19). *FaCHI3* genes located on chromosome 7-2 (chromosomes 7-2-1 and 7-2-2), chromosome 7-3 (chromosomes 7-3-1 and 7-3-2), and chromosome 7-4 (chromosomes 7-4-1 and 7-4-2) represented 31.74%, 38.66%, and 26.71% of the total *FaCHI3* expression in the R stage, respectively, while genes located on chromosome 7-1 (chromosomes 7-1-1 and 7-1-2) only represented 2.89% of the expression (Supplementary Data Table S20). The expression levels of the duplicated genes were low; for example, FxaYL_731g0856620 (*FaCHI1*), duplicated from FxaYL_731g0856630, was not transcribed throughout fruit development (Supplementary Data Table S21), while the expression level of FxaYL_221g0449840 (*FaDFR1*) was only 0.29% (0.37/127.00) of that of the original gene, FxaYL_221g0449850 (Fig. 4).

## Discussion

Polyploid organisms have multiple alleles compared with diploid organisms having two alleles at the same locus, which complicates haplotype identification efforts in polyploids [40]. To better resolve haplotypes in polyploid genomes, efficient chromosome set separation techniques can be used before genotyping. After decades of attempts, researchers have successfully isolated sets of homologous chromosomes in allopolyploids using methods that rely on novel DNA sequencing technology such as HiFi and Hi-C [41, 42]. However, efficient sequence separation in autopolyploid genomes remains a challenge due to the high degree of similarity between each homologous chromosome group and possible meiotic recombinants [41]. Cheng *et al*. assembled a 3.1 Gb diploid ginger genome and performed haplotype analysis, and they found that two chromosomal inversions larger than 15 Mb on a chromosome may be associated with sterility in ginger [42]. Moreover, Li *et al*. obtained a chromosome-scale genome of ginger using SMRT and Hi-C technologies and divided it into haplotype 1 and haplotype 0 [43]. In addition, haplotype analysis has been performed for crops such as potato and tea, for which chromosome-level genomes have been identified [44–46]. Here, for the first time, we performed a high-quality chromosome-scale assembly of the cultivated octoploid strawberry (*F. × ananassa*) “Yanli” genome using SMRT technology and separated the genome into two haplotypes named Hap1 and Hap2. Unlike studies on ginger, we did not find any obvious chromosomal structural variation between the two haplotypes of the “Yanli” genome. At the same time, high collinearity was observed between the two haplotype chromosomes, and the related parameters of gene annotation were identical. This indicated that our haplotype phasing of the octoploid strawberry genome was successful and that it can provide a basis for follow-up studies.

Chromosomal structural variations play an important role in plant evolution, and the emergence of new species is often accompanied by changes in chromosome structure, such as translocations and inversions [47]. Heterozygous chromosomal structural variants produce unbalanced gametes during meiosis, resulting in gamete sterility and reproductive isolation [48]. The combination of two homologous genes can increase the probability of chromosomal rearrangements, thereby improving the adaptability of species to the environment [49]. Chromosomal inversions are associated with polymorphisms in many species and can locally reduce recombination [50]. Hybridization is an important way of generating chromosomal structural variations [51]. Wheat-rye hybridization induced alterations in wheat chromosomes 5A, 6A, 1B, 2B, 6B, 1D, and 3D [52]. Chromosomal rearrangements exist in many cultivated banana genomes that are hybrids between *Musa acuminata* and *M. balbisiana* [53]. Similar to wheat, cultivated octoploid strawberry is allopolyploid, and chromosome doubling occurs due to a hypoploid hybridization event; thus, chromosome structural variations can be generated in this process. In this study, we reported for the first time a ~ 10 Mb inversion and translocation on chromosome 2–1 both in Hap1 and Hap2. Similar to that in the reported cultivated octoploid strawberry genome, this inversion and translocation exists in “Wongyo 3115” [12], but not in “Camarosa” [11]. We speculated that although this inversion and translocation existed in the “Camarosa” genome, it was not discovered probably because of using Illumina sequencing technology. In this study, the genome of “Yanli” was assembled with the third-generation SMRT sequencing technology. Compared to second-generation Illumina technology, which generates reads of 150 bp average length, SMRT sequencing technology yielded reads with average read length of about 20 000 bp, which preserved the chromosome’s structural information [54, 55].

In cultivated strawberry, trait variation at a single locus may be controlled by up to eight homoeoalleles [56]. In the high-quality haplotype-resolved genome of “Yanli”, we found that not all the alleles were present on the eight homologous chromosomes; for example, *FaF3H* has only five homoeoalleles. We also found that many genes were duplicated in the genome of “Yanli”, and that the length of the CDS of the alleles varied abundantly. All these reflect the complexity of alleles in allopolyploids [57, 58]. Genomic variants affect the dosage of homologous gene expression, which contributes to agronomic trait variation in allopolyploids [59]. In this study, we found that some genes were the dominant homologues throughout fruit development. For example, few *FaMYB10* genes located on chromosome 1-2 (chromosomes 1-2-1 and 1-2-2) represented 97.65% of the total *FaMYB10* expression in the R stage, which is consistent with that reported previously [60]. However, in the haplotype-resolved genome of cultivated strawberry, we observed that the expression dosage of the two *FaMYB10* genes located on chromosomes 1-2-1 and 1-2-2 differed considerably. The expression level of FxaYL_122g0790550 (located on chromosome 1-2-2) represented 73.64% of the total *FaMYB10* expression in the R stage, while FxaYL_122g0790550 (located on chromosome 1-2-1) represented 24.01% of the total *FaMYB10* expression in the R stage. Although the identity of FxaYL_122g0790550 and FxaYL_122g0790550 CDS is 100%, *FaMYB10* located on chromosome 1-2-2 is the actual dominant homologue in cultivated strawberry. This information will be beneficial for understanding the mechanism underlying *FaMYB10* transcription.

In summary, we obtained a high-quality haplotype-resolved genome assembly of cultivated octoploid strawberry, which will provide a solid foundation for future studies on gene function and genome evolution. However, the reason for chromosome structural variation on chromosome 2-1 and the reasons for structural diversity and expression complexity of the alleles in the octoploid *F. × ananassa* genome need to be further studied.

## Materials and methods

### Plant materials

The cultivated strawberry (*Fragaria* × *ananassa*) cultivar “Yanli” (2*n =* 8*x =* 56) was used for genome sequencing. “Yanli” was grown in the solar greenhouse of the Shenyang Agriculture University. The leaves for genome sequencing were collected in August, 2021. Shoot tips for RNA-sequencing were collected in June, 2018, while roots, leaves, and fruits for RNA-sequencing were collected in February, 2021. All materials were stored at −80°C.

### Genomic DNA extraction

Mature leaves were used to extract genomic DNA of “Yanli” using the cetyltrimethylammonium bromide (CTAB) method [61]. The quality and concentration of DNA was determined using 1% agarose gel electrophoresis and a Qubit 3.0 fluorometer (Life Technologies, Carlsbad, CA, USA).

### Illumina sequencing and genome analysis

DNA fragmentation was performed using ultrasonic processor, such that the length of the insert fragments was approximately 350 bp. Then terminal repair, addition of base A, addition of sequence adapter, purification, and PCR amplification were performed to prepare a library of 350 bp fragments. After quantification using Qubit 2.0, the library was diluted to 1 ng/μL. Then, the insert size of the library was verified using Agilent 2100. And the effective concentration of the library was determined by quantitative PCR (qPCR).

Illumina Novaseq 6000 was used for paired-end sequencing. The raw data was processed using FastQC version 0.11.3 [62] to remove adapters, and the leading and trailing ambiguous or low quality bases. Clean data was used for further genome analysis. GCE version 1.0.0 [63] was used to generate a k-mer (k = 17) depth distribution curve. The “Yanli” genome size (GS) was calculated using the follow formula: GS = k-mer number/average k-mer depth. The formula described by Fahad *et al*. was used to estimate the heterozygosity of the “Yanli” genome [64].

### PacBio SMRT sequencing and assembly

After the genomic DNA used for building SMRT library was extracted, PacBio’s standard protocol (Pacific Biosciences, CA, USA) was used to build SMRTbell target size libraries. The library was sequenced using PacBio Sequel II with primer V2 and Sequel II binding kit 2.0. The *de novo* assembly was performed using Hifiasm version 0.16.1 [65].

### Hi-C assembly

The Illumina HiSeq X Ten platform was used to construct the Hi-C library by anchoring configs onto the chromosome. Qubit 2.0 and Agilent 2100 were used to determine the concentration and insert size. HiCUP [66] was used to process sequence data generated by Hi-C and 3d-DNA [67] was used to assist assembly of genome. After the Hi-C interaction heatmap matrix was constructed by Juicer version 1.5.6 [68], mis-joins, order, and orientation were corrected by JuiceBox version 1.11.08 [69]. And the reads were aligned to the genome by Bowtie 2 [70].

### Evaluation of assembly quality

The second and third generation data were aligned using bwa-mem version 0.7.12 [71] and minimap2 version 2.22 [72]. Genome integrity was assessed using LAI obtained using LTR_FINDER version 1.0.7 [73] and LTR_retriever [74]. Homozygous SNP and InDel rate were measured using GATK version 4.2.0.0 [75]. The completeness and accuracy of the genome assembly were evaluated using CEGMA version 2.5 [76] and BUSCO version 5.3.1 [29].

### Annotation of repetitive sequences

Repetitive sequences were annotated using a combination of homology prediction methods and *de novo* prediction methods. RepeatScout version 1.0.5 [77], RepeatModeler version 2.0.1 [78], and PILER version 1.0 [79] were used for self-sequence alignment, while Trf version 4.10.0 [80] and LTR_FINDER version 1.0.7 [73] were used for recognizing repetitive sequence features. The predicted repeats were classified using PASTE version 1.0 [81]. The final database of repeats was obtained by combining the predicted repeats with Repbase database version 19.06 [82] of repetitive DNA elements. Finally, RepeatMasker program version 4.0.9 [83] was used to identify repeat sequences by aligning them against the final repeat database.

### Gene perdition and functional annotations

A combination of homologue prediction, *de novo* prediction, and RNA-seq/EST prediction was used to annotate protein-coding genes in the “Yanli” genome. Sequences of *P. avium*, *F. vesca*, *Malus* × *domestica*, *R. chinensis*, and *F.* × *ananassa* and Exonerate version 2.2.0 were used for predicting homologous genes [84]. *De novo* prediction was measured using AUGUSTUS version 3.3.2 [33] and GlimmerHMM version 3.0.4 [32]. Splice junctions between exons were identified in the RNA-seq data using TopHat version 2.0.4 [85] and assembled into transcripts using Cufflinks version 2.2.1. All the predictions made using the three methods were combined using MAKER2 version 2.31.10 [34] to generate non-redundant and more complete gene sets. HiCESAP was used for obtaining final reliable gene sets. Exogenous protein databases, including nonredundant protein database, SwissProt, TrEMBL, Kyoto Encyclopaedia of Genes and Genomes (KEGG), and Eukaryotic Orthologous group (KOG) were used to assign Gene functions after BLASTP [86]. The domain information was obtained using InterProScan version 4.3 [87]. The Gene Ontology (GO) database and Blast2GO were also used for annotating function. Finally, the predicted genes were subjected to KOG functional enrichment analysis, KEGG pathway enrichment analysis, and GO functional enrichment analysis [88].

### Prediction of noncoding RNA

Whole transcriptome sequencing of the roots, leaves, flowers, and fruits was performed for predicting noncoding RNAs. tRNAscan-SE version 1.3.1 [35] was used to predict tRNAs based on the structural characteristics. As rRNAs are highly conserved, the rRNA sequences of the closely related species can be used as reference sequences and rRNAs can be annotated using BLASTN alignment. In addition, Rfam version 14.0 [36] was used to identify miRNAs and snRNAs in the assembled “Yanli” genome.

### RNA sequencing and gene expression analysis

We collected the samples of roots, shoot tips, mature leaves, and different developmental stage fruits of “Yanli”. CTAB method was used to extract total RNA from different organs, and QIAGEN® Genomic kit (Cat#13343, QIAGEN) was used to purify RNA samples. RNA purity and concentration were checked with Nanodrop 2000 and 1% agarose gel electrophoresis. The library was constructed with high quality RNA using NEBNext Ultra™ RNA Library Prep Kit for Illumina (NEB, USA). The first strand of cDNA was synthesized with random hexamer primer and M-MuLV reverse transcriptase, and the second strand of cDNA was subsequently obtained using DNA Polymerase I and RNase H. AMPure XP system (Beckman Coulter, Beverly, USA) was used to purify the library fragments to ensure selection ~240 bp cDNA fragments. The Illumina Hiseq 2000 platform was used to sequence the library and generate the paired-end reads.

STAR version 2.7.5a [37] was used to align the reads to the reference genome and the transcripts were assembled using Stringtie version 1.3.5 [38]. Then, Cufflinks version 2.2.1 was used to compare the existing annotations, obtain new transcripts, and add protein-coding transcripts identified using CPC2 (beta) [89] to the gene set. HTSeq version 0.12.4 [90] was used to count the reads that mapped to each gene. Then, the fragments per kilobase of exon per million mapped fragments (FPKM) [29] of each gene was calculated based on the length of the gene and the read counts that mapped to this gene.

### Allele identification and analysis

Tbtools was used for homology and collinearity analysis of alleles with default settings [91]. The location of alleles was visualized using Tbtools. DNAMAN v8.0.8.789 was used for multiple sequence alignment.

## Acknowledgements

We thank Wuhan Onemore-tech Co., Ltd. for their assistance with genome sequencing and analysis. This work was financially supported by National Natural Science Foundation of China (No. 32130092; No. 31872072) and LiaoNing Revitalization Talents Program (No. XLYC1902069).

## Author Contributions

Y.W. and Z.Z. conceived and designed the study. Y.W. and J.M. prepared the materials. B.W., J.L., C.Z, W.Z., X.L., J.L., J.Z. and H.L. performed the bioinformatics analysis and prepared the results. J.M. and Y.W. wrote the manuscript. Z.Z. edited and improved the manuscript. All authors approved the final manuscript.

## Data availability

The data that support the results are included in this article and its supplementary materials. Other relevant materials are available from the corresponding author upon reasonable request.

## Conflict of interest

The authors declare no competing interests.

## Supplementary data

Supplementary data is available at *Horticulture Research* online.

## Supplementary Material

Web_Material_uhad002
