# Supplementary material for: High-quality haplotype-resolved genome assembly of cultivated octoploid strawberry
Source: Hortic Res. 2023 Jan 4;10(1):uhad002. doi: 10.1093/hr/uhad002 (PMC10108017; doi:10.1093/hr/uhad002)
Supplement: Web_Material_uhad002 [file web_material_uhad002.zip › Supplementary Figures.docx]

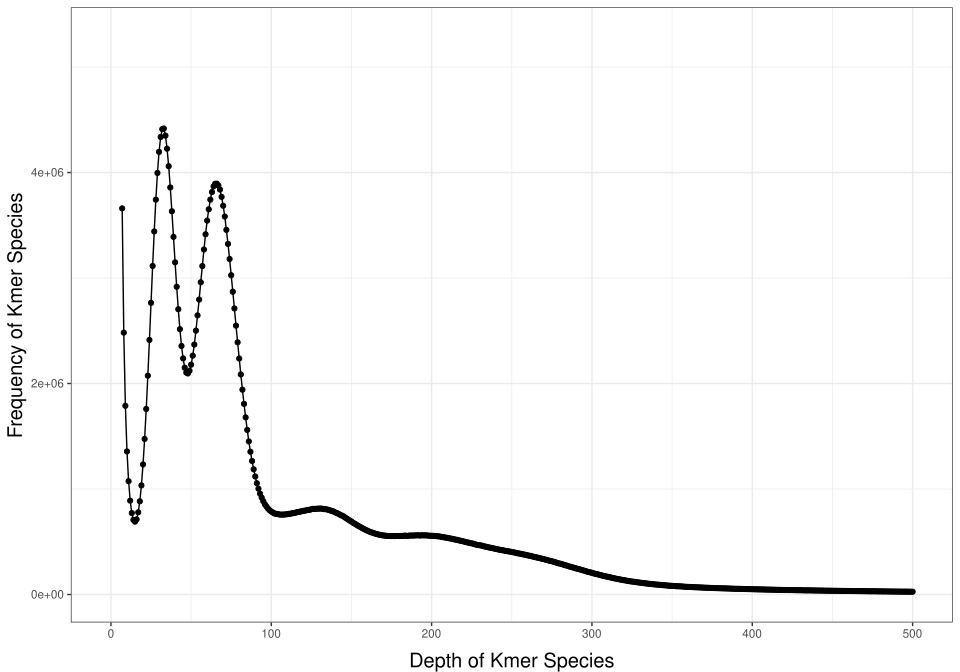


**Figure S1. Frequency distribution of K-mer Depth and K-mer species**

**
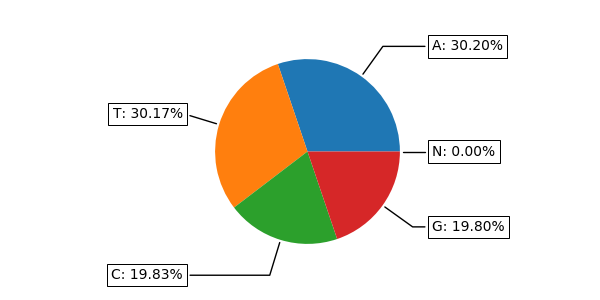
**

**Figure S2 statistics of nucleotide composition distribution.**

**
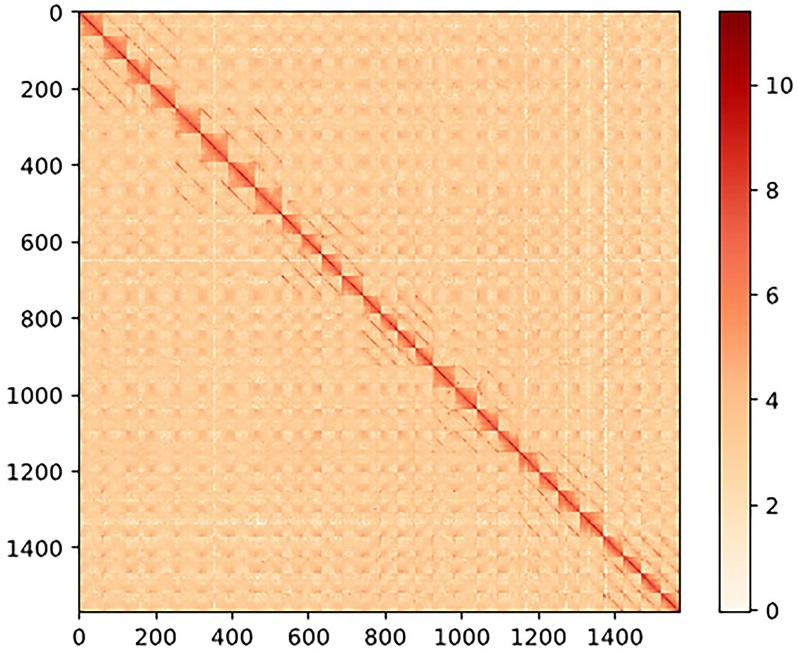

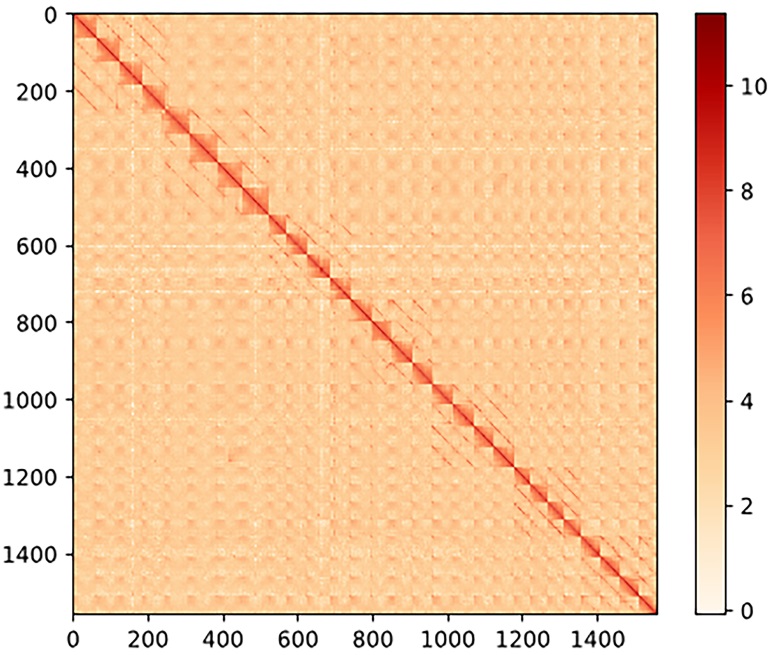
**

**Figure S3 Chromosomal Hi-C interaction heat map of Hap1 and Hap2 (resolution ratio = 500k)**


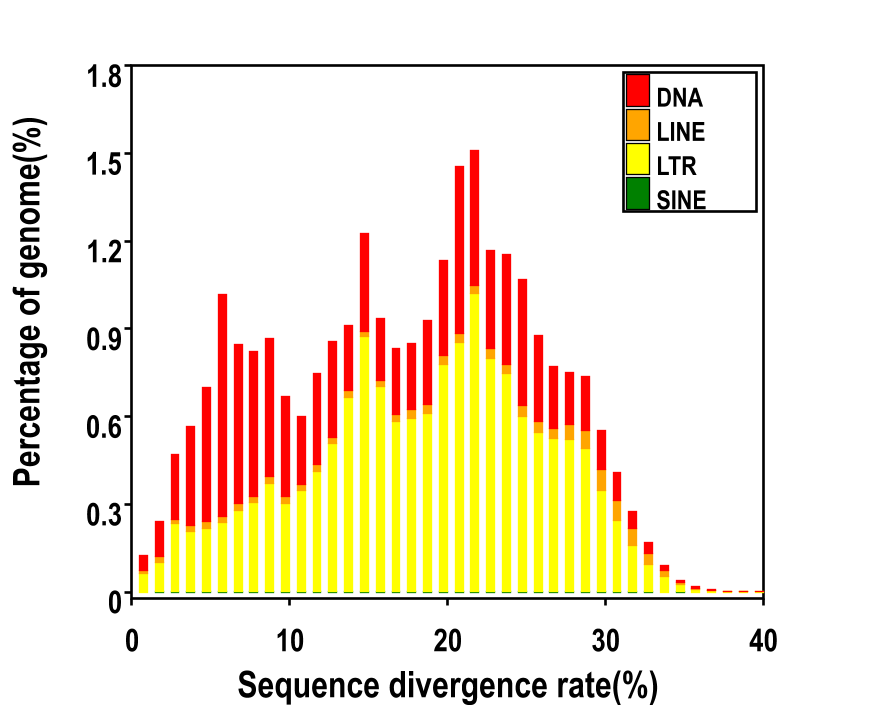


**Figure S4. Distribution map of divergence degree of four TE sequences annotated by RepeatMasker**


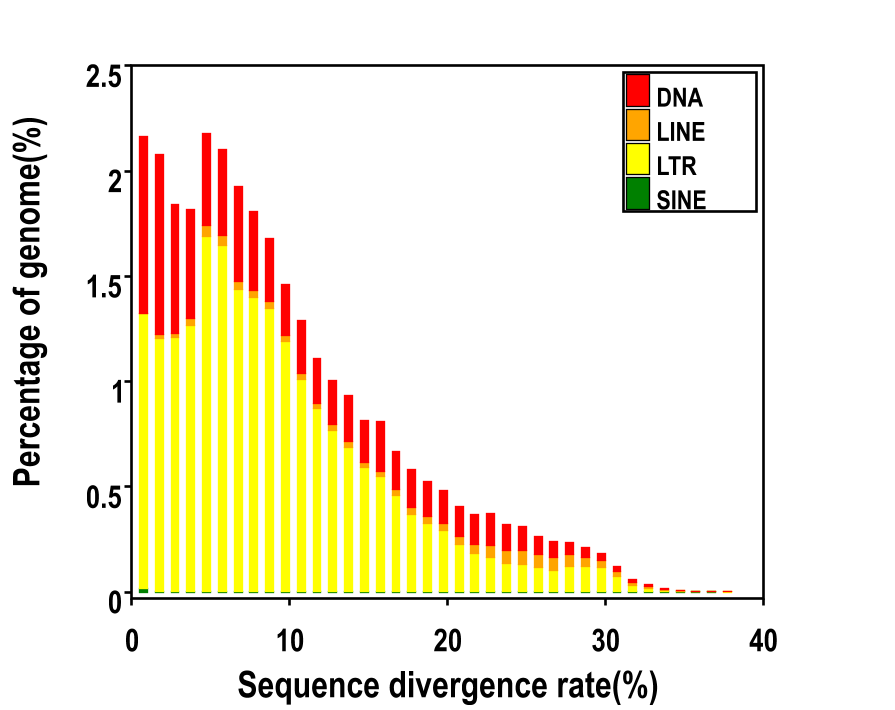


**Figure S5. Distribution map of divergence degree of four TE sequences annotated by *De novo***


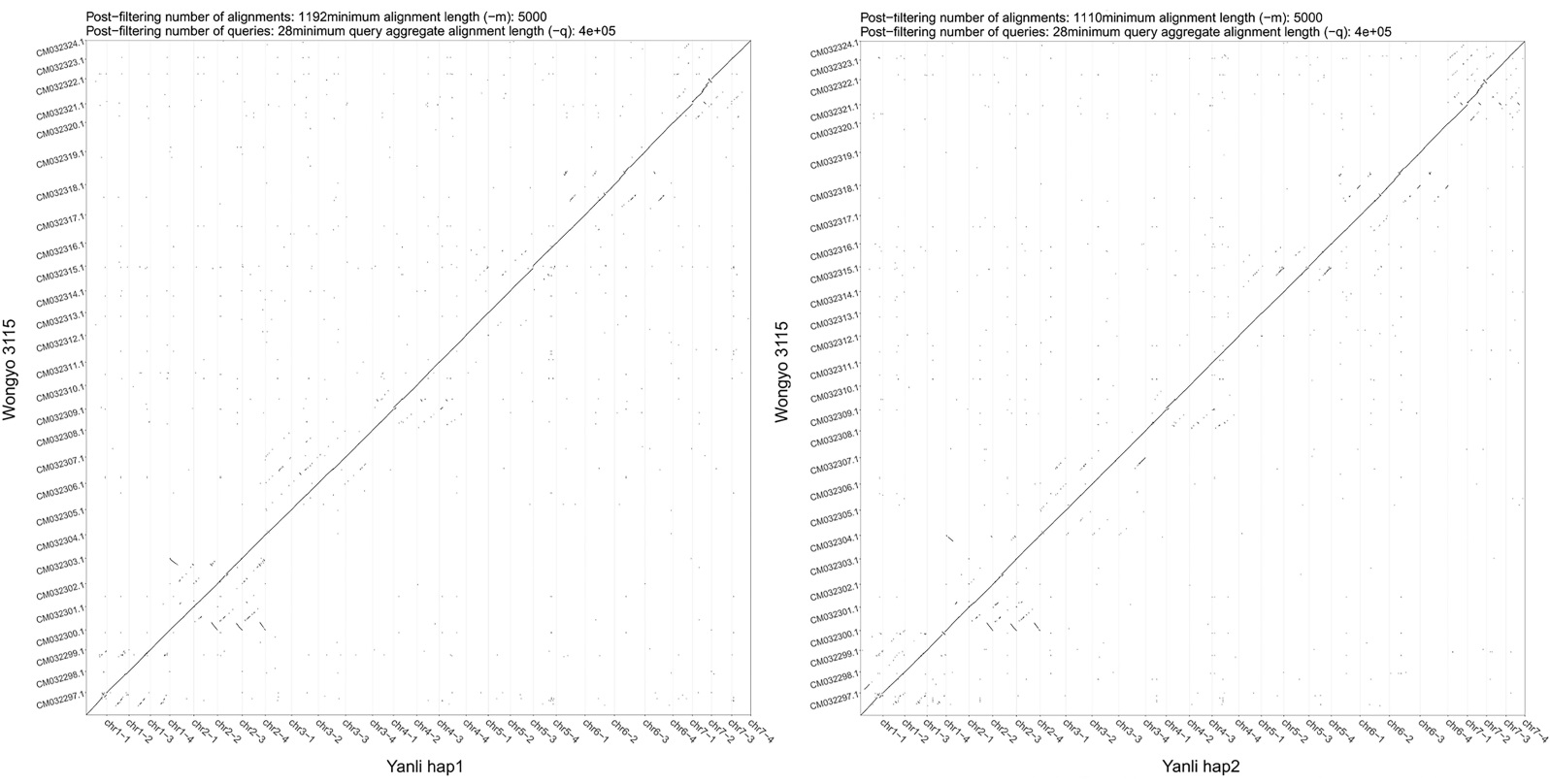


**Figure S6. Collinearity analysis between Hap 1, Hap 2 and Wongyo 3115**

**
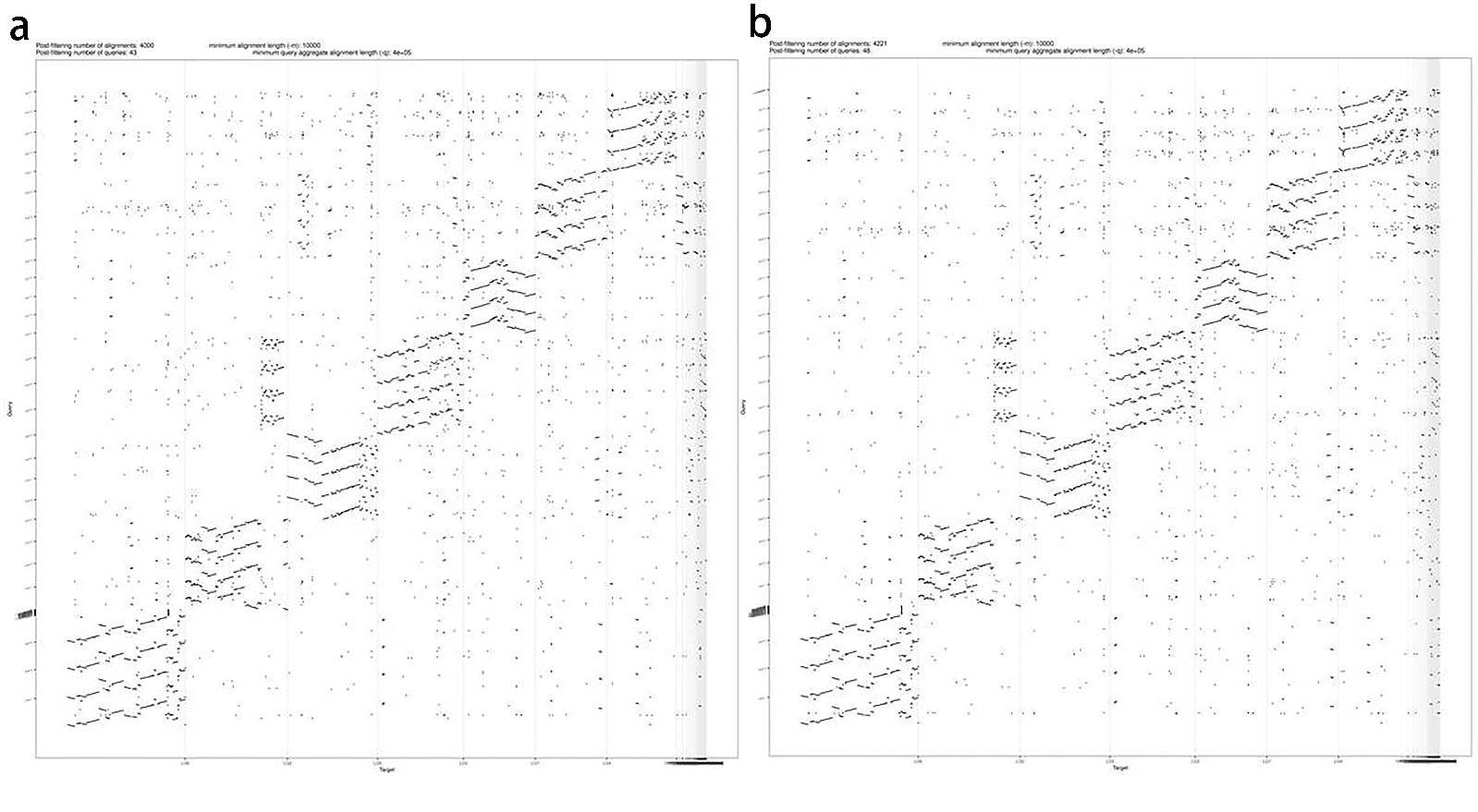
**

**Figure S7. Collinearity analysis between Hap 1, Hap 2 and Hawaii 4**

**
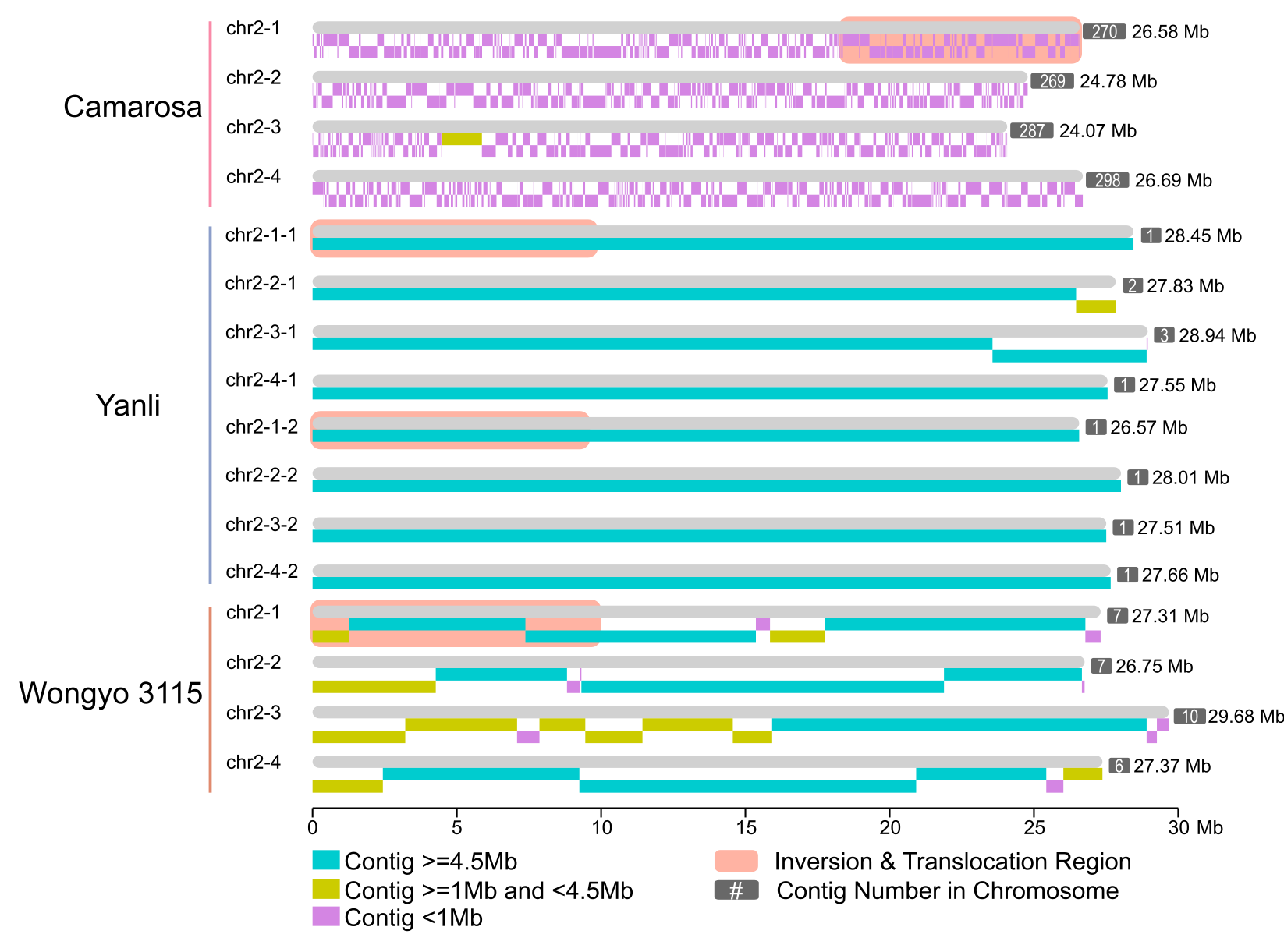
**

**Figure S8. Assembly of chromosome 2-1, 2-2, 2-3, and 2-4**

**
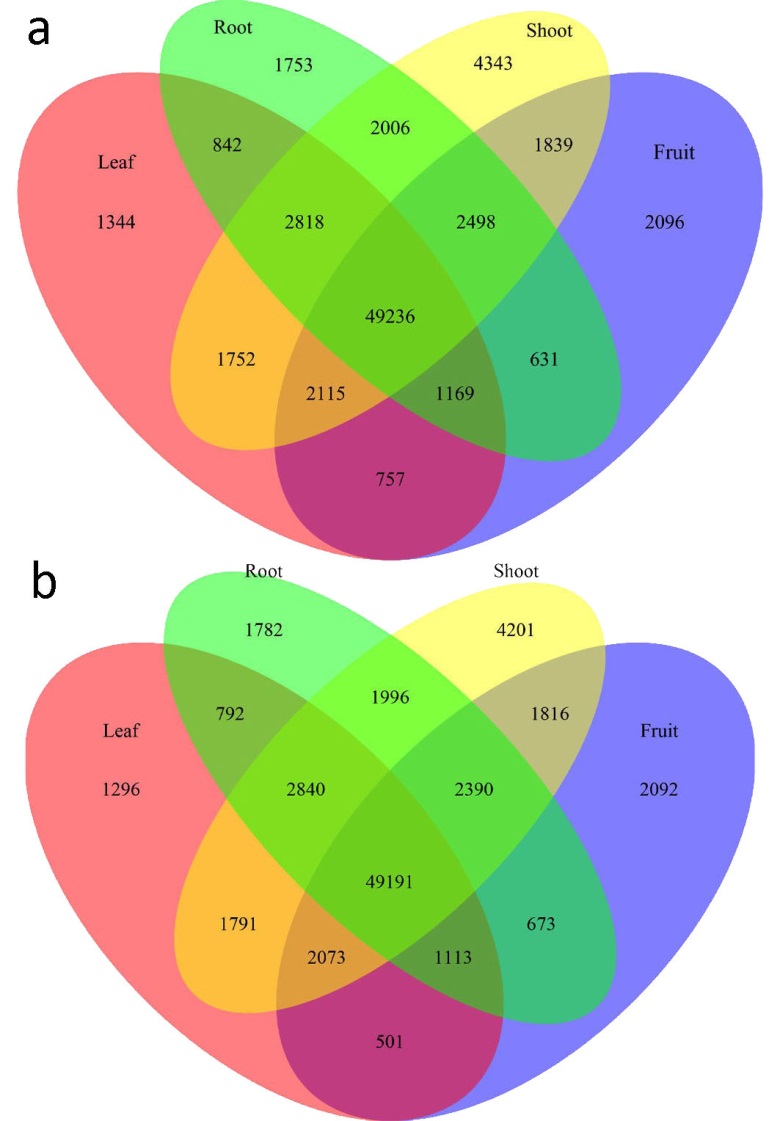
**

**Figure S9. Venn diagram showing genes in the leaf, root, shoot, and fruit.**

**
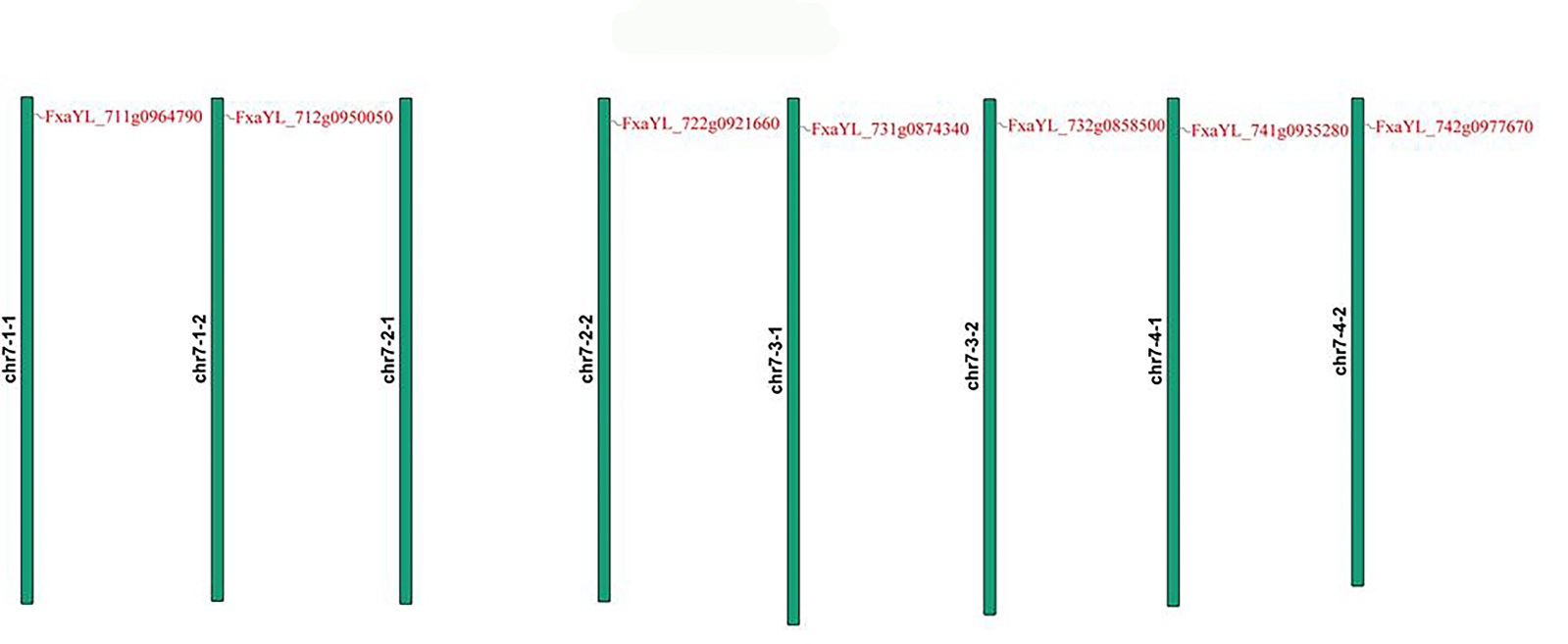
**

**Figure S10. Location of *FaCHS2* gene**

**
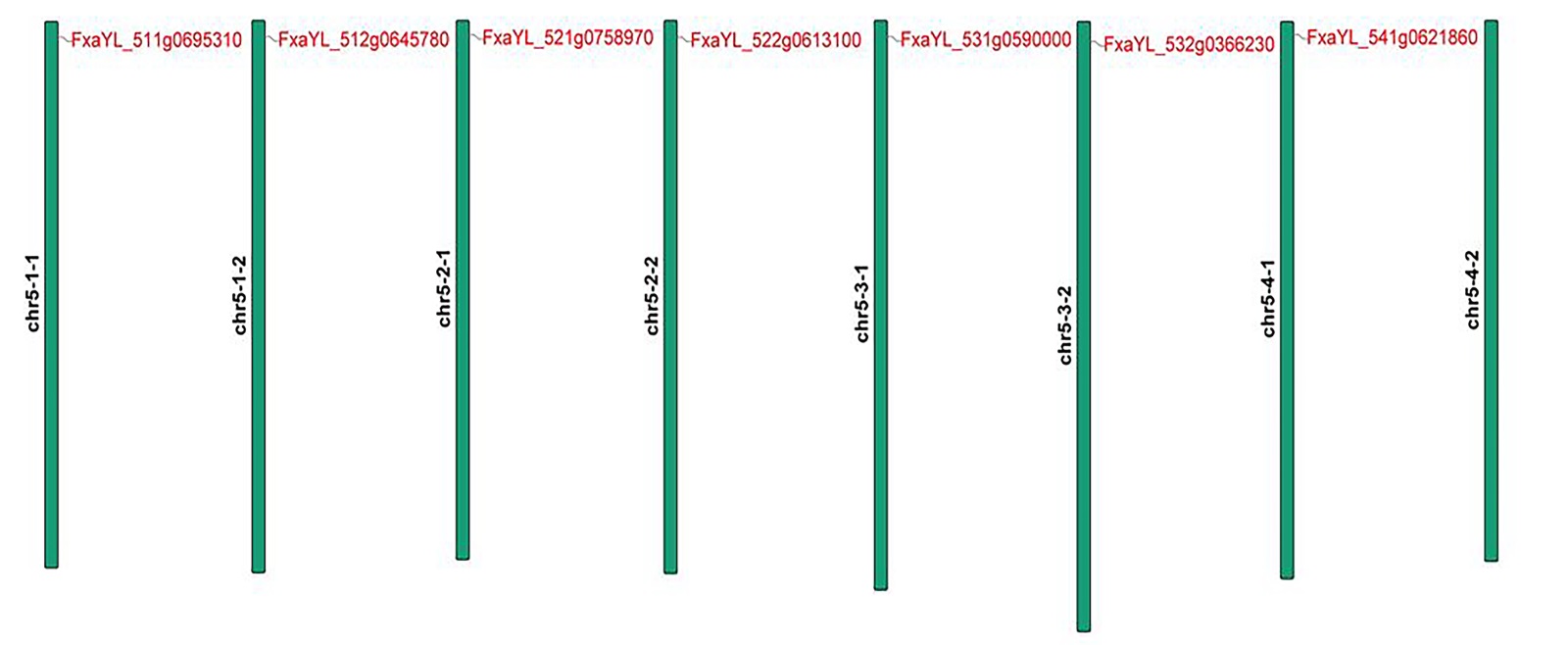
**

**Figure S11. Location of *FaANS* gene**

**
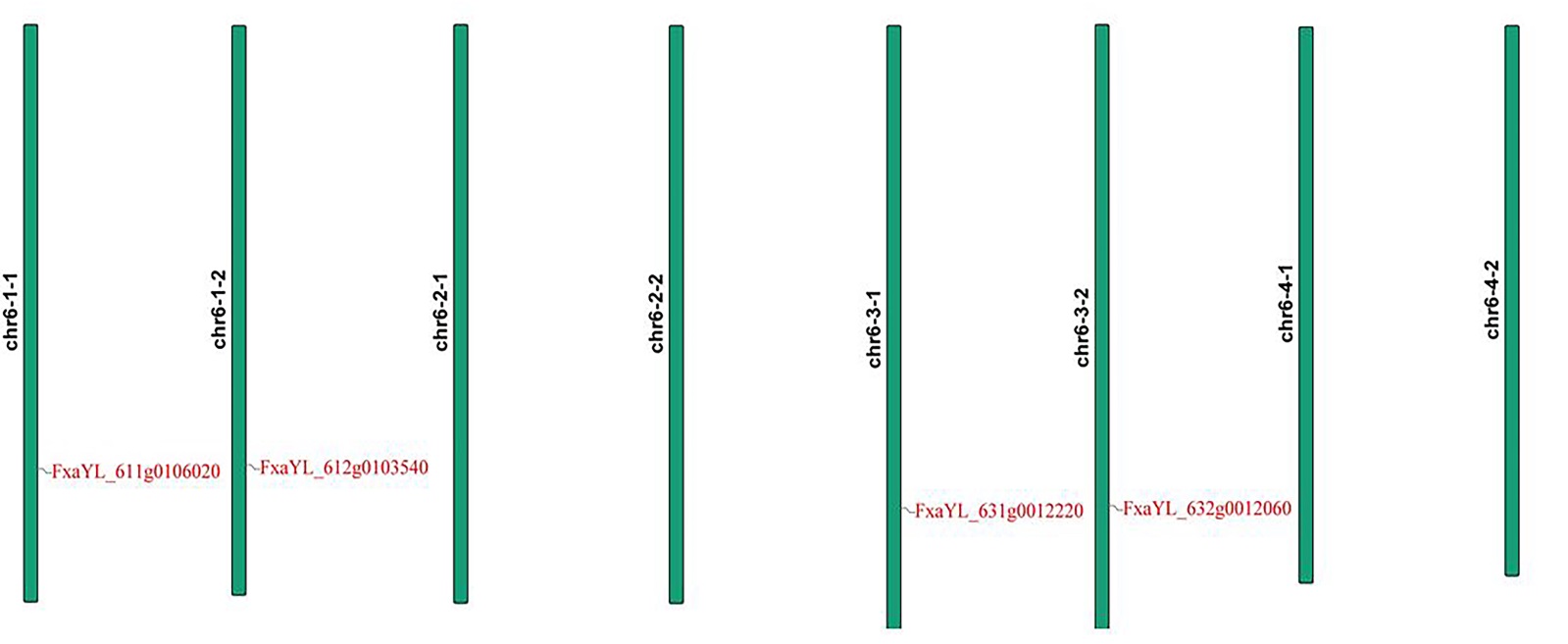
**

**Figure S12. Location of *FaUFGT****3* **gene**

**
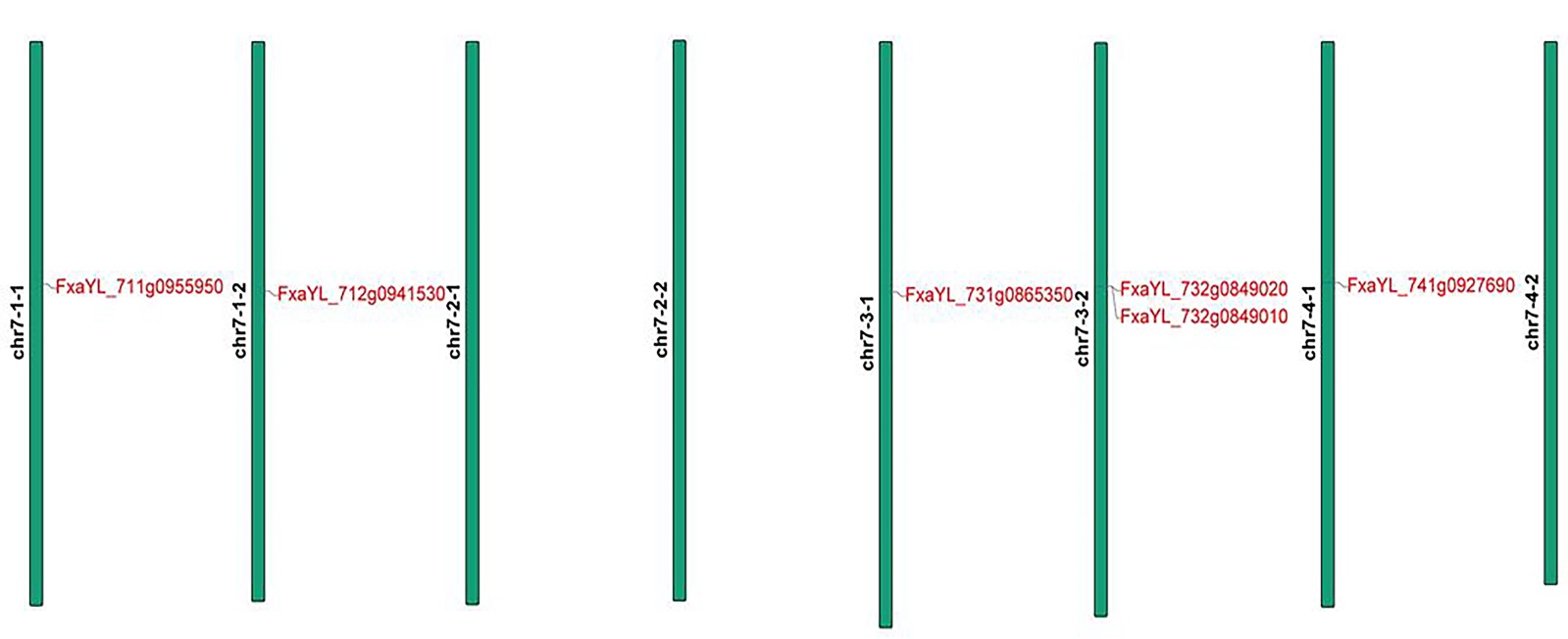
**

**Figure S13. Location of *FaF3H* gene.**

**
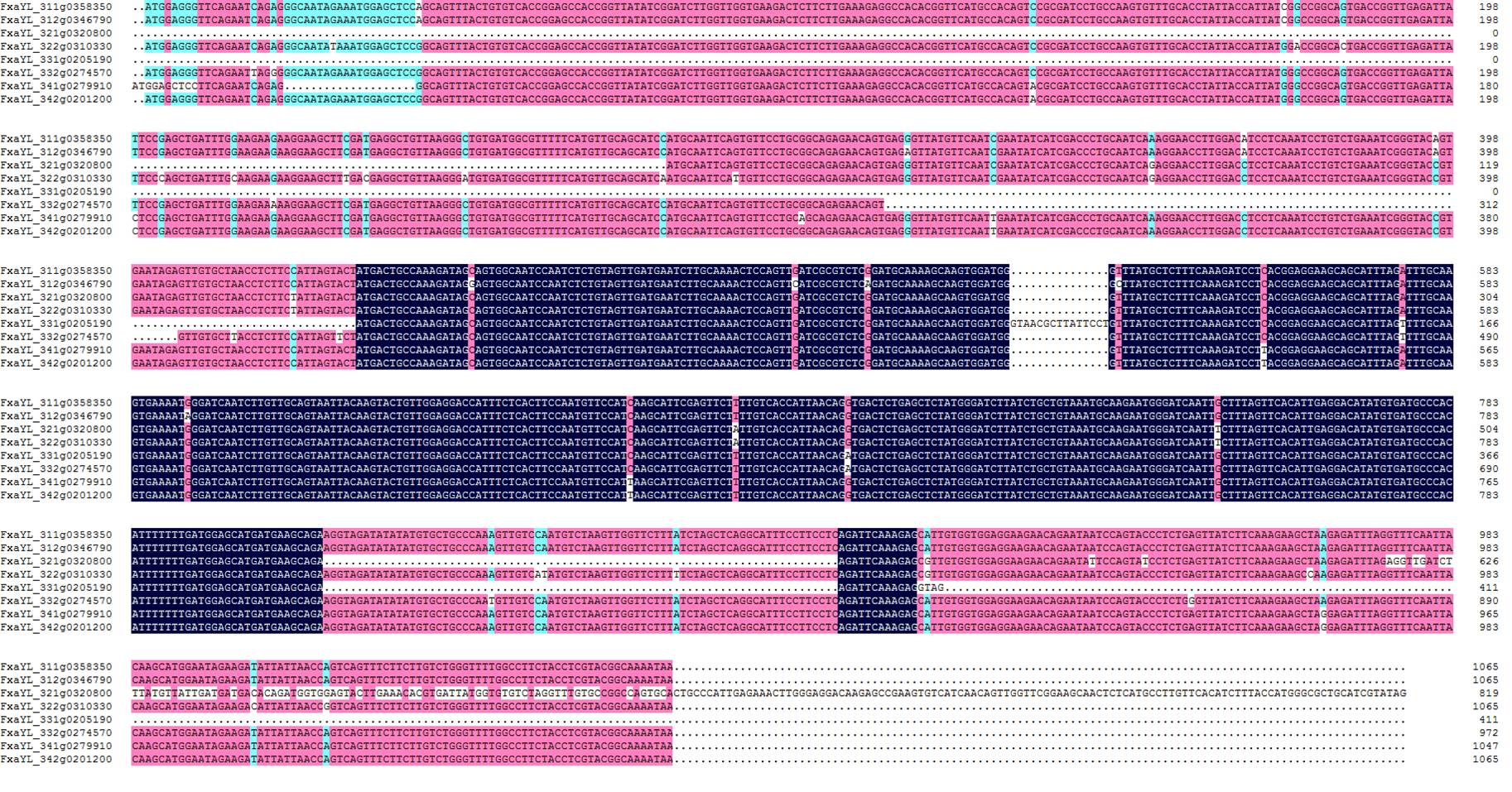
**

**Figure S14. Different length of coding sequence for *FaDFR***
