# Supplementary material for: High-quality haplotype-resolved genome assembly of cultivated octoploid strawberry
Source: Hortic Res. 2023 Jan 4;10(1):uhad002. doi: 10.1093/hr/uhad002 (PMC10108017; doi:10.1093/hr/uhad002)

## Slide 1
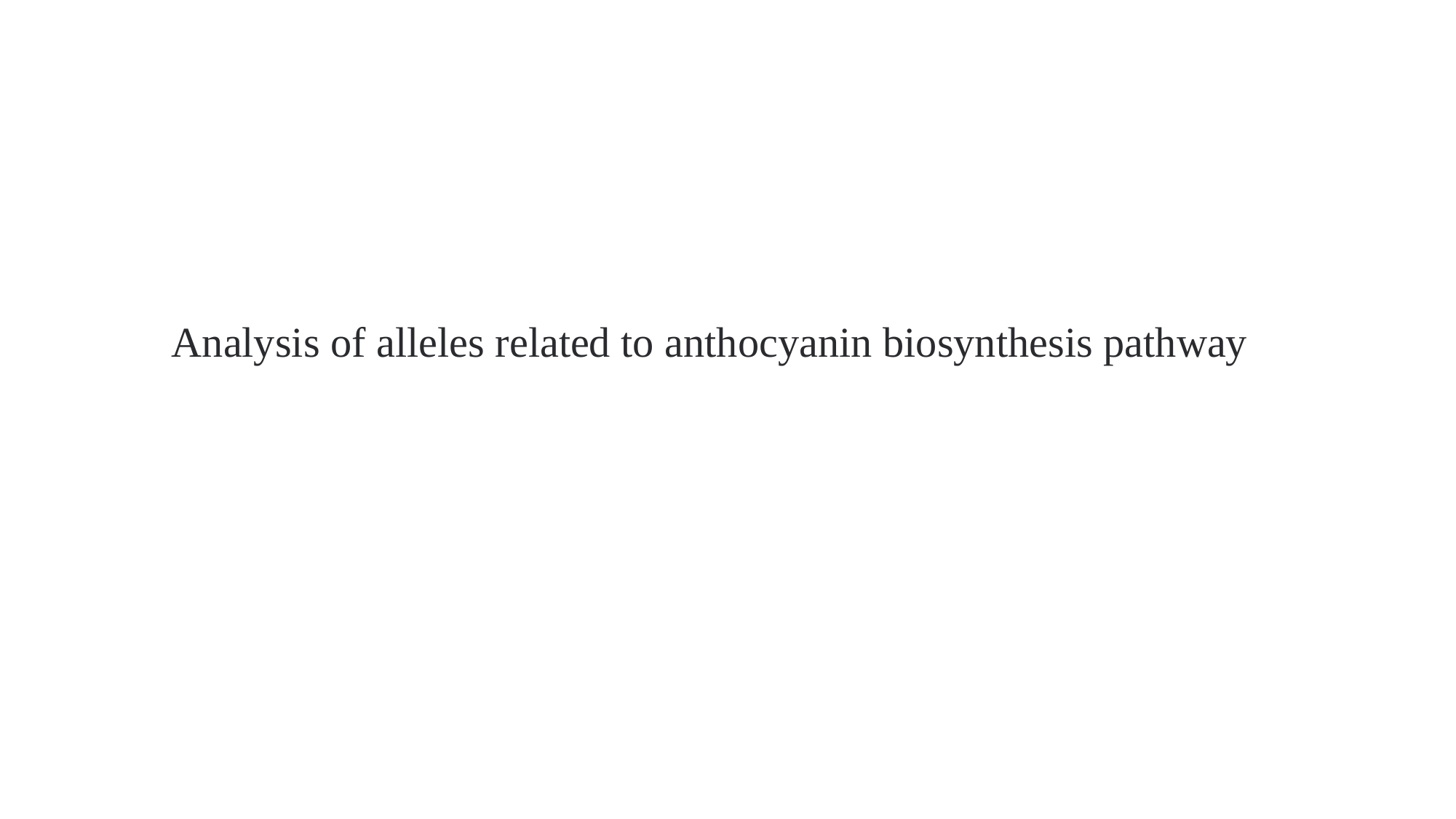

Analysis of alleles related to anthocyanin biosynthesis pathway

## Slide 2
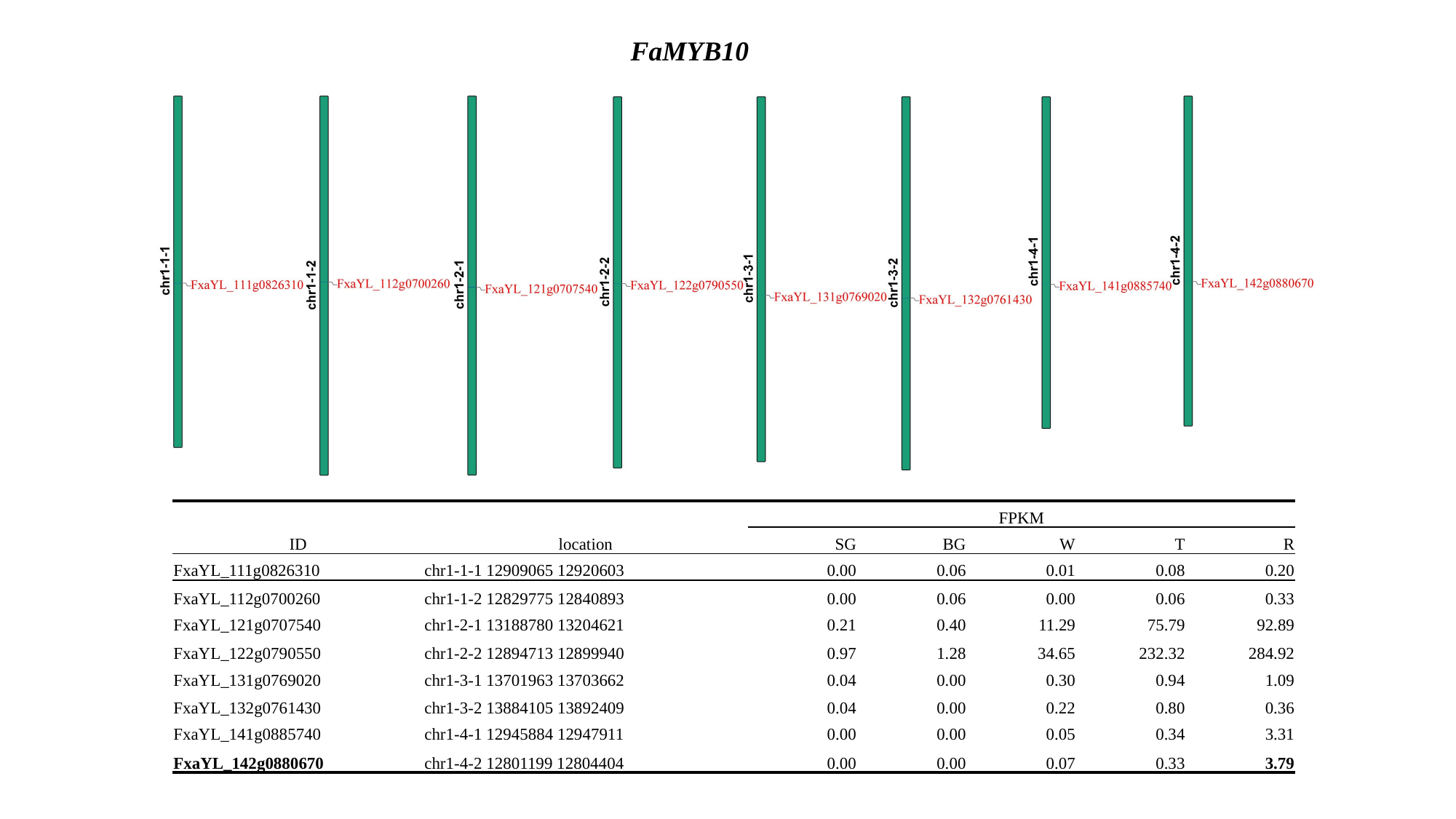

FaMYB10
| | | FPKM | | | | |
| --- | --- | --- | --- | --- | --- | --- |
| ID | location | SG | BG | W | T | R |
| FxaYL\_111g0826310 | chr1-1-1 12909065 12920603 | 0.00 | 0.06 | 0.01 | 0.08 | 0.20 |
| FxaYL\_112g0700260 | chr1-1-2 12829775 12840893 | 0.00 | 0.06 | 0.00 | 0.06 | 0.33 |
| FxaYL\_121g0707540 | chr1-2-1 13188780 13204621 | 0.21 | 0.40 | 11.29 | 75.79 | 92.89 |
| FxaYL\_122g0790550 | chr1-2-2 12894713 12899940 | 0.97 | 1.28 | 34.65 | 232.32 | 284.92 |
| FxaYL\_131g0769020 | chr1-3-1 13701963 13703662 | 0.04 | 0.00 | 0.30 | 0.94 | 1.09 |
| FxaYL\_132g0761430 | chr1-3-2 13884105 13892409 | 0.04 | 0.00 | 0.22 | 0.80 | 0.36 |
| FxaYL\_141g0885740 | chr1-4-1 12945884 12947911 | 0.00 | 0.00 | 0.05 | 0.34 | 3.31 |
| FxaYL\_142g0880670 | chr1-4-2 12801199 12804404 | 0.00 | 0.00 | 0.07 | 0.33 | 3.79 |

## Slide 3
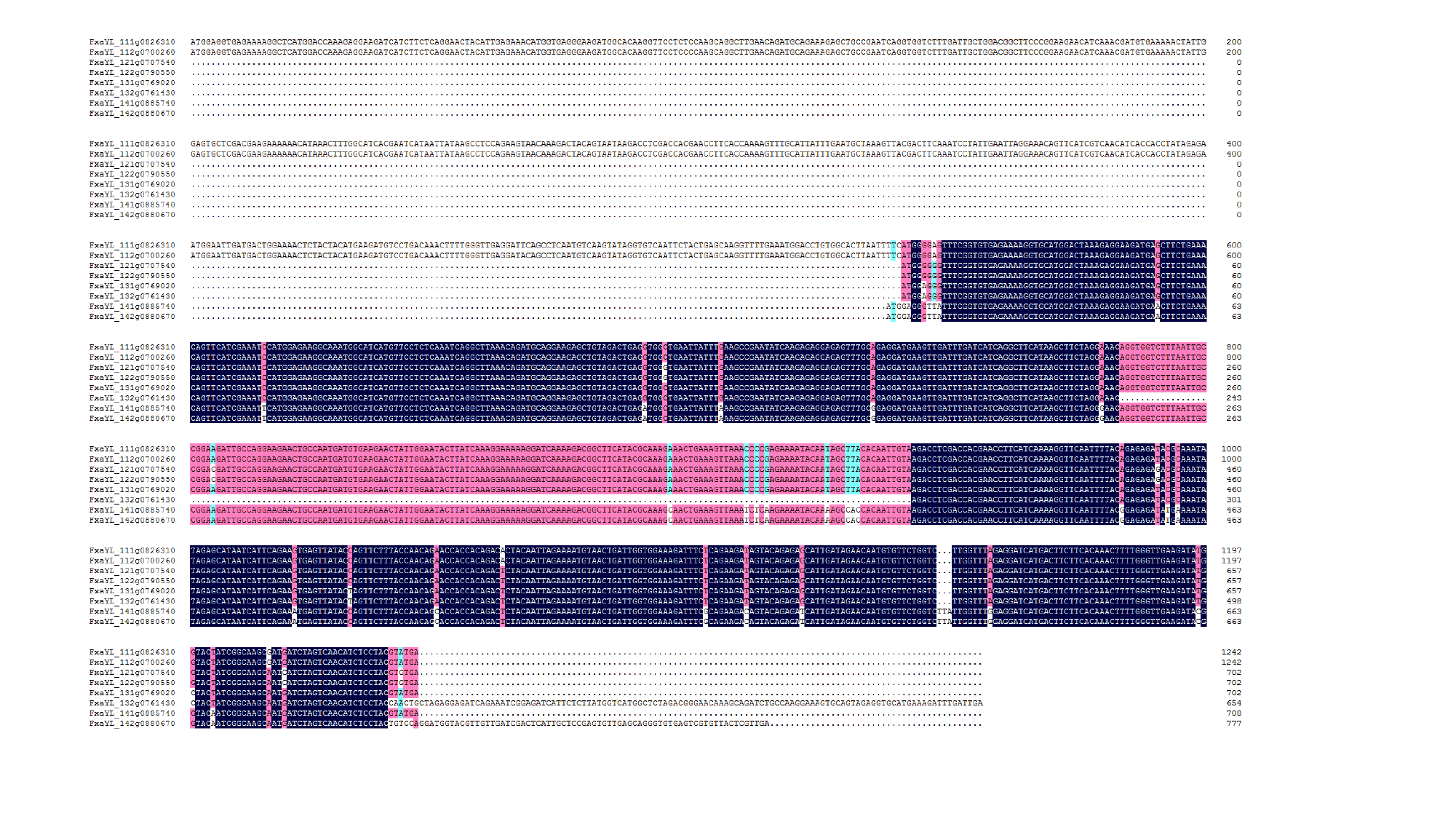

## Slide 4
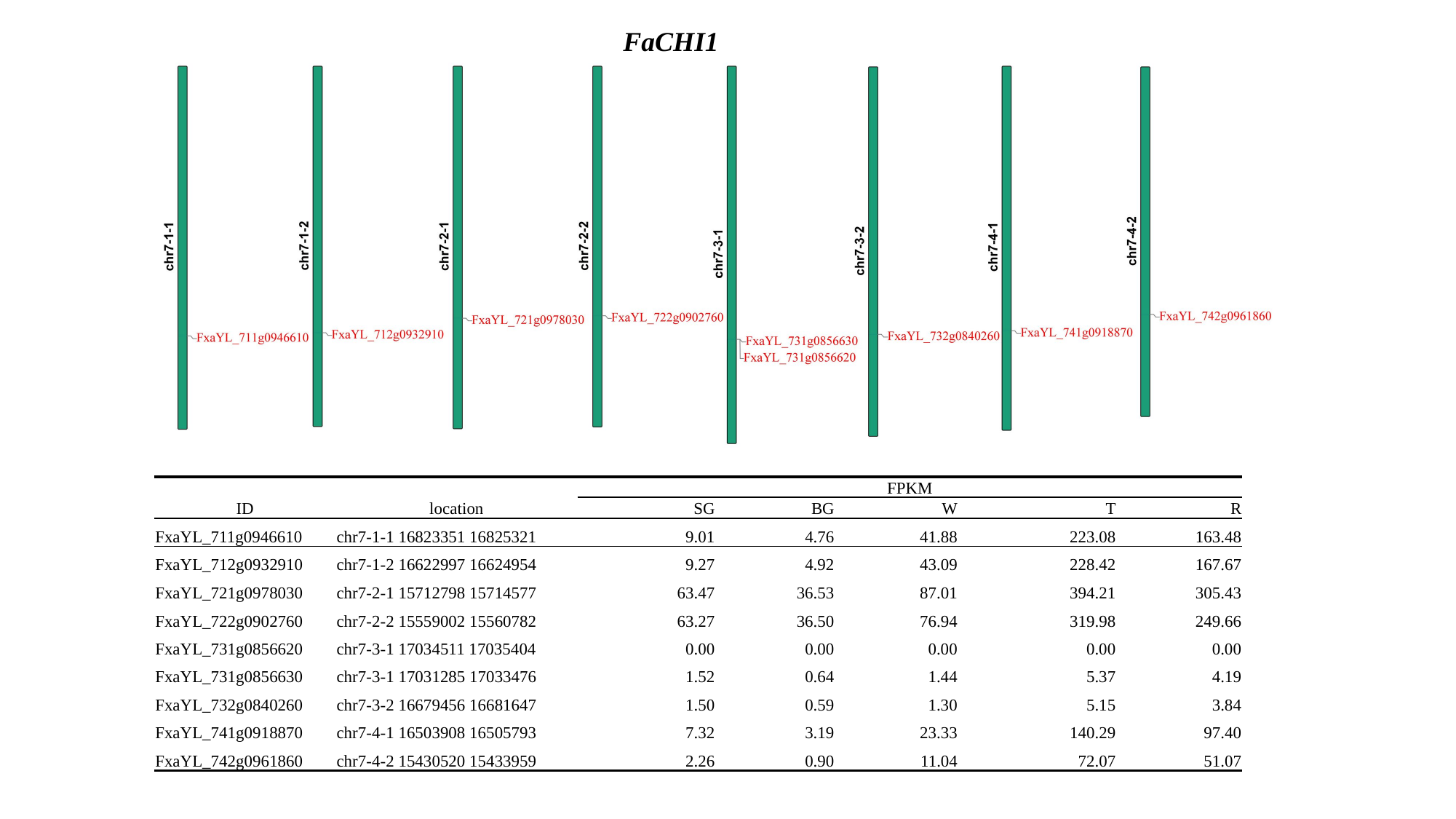

FaCHI1
| | | FPKM | | | | |
| --- | --- | --- | --- | --- | --- | --- |
| ID | location | SG | BG | W | T | R |
| FxaYL\_711g0946610 | chr7-1-1 16823351 16825321 | 9.01 | 4.76 | 41.88 | 223.08 | 163.48 |
| FxaYL\_712g0932910 | chr7-1-2 16622997 16624954 | 9.27 | 4.92 | 43.09 | 228.42 | 167.67 |
| FxaYL\_721g0978030 | chr7-2-1 15712798 15714577 | 63.47 | 36.53 | 87.01 | 394.21 | 305.43 |
| FxaYL\_722g0902760 | chr7-2-2 15559002 15560782 | 63.27 | 36.50 | 76.94 | 319.98 | 249.66 |
| FxaYL\_731g0856620 | chr7-3-1 17034511 17035404 | 0.00 | 0.00 | 0.00 | 0.00 | 0.00 |
| FxaYL\_731g0856630 | chr7-3-1 17031285 17033476 | 1.52 | 0.64 | 1.44 | 5.37 | 4.19 |
| FxaYL\_732g0840260 | chr7-3-2 16679456 16681647 | 1.50 | 0.59 | 1.30 | 5.15 | 3.84 |
| FxaYL\_741g0918870 | chr7-4-1 16503908 16505793 | 7.32 | 3.19 | 23.33 | 140.29 | 97.40 |
| FxaYL\_742g0961860 | chr7-4-2 15430520 15433959 | 2.26 | 0.90 | 11.04 | 72.07 | 51.07 |

## Slide 5
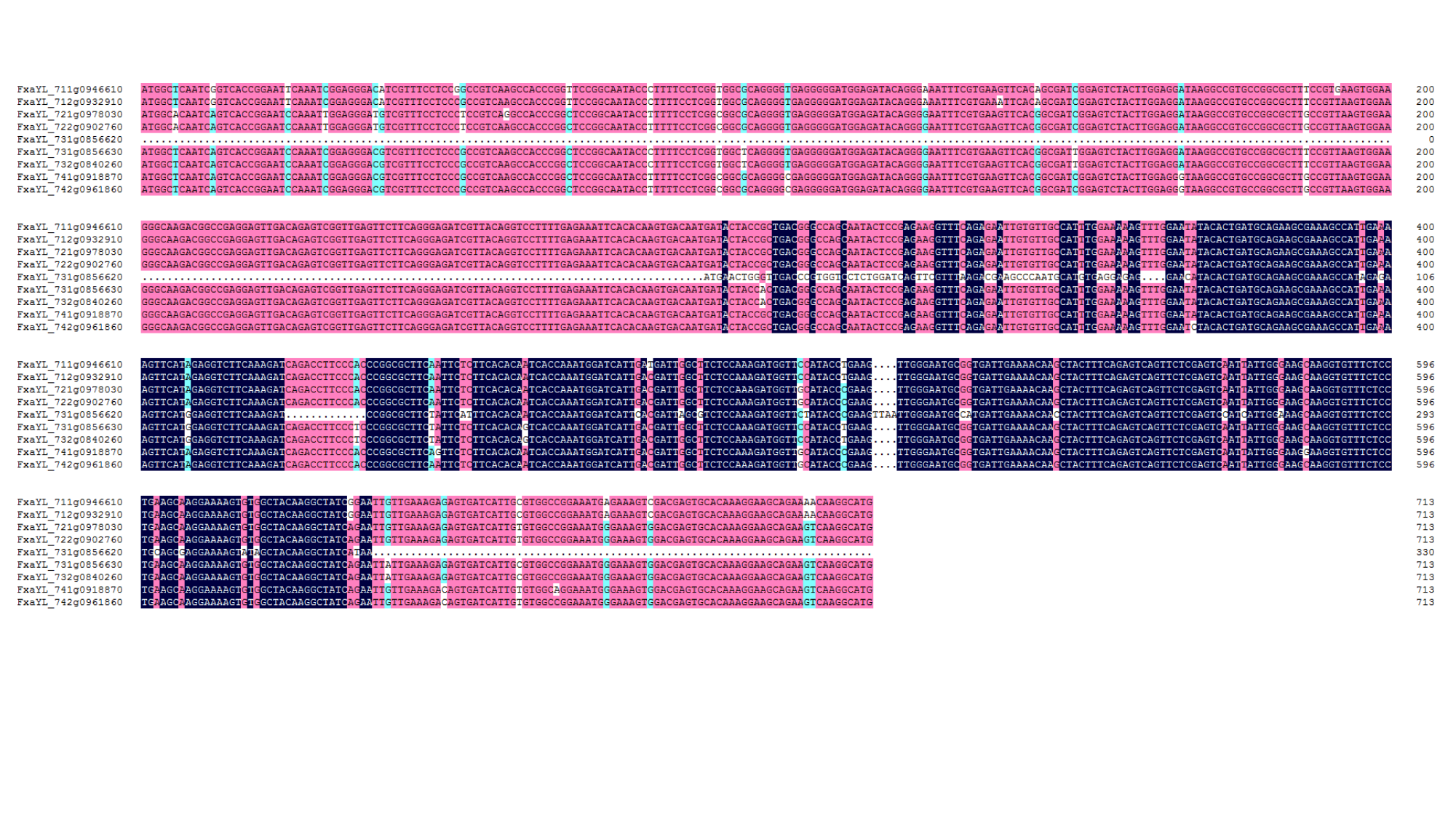

## Slide 6
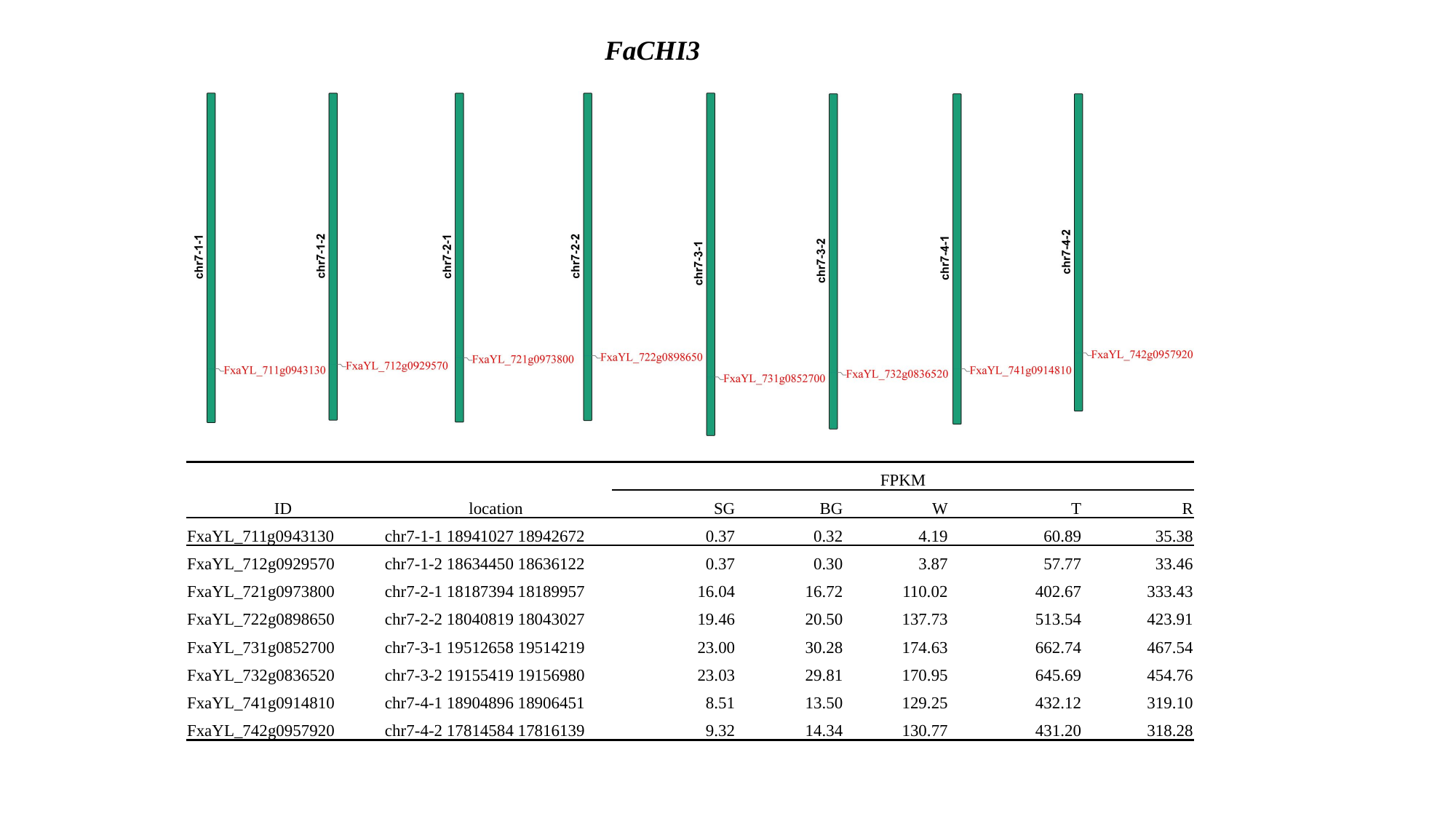

FaCHI3
| | | FPKM | | | | |
| --- | --- | --- | --- | --- | --- | --- |
| ID | location | SG | BG | W | T | R |
| FxaYL\_711g0943130 | chr7-1-1 18941027 18942672 | 0.37 | 0.32 | 4.19 | 60.89 | 35.38 |
| FxaYL\_712g0929570 | chr7-1-2 18634450 18636122 | 0.37 | 0.30 | 3.87 | 57.77 | 33.46 |
| FxaYL\_721g0973800 | chr7-2-1 18187394 18189957 | 16.04 | 16.72 | 110.02 | 402.67 | 333.43 |
| FxaYL\_722g0898650 | chr7-2-2 18040819 18043027 | 19.46 | 20.50 | 137.73 | 513.54 | 423.91 |
| FxaYL\_731g0852700 | chr7-3-1 19512658 19514219 | 23.00 | 30.28 | 174.63 | 662.74 | 467.54 |
| FxaYL\_732g0836520 | chr7-3-2 19155419 19156980 | 23.03 | 29.81 | 170.95 | 645.69 | 454.76 |
| FxaYL\_741g0914810 | chr7-4-1 18904896 18906451 | 8.51 | 13.50 | 129.25 | 432.12 | 319.10 |
| FxaYL\_742g0957920 | chr7-4-2 17814584 17816139 | 9.32 | 14.34 | 130.77 | 431.20 | 318.28 |

## Slide 7
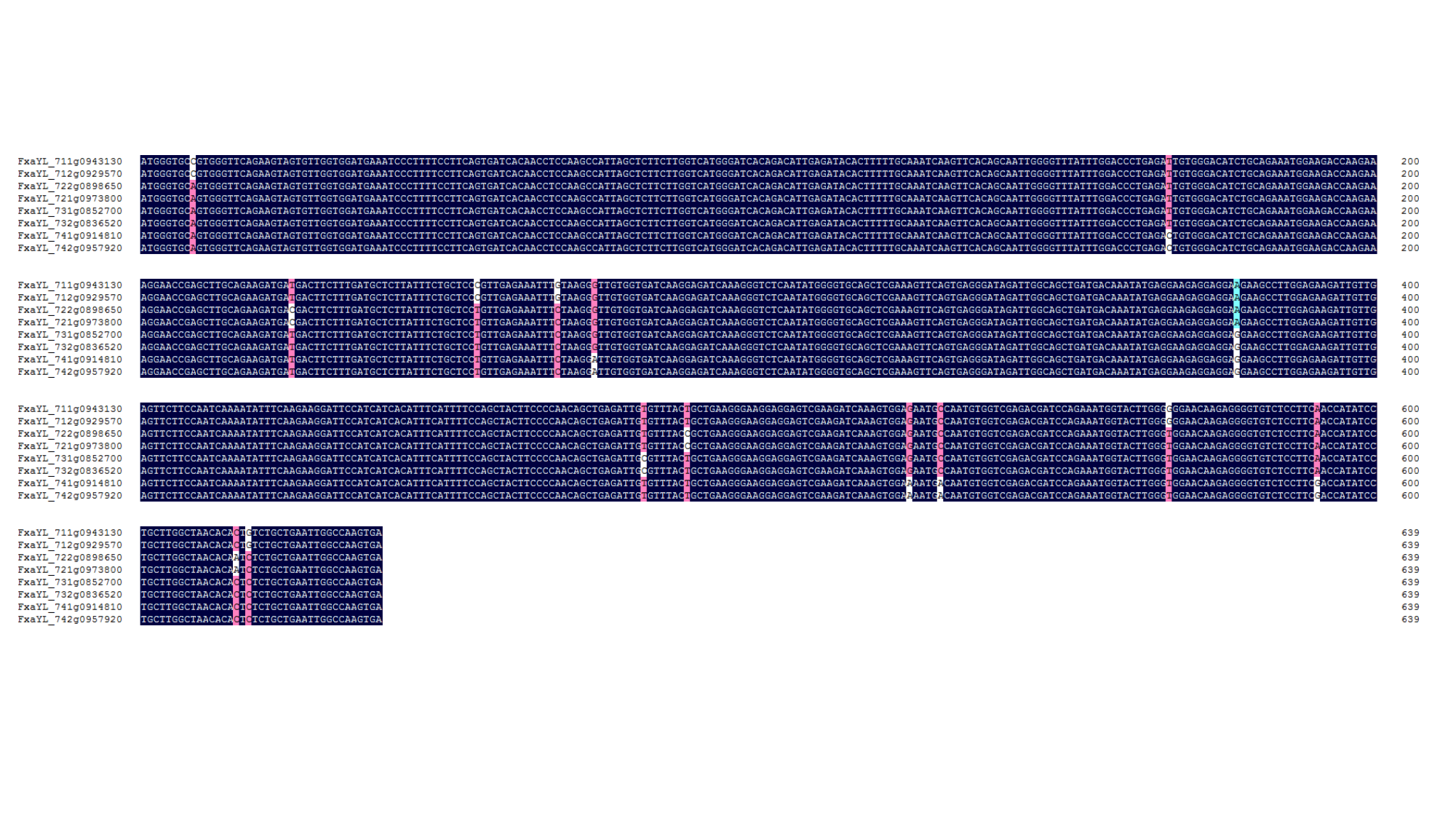

## Slide 8
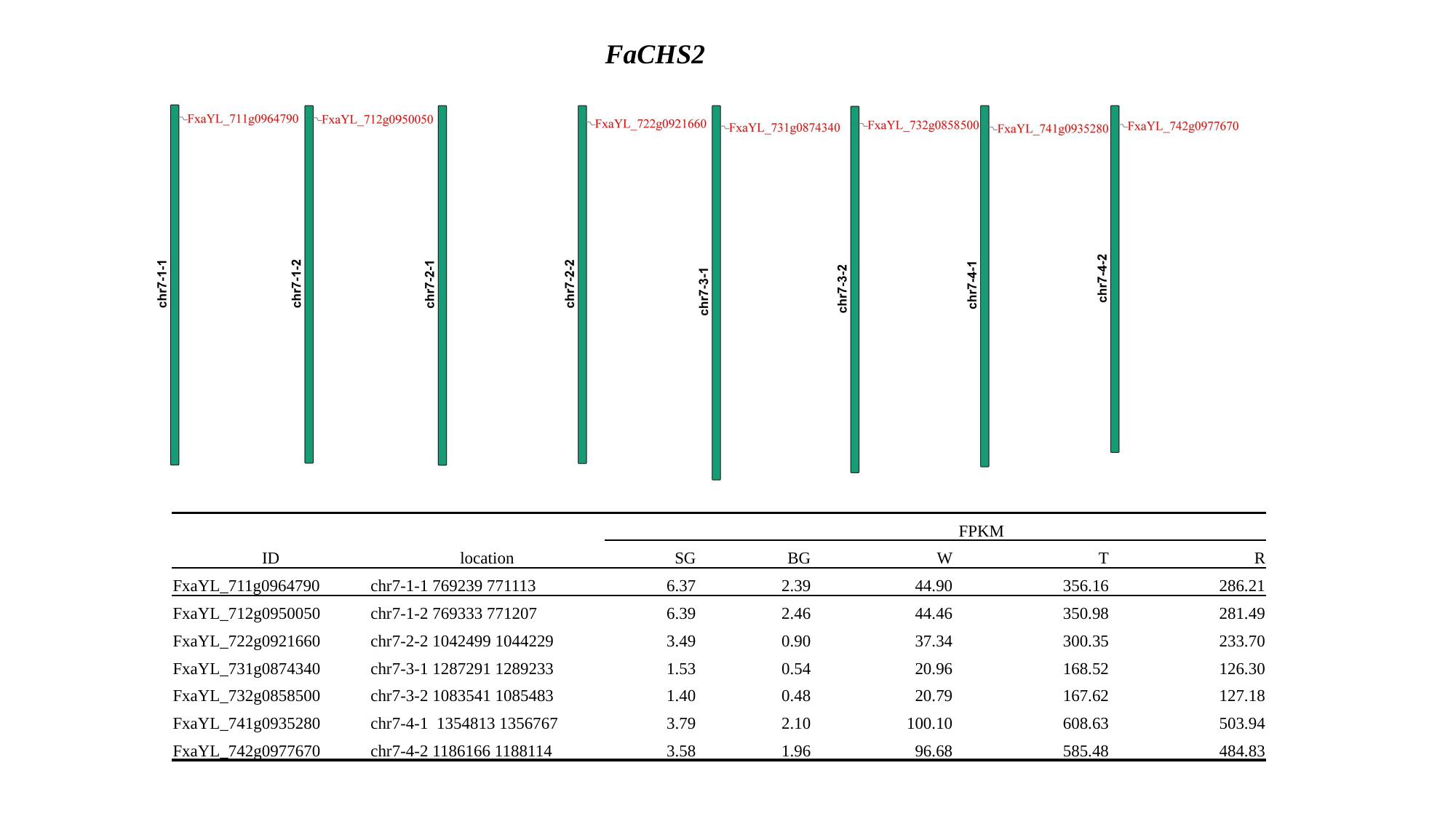

FaCHS2
| | | | FPKM | | | |
| --- | --- | --- | --- | --- | --- | --- |
| ID | location | SG | BG | W | T | R |
| FxaYL\_711g0964790 | chr7-1-1 769239 771113 | 6.37 | 2.39 | 44.90 | 356.16 | 286.21 |
| FxaYL\_712g0950050 | chr7-1-2 769333 771207 | 6.39 | 2.46 | 44.46 | 350.98 | 281.49 |
| FxaYL\_722g0921660 | chr7-2-2 1042499 1044229 | 3.49 | 0.90 | 37.34 | 300.35 | 233.70 |
| FxaYL\_731g0874340 | chr7-3-1 1287291 1289233 | 1.53 | 0.54 | 20.96 | 168.52 | 126.30 |
| FxaYL\_732g0858500 | chr7-3-2 1083541 1085483 | 1.40 | 0.48 | 20.79 | 167.62 | 127.18 |
| FxaYL\_741g0935280 | chr7-4-1 1354813 1356767 | 3.79 | 2.10 | 100.10 | 608.63 | 503.94 |
| FxaYL\_742g0977670 | chr7-4-2 1186166 1188114 | 3.58 | 1.96 | 96.68 | 585.48 | 484.83 |

## Slide 9
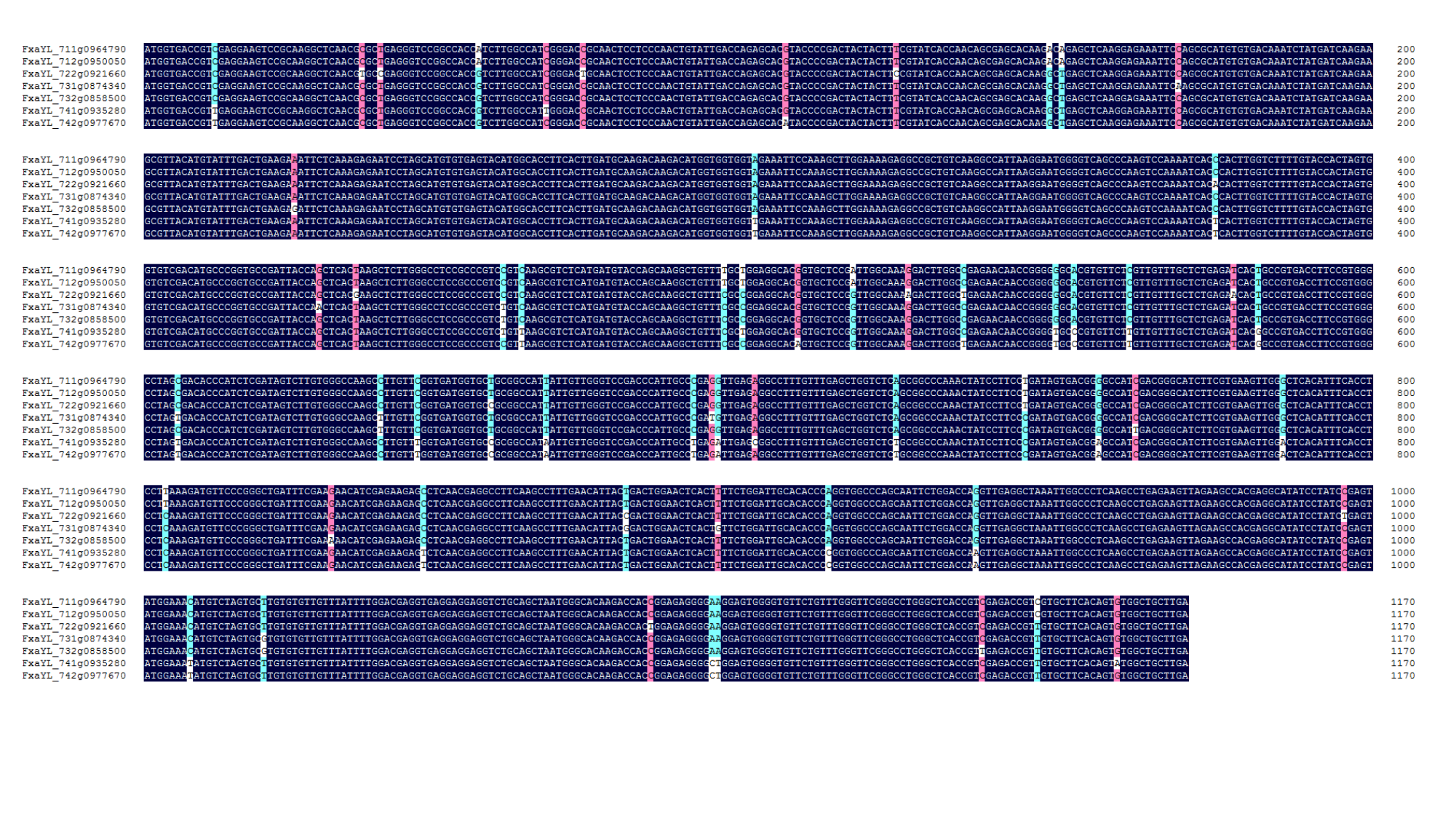

## Slide 10
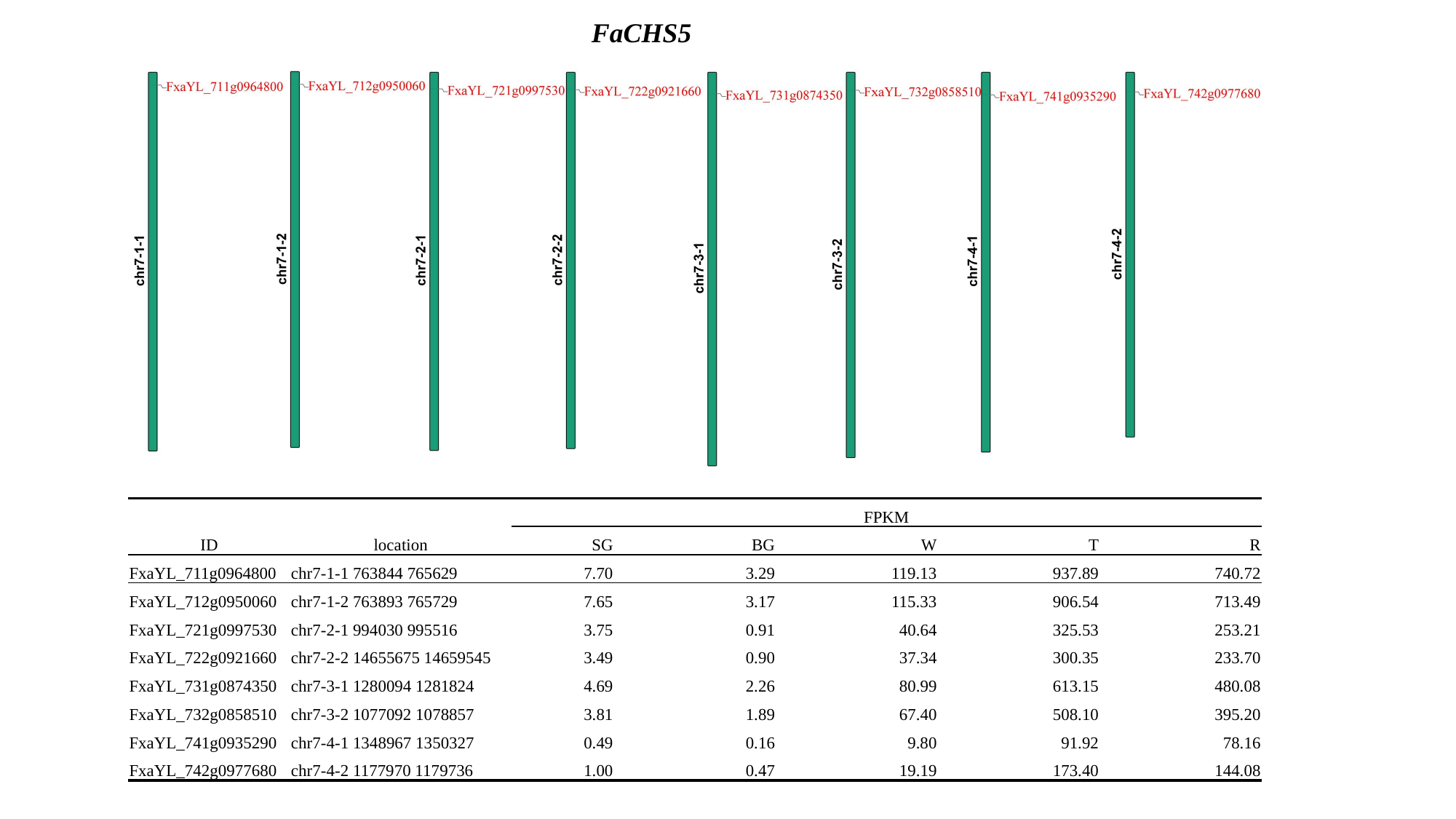

FaCHS5
| | | FPKM | | | | |
| --- | --- | --- | --- | --- | --- | --- |
| ID | location | SG | BG | W | T | R |
| FxaYL\_711g0964800 | chr7-1-1 763844 765629 | 7.70 | 3.29 | 119.13 | 937.89 | 740.72 |
| FxaYL\_712g0950060 | chr7-1-2 763893 765729 | 7.65 | 3.17 | 115.33 | 906.54 | 713.49 |
| FxaYL\_721g0997530 | chr7-2-1 994030 995516 | 3.75 | 0.91 | 40.64 | 325.53 | 253.21 |
| FxaYL\_722g0921660 | chr7-2-2 14655675 14659545 | 3.49 | 0.90 | 37.34 | 300.35 | 233.70 |
| FxaYL\_731g0874350 | chr7-3-1 1280094 1281824 | 4.69 | 2.26 | 80.99 | 613.15 | 480.08 |
| FxaYL\_732g0858510 | chr7-3-2 1077092 1078857 | 3.81 | 1.89 | 67.40 | 508.10 | 395.20 |
| FxaYL\_741g0935290 | chr7-4-1 1348967 1350327 | 0.49 | 0.16 | 9.80 | 91.92 | 78.16 |
| FxaYL\_742g0977680 | chr7-4-2 1177970 1179736 | 1.00 | 0.47 | 19.19 | 173.40 | 144.08 |

## Slide 11
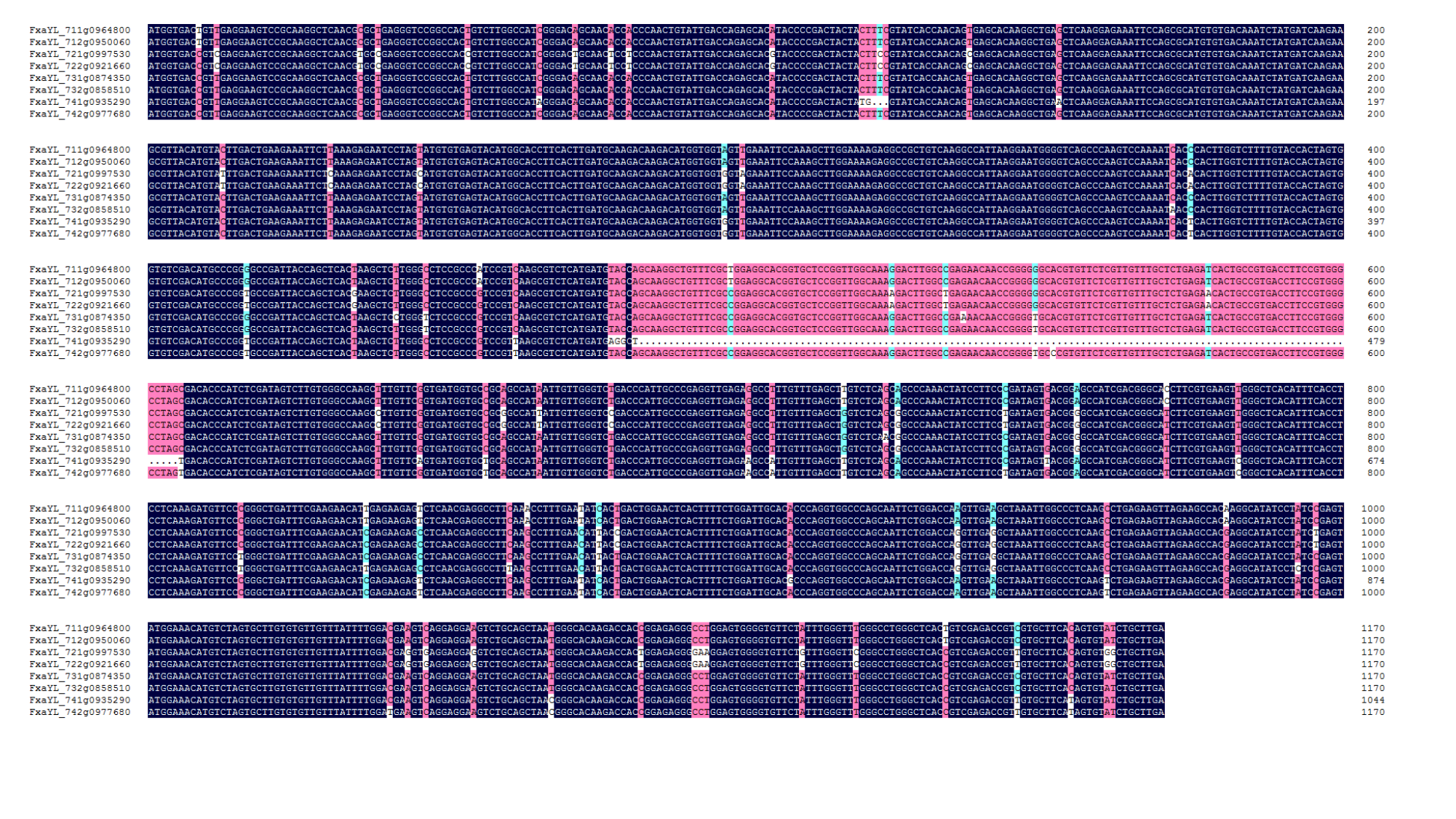

## Slide 12
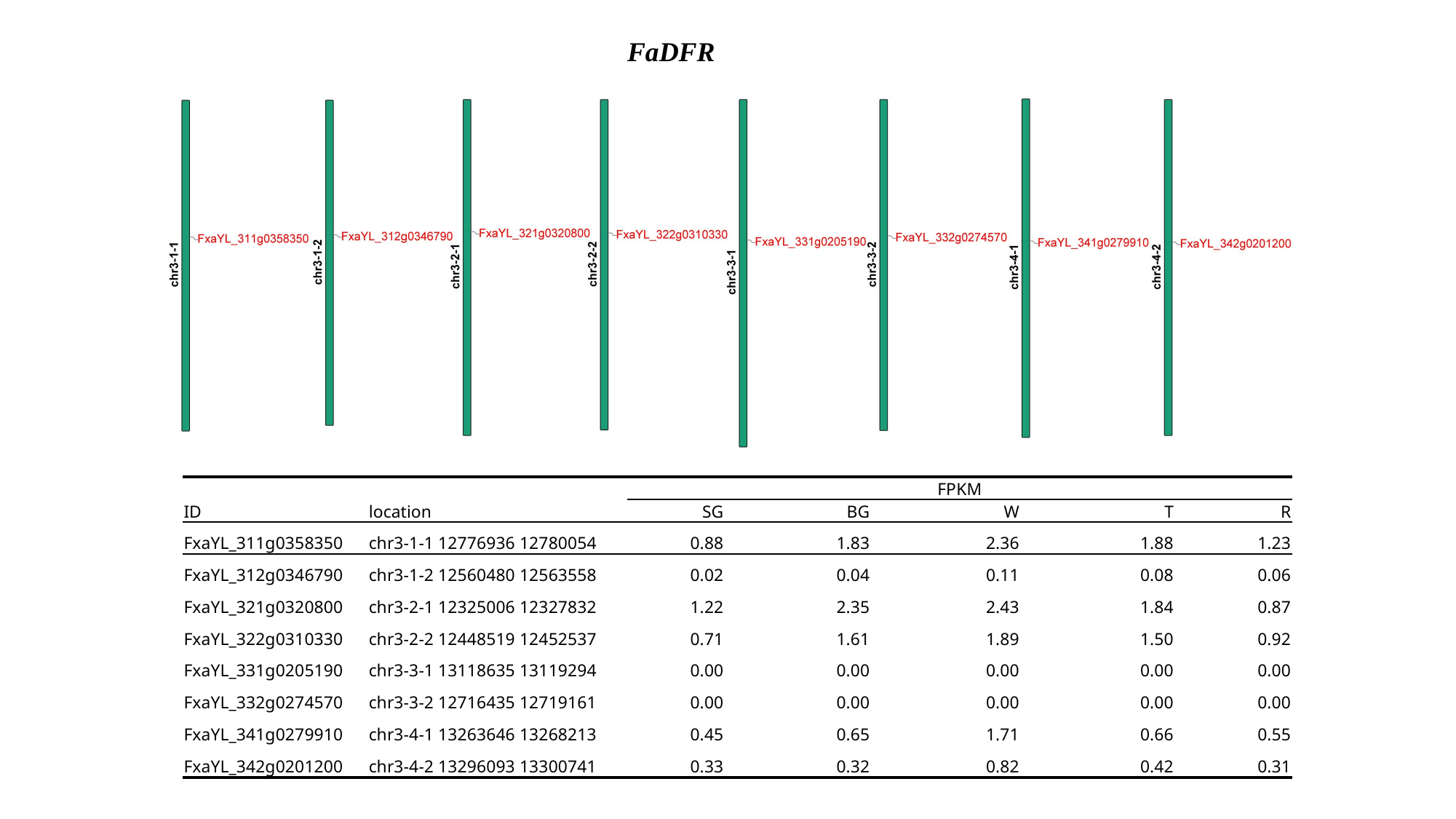

FaDFR
| | | FPKM | | | | |
| --- | --- | --- | --- | --- | --- | --- |
| ID | location | SG | BG | W | T | R |
| FxaYL\_311g0358350 | chr3-1-1 12776936 12780054 | 0.88 | 1.83 | 2.36 | 1.88 | 1.23 |
| FxaYL\_312g0346790 | chr3-1-2 12560480 12563558 | 0.02 | 0.04 | 0.11 | 0.08 | 0.06 |
| FxaYL\_321g0320800 | chr3-2-1 12325006 12327832 | 1.22 | 2.35 | 2.43 | 1.84 | 0.87 |
| FxaYL\_322g0310330 | chr3-2-2 12448519 12452537 | 0.71 | 1.61 | 1.89 | 1.50 | 0.92 |
| FxaYL\_331g0205190 | chr3-3-1 13118635 13119294 | 0.00 | 0.00 | 0.00 | 0.00 | 0.00 |
| FxaYL\_332g0274570 | chr3-3-2 12716435 12719161 | 0.00 | 0.00 | 0.00 | 0.00 | 0.00 |
| FxaYL\_341g0279910 | chr3-4-1 13263646 13268213 | 0.45 | 0.65 | 1.71 | 0.66 | 0.55 |
| FxaYL\_342g0201200 | chr3-4-2 13296093 13300741 | 0.33 | 0.32 | 0.82 | 0.42 | 0.31 |

## Slide 13
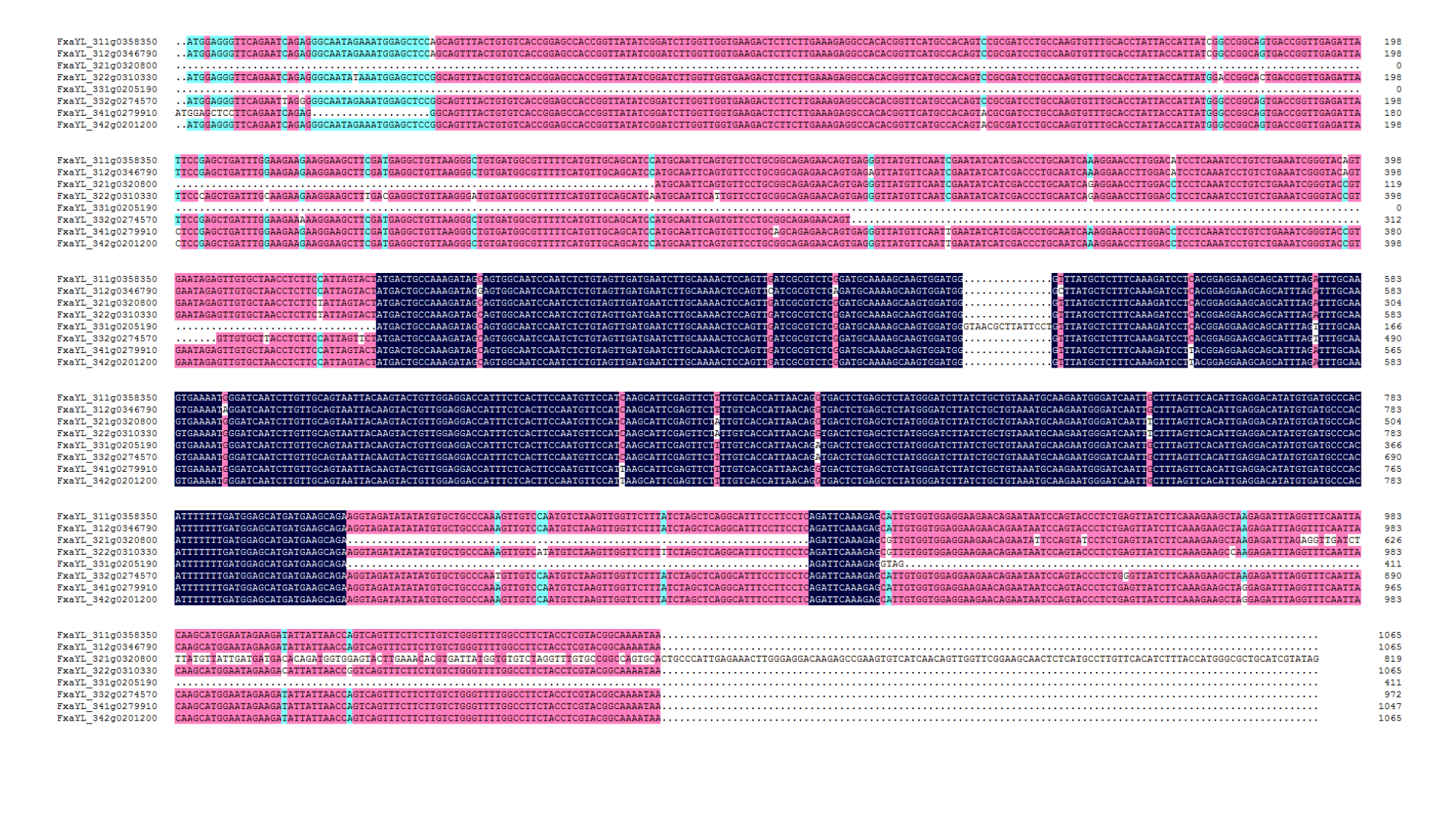

## Slide 14
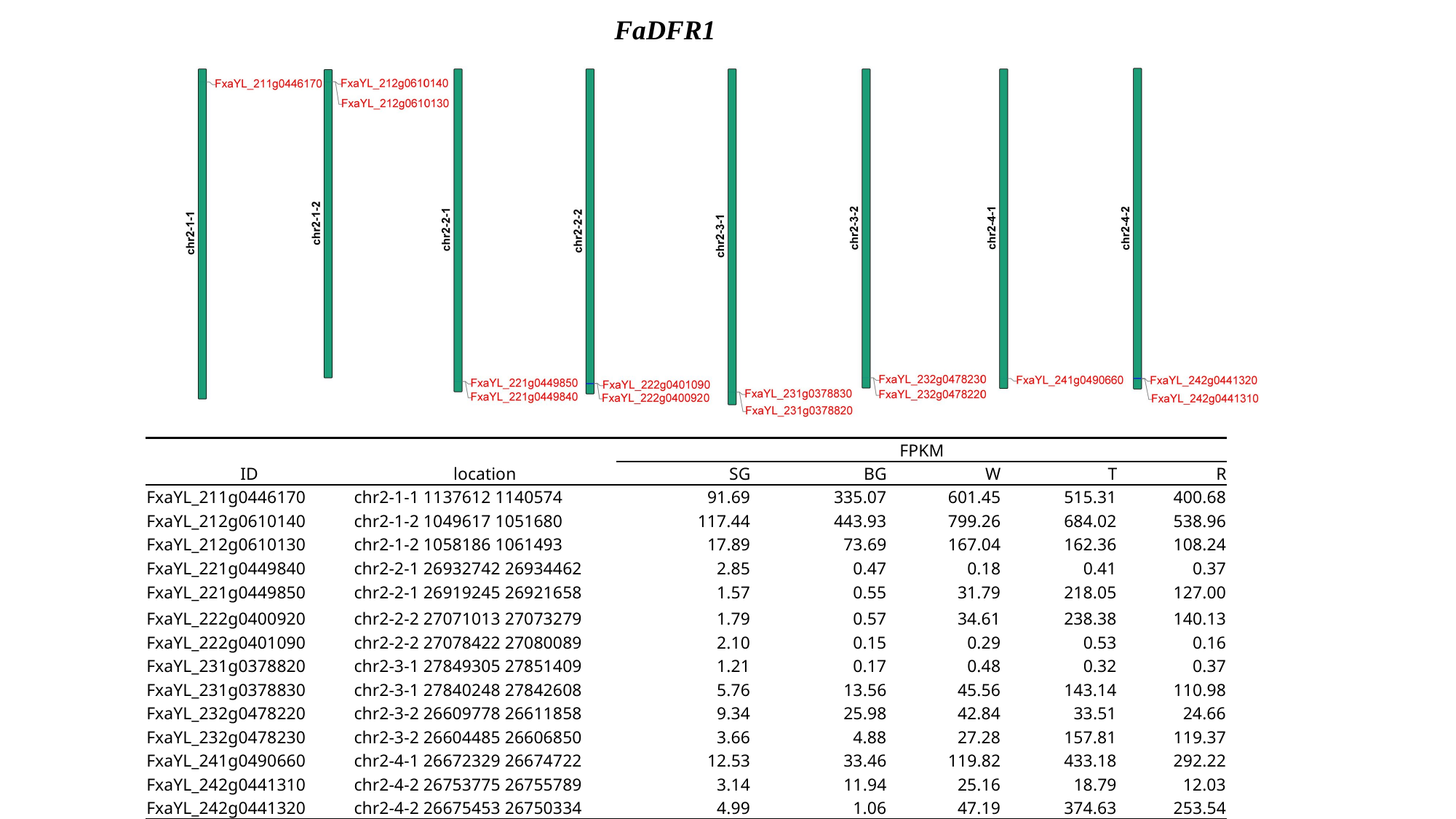

FaDFR1
| | | FPKM | | | | |
| --- | --- | --- | --- | --- | --- | --- |
| ID | location | SG | BG | W | T | R |
| FxaYL\_211g0446170 | chr2-1-1 1137612 1140574 | 91.69 | 335.07 | 601.45 | 515.31 | 400.68 |
| FxaYL\_212g0610140 | chr2-1-2 1049617 1051680 | 117.44 | 443.93 | 799.26 | 684.02 | 538.96 |
| FxaYL\_212g0610130 | chr2-1-2 1058186 1061493 | 17.89 | 73.69 | 167.04 | 162.36 | 108.24 |
| FxaYL\_221g0449840 | chr2-2-1 26932742 26934462 | 2.85 | 0.47 | 0.18 | 0.41 | 0.37 |
| FxaYL\_221g0449850 | chr2-2-1 26919245 26921658 | 1.57 | 0.55 | 31.79 | 218.05 | 127.00 |
| FxaYL\_222g0400920 | chr2-2-2 27071013 27073279 | 1.79 | 0.57 | 34.61 | 238.38 | 140.13 |
| FxaYL\_222g0401090 | chr2-2-2 27078422 27080089 | 2.10 | 0.15 | 0.29 | 0.53 | 0.16 |
| FxaYL\_231g0378820 | chr2-3-1 27849305 27851409 | 1.21 | 0.17 | 0.48 | 0.32 | 0.37 |
| FxaYL\_231g0378830 | chr2-3-1 27840248 27842608 | 5.76 | 13.56 | 45.56 | 143.14 | 110.98 |
| FxaYL\_232g0478220 | chr2-3-2 26609778 26611858 | 9.34 | 25.98 | 42.84 | 33.51 | 24.66 |
| FxaYL\_232g0478230 | chr2-3-2 26604485 26606850 | 3.66 | 4.88 | 27.28 | 157.81 | 119.37 |
| FxaYL\_241g0490660 | chr2-4-1 26672329 26674722 | 12.53 | 33.46 | 119.82 | 433.18 | 292.22 |
| FxaYL\_242g0441310 | chr2-4-2 26753775 26755789 | 3.14 | 11.94 | 25.16 | 18.79 | 12.03 |
| FxaYL\_242g0441320 | chr2-4-2 26675453 26750334 | 4.99 | 1.06 | 47.19 | 374.63 | 253.54 |

## Slide 15
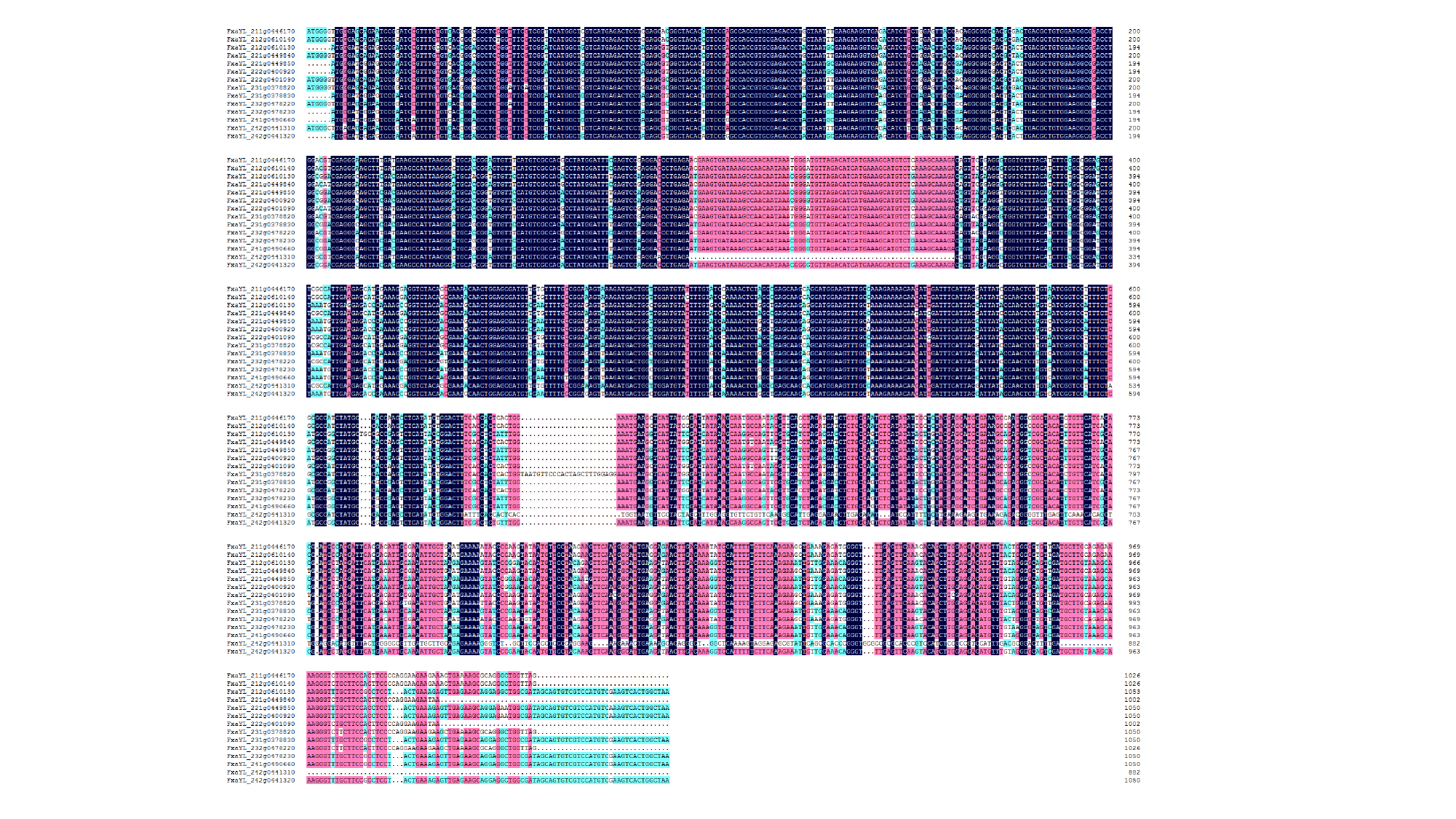

## Slide 16
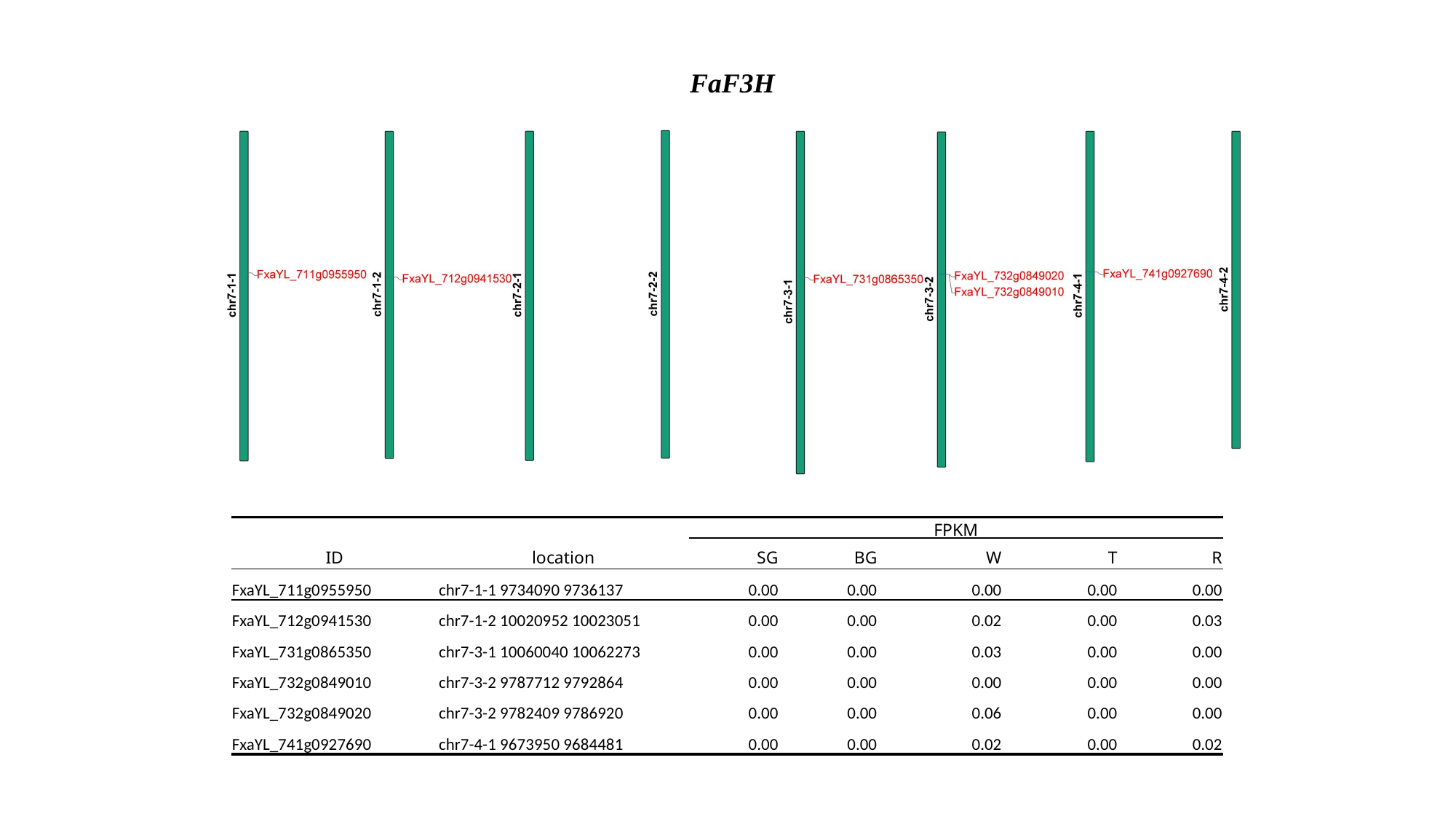

FaF3H
| | | FPKM | | | | |
| --- | --- | --- | --- | --- | --- | --- |
| ID | location | SG | BG | W | T | R |
| FxaYL\_711g0955950 | chr7-1-1 9734090 9736137 | 0.00 | 0.00 | 0.00 | 0.00 | 0.00 |
| FxaYL\_712g0941530 | chr7-1-2 10020952 10023051 | 0.00 | 0.00 | 0.02 | 0.00 | 0.03 |
| FxaYL\_731g0865350 | chr7-3-1 10060040 10062273 | 0.00 | 0.00 | 0.03 | 0.00 | 0.00 |
| FxaYL\_732g0849010 | chr7-3-2 9787712 9792864 | 0.00 | 0.00 | 0.00 | 0.00 | 0.00 |
| FxaYL\_732g0849020 | chr7-3-2 9782409 9786920 | 0.00 | 0.00 | 0.06 | 0.00 | 0.00 |
| FxaYL\_741g0927690 | chr7-4-1 9673950 9684481 | 0.00 | 0.00 | 0.02 | 0.00 | 0.02 |

## Slide 17
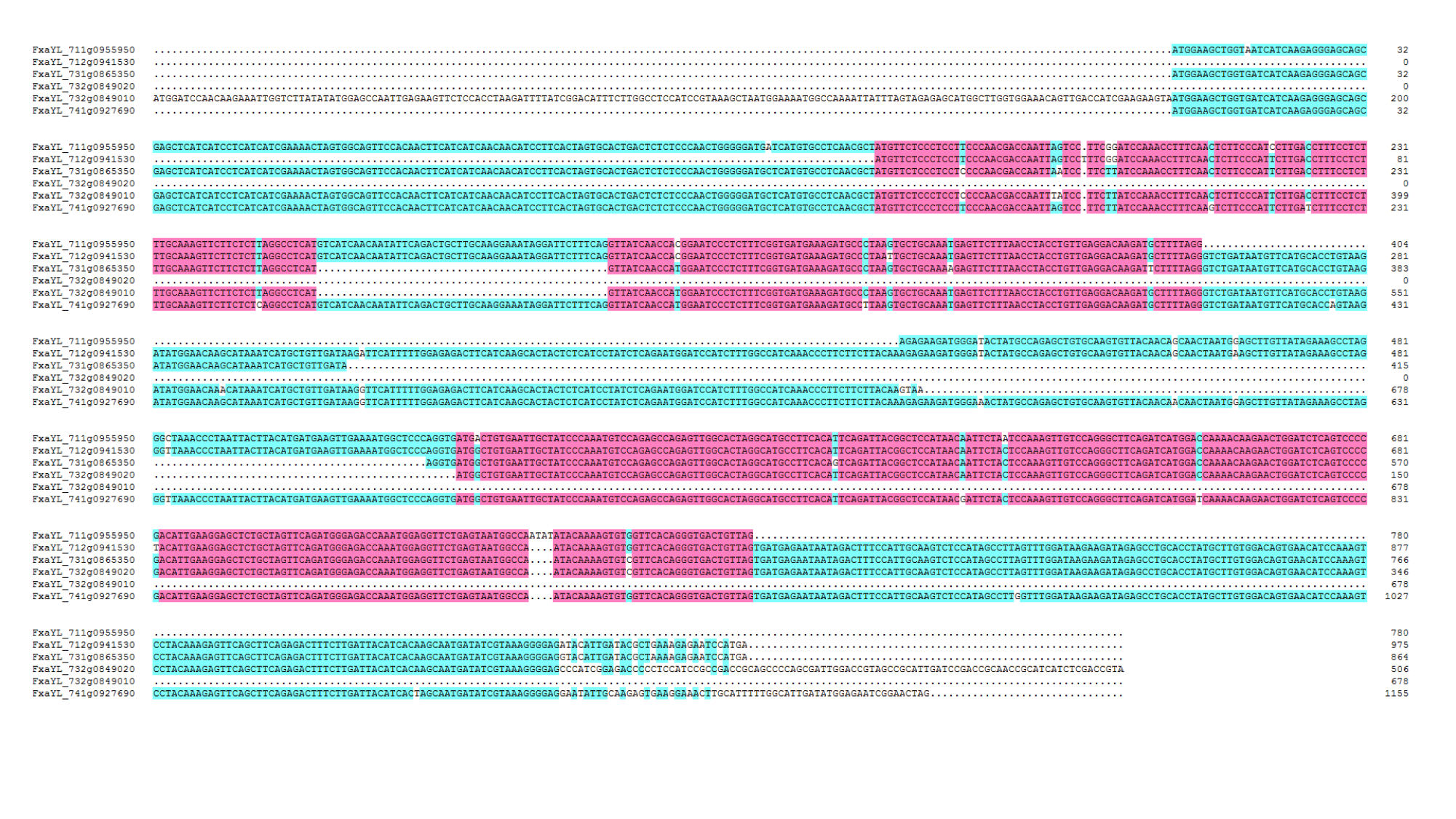

## Slide 18
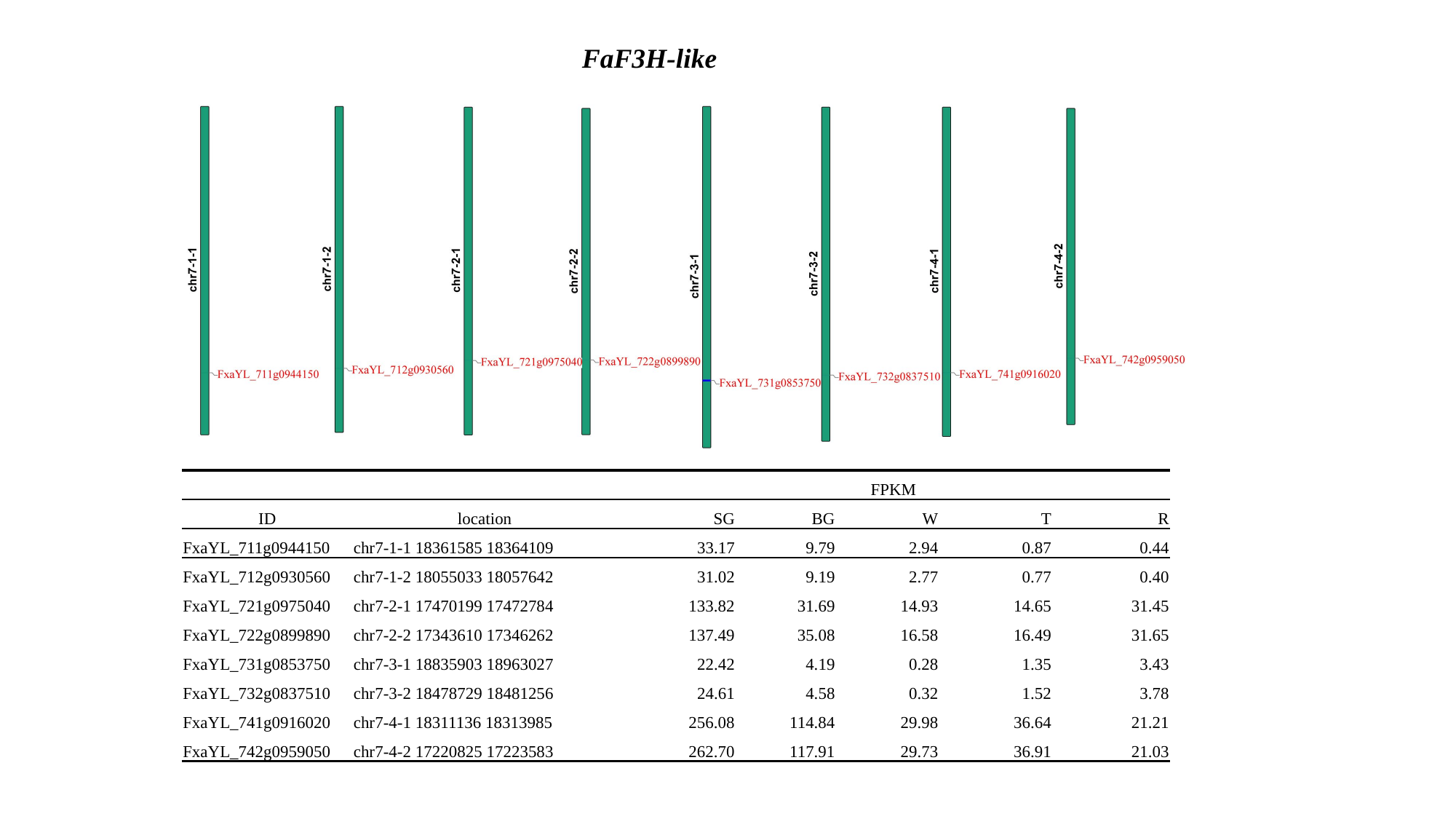

FaF3H-like
| | | FPKM | | | | |
| --- | --- | --- | --- | --- | --- | --- |
| ID | location | SG | BG | W | T | R |
| FxaYL\_711g0944150 | chr7-1-1 18361585 18364109 | 33.17 | 9.79 | 2.94 | 0.87 | 0.44 |
| FxaYL\_712g0930560 | chr7-1-2 18055033 18057642 | 31.02 | 9.19 | 2.77 | 0.77 | 0.40 |
| FxaYL\_721g0975040 | chr7-2-1 17470199 17472784 | 133.82 | 31.69 | 14.93 | 14.65 | 31.45 |
| FxaYL\_722g0899890 | chr7-2-2 17343610 17346262 | 137.49 | 35.08 | 16.58 | 16.49 | 31.65 |
| FxaYL\_731g0853750 | chr7-3-1 18835903 18963027 | 22.42 | 4.19 | 0.28 | 1.35 | 3.43 |
| FxaYL\_732g0837510 | chr7-3-2 18478729 18481256 | 24.61 | 4.58 | 0.32 | 1.52 | 3.78 |
| FxaYL\_741g0916020 | chr7-4-1 18311136 18313985 | 256.08 | 114.84 | 29.98 | 36.64 | 21.21 |
| FxaYL\_742g0959050 | chr7-4-2 17220825 17223583 | 262.70 | 117.91 | 29.73 | 36.91 | 21.03 |

## Slide 19
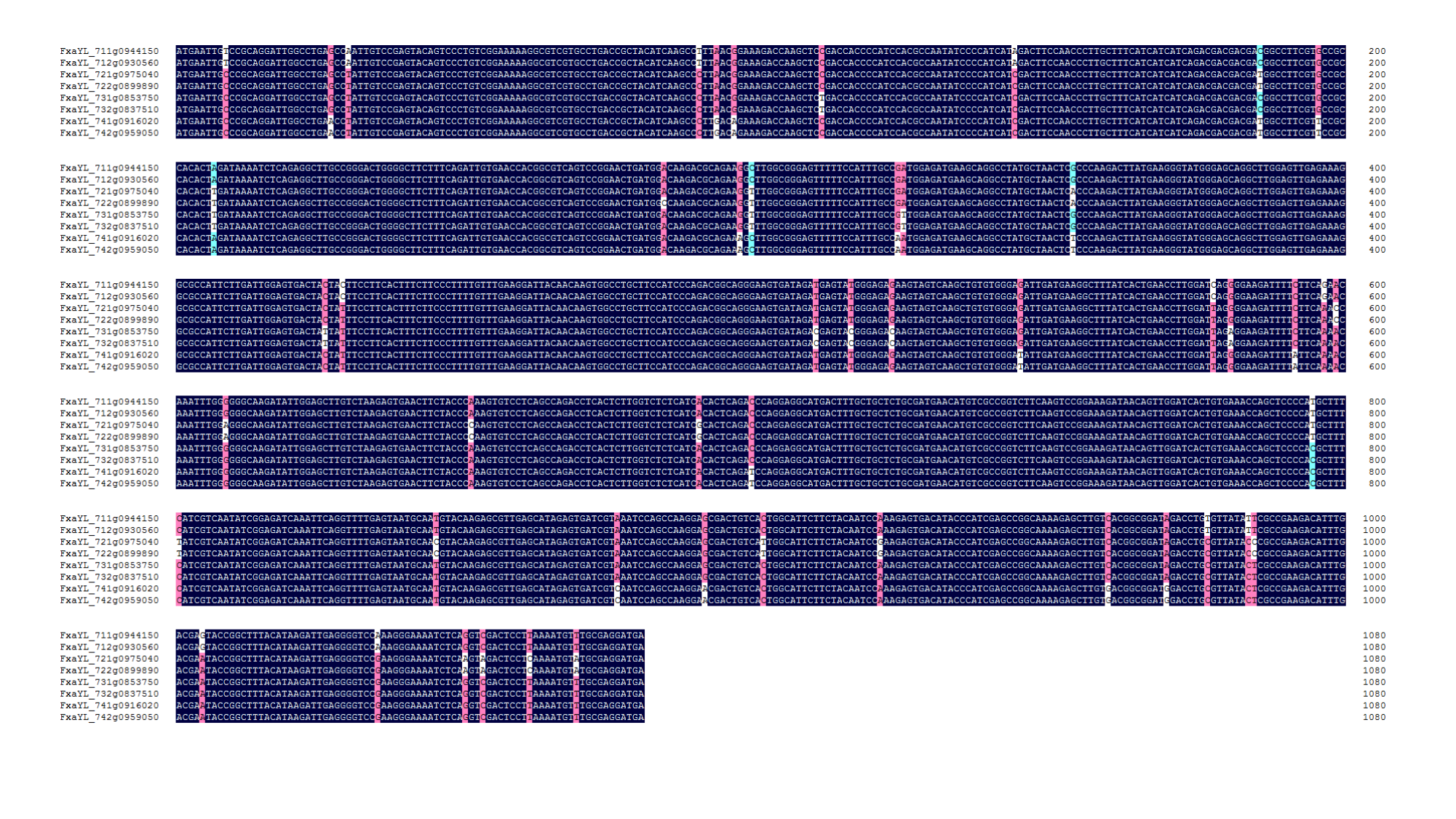

## Slide 20
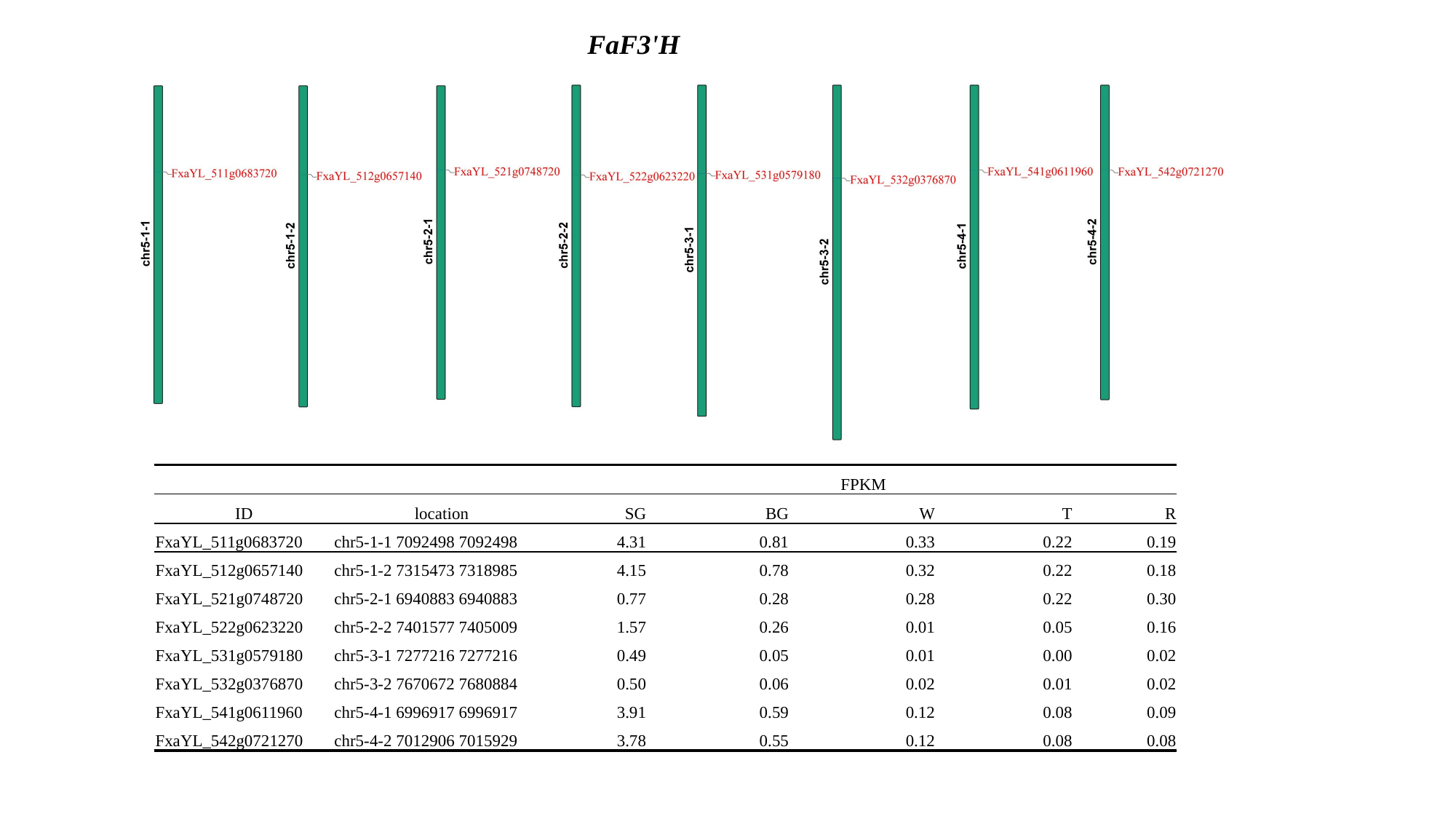

FaF3'H
| | | FPKM | | | | |
| --- | --- | --- | --- | --- | --- | --- |
| ID | location | SG | BG | W | T | R |
| FxaYL\_511g0683720 | chr5-1-1 7092498 7092498 | 4.31 | 0.81 | 0.33 | 0.22 | 0.19 |
| FxaYL\_512g0657140 | chr5-1-2 7315473 7318985 | 4.15 | 0.78 | 0.32 | 0.22 | 0.18 |
| FxaYL\_521g0748720 | chr5-2-1 6940883 6940883 | 0.77 | 0.28 | 0.28 | 0.22 | 0.30 |
| FxaYL\_522g0623220 | chr5-2-2 7401577 7405009 | 1.57 | 0.26 | 0.01 | 0.05 | 0.16 |
| FxaYL\_531g0579180 | chr5-3-1 7277216 7277216 | 0.49 | 0.05 | 0.01 | 0.00 | 0.02 |
| FxaYL\_532g0376870 | chr5-3-2 7670672 7680884 | 0.50 | 0.06 | 0.02 | 0.01 | 0.02 |
| FxaYL\_541g0611960 | chr5-4-1 6996917 6996917 | 3.91 | 0.59 | 0.12 | 0.08 | 0.09 |
| FxaYL\_542g0721270 | chr5-4-2 7012906 7015929 | 3.78 | 0.55 | 0.12 | 0.08 | 0.08 |

## Slide 21
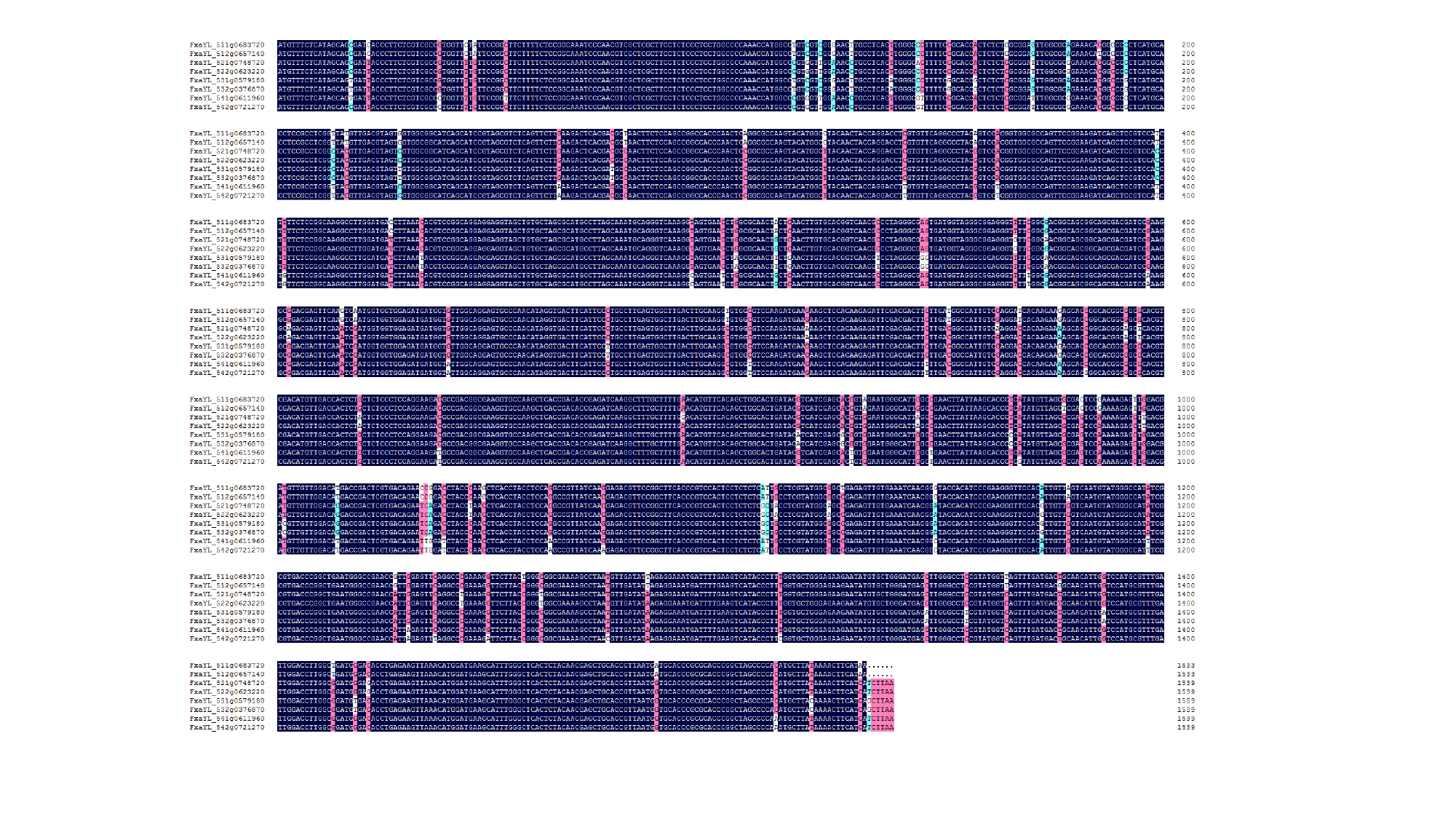

## Slide 22
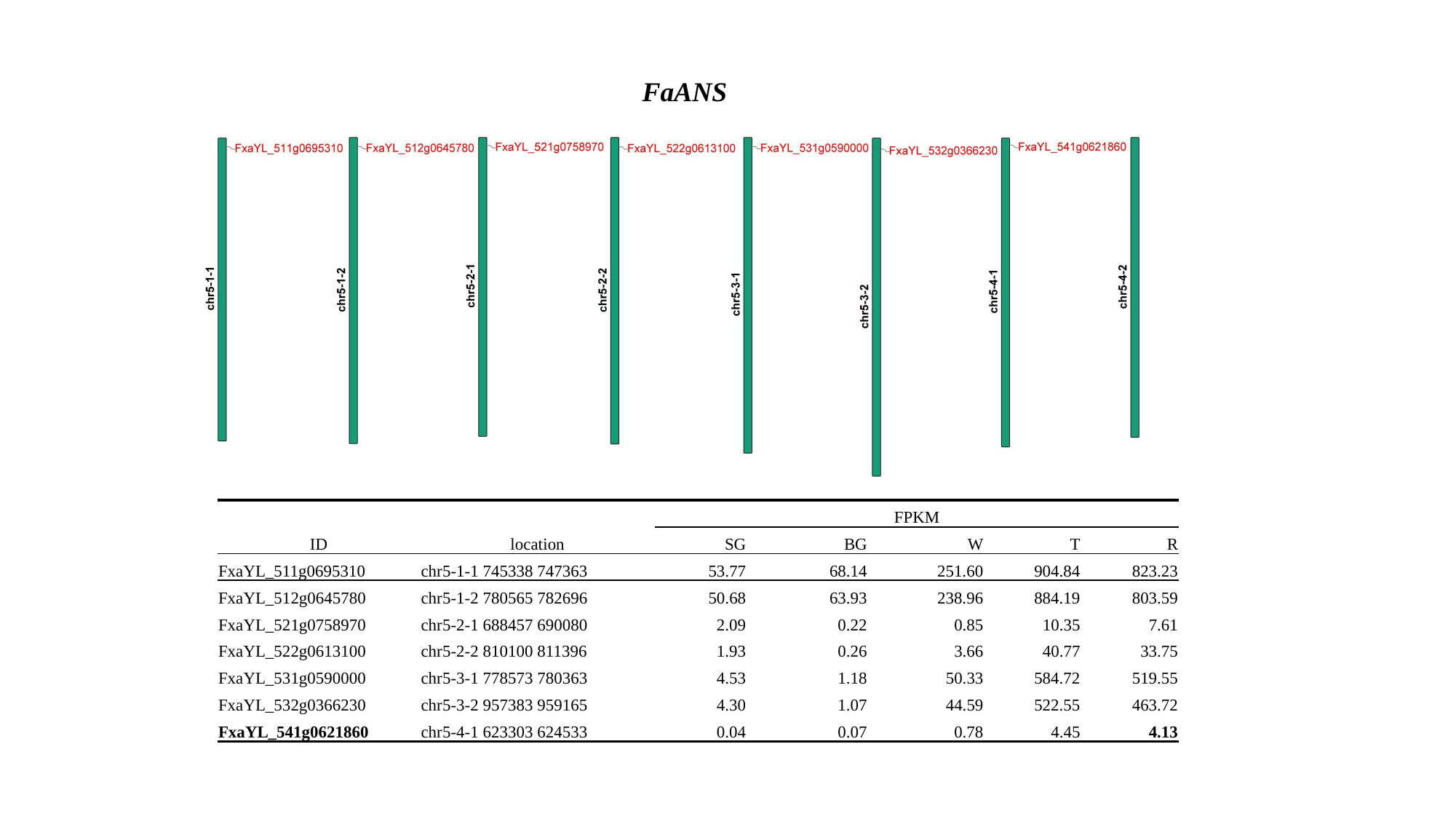

FaANS
| | | FPKM | | | | |
| --- | --- | --- | --- | --- | --- | --- |
| ID | location | SG | BG | W | T | R |
| FxaYL\_511g0695310 | chr5-1-1 745338 747363 | 53.77 | 68.14 | 251.60 | 904.84 | 823.23 |
| FxaYL\_512g0645780 | chr5-1-2 780565 782696 | 50.68 | 63.93 | 238.96 | 884.19 | 803.59 |
| FxaYL\_521g0758970 | chr5-2-1 688457 690080 | 2.09 | 0.22 | 0.85 | 10.35 | 7.61 |
| FxaYL\_522g0613100 | chr5-2-2 810100 811396 | 1.93 | 0.26 | 3.66 | 40.77 | 33.75 |
| FxaYL\_531g0590000 | chr5-3-1 778573 780363 | 4.53 | 1.18 | 50.33 | 584.72 | 519.55 |
| FxaYL\_532g0366230 | chr5-3-2 957383 959165 | 4.30 | 1.07 | 44.59 | 522.55 | 463.72 |
| FxaYL\_541g0621860 | chr5-4-1 623303 624533 | 0.04 | 0.07 | 0.78 | 4.45 | 4.13 |

## Slide 23
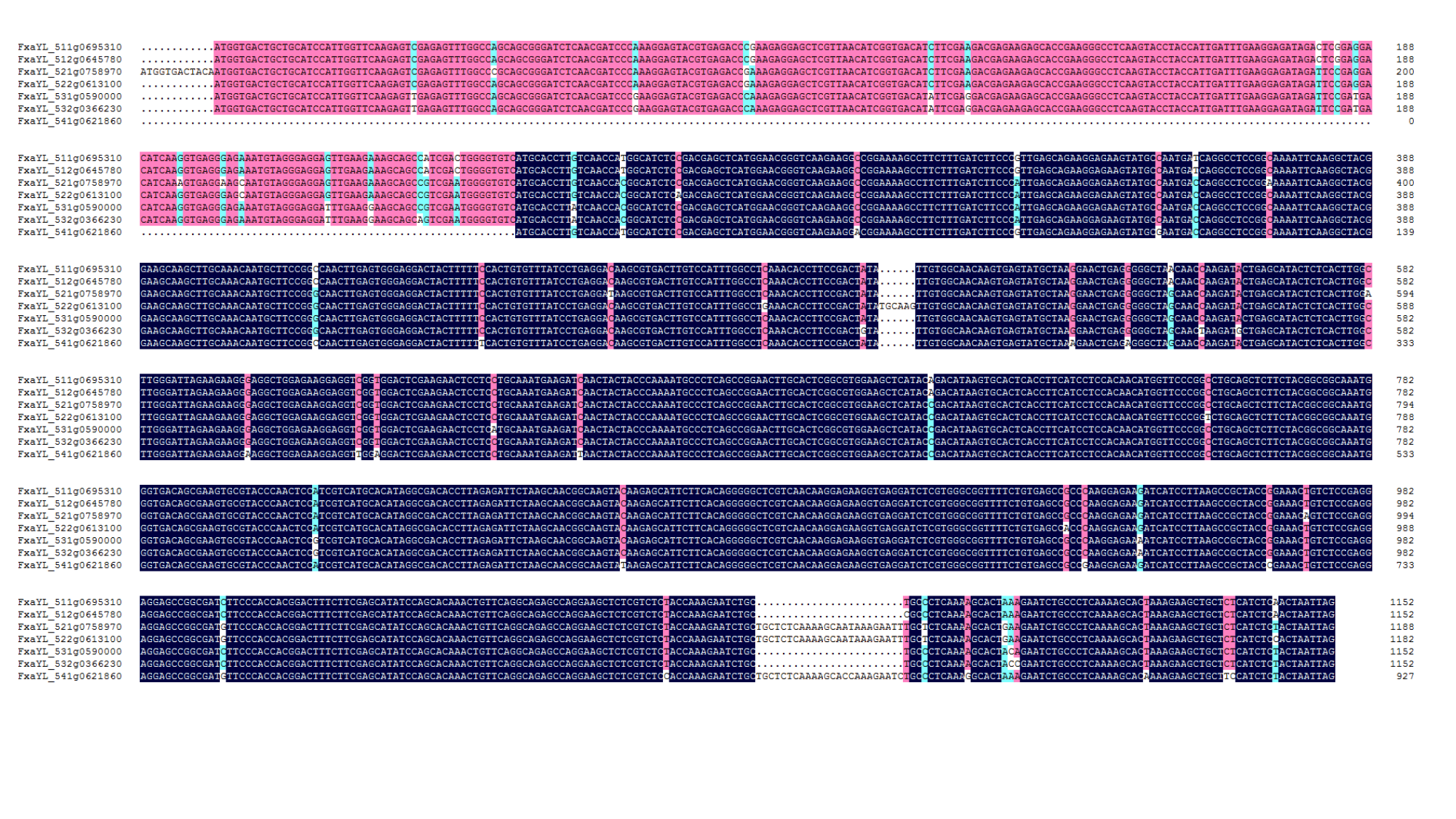

## Slide 24
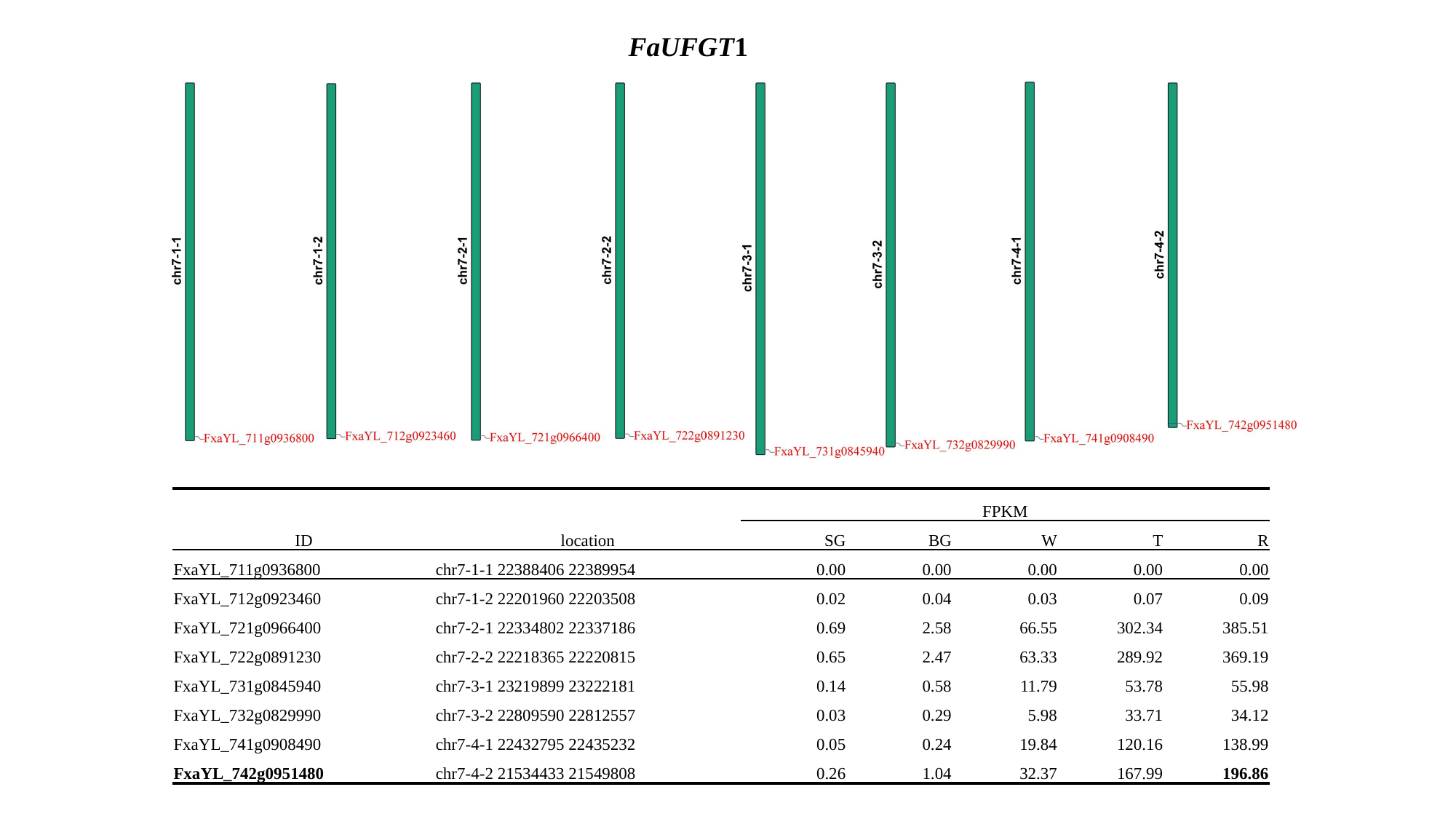

FaUFGT1
| | | FPKM | | | | |
| --- | --- | --- | --- | --- | --- | --- |
| ID | location | SG | BG | W | T | R |
| FxaYL\_711g0936800 | chr7-1-1 22388406 22389954 | 0.00 | 0.00 | 0.00 | 0.00 | 0.00 |
| FxaYL\_712g0923460 | chr7-1-2 22201960 22203508 | 0.02 | 0.04 | 0.03 | 0.07 | 0.09 |
| FxaYL\_721g0966400 | chr7-2-1 22334802 22337186 | 0.69 | 2.58 | 66.55 | 302.34 | 385.51 |
| FxaYL\_722g0891230 | chr7-2-2 22218365 22220815 | 0.65 | 2.47 | 63.33 | 289.92 | 369.19 |
| FxaYL\_731g0845940 | chr7-3-1 23219899 23222181 | 0.14 | 0.58 | 11.79 | 53.78 | 55.98 |
| FxaYL\_732g0829990 | chr7-3-2 22809590 22812557 | 0.03 | 0.29 | 5.98 | 33.71 | 34.12 |
| FxaYL\_741g0908490 | chr7-4-1 22432795 22435232 | 0.05 | 0.24 | 19.84 | 120.16 | 138.99 |
| FxaYL\_742g0951480 | chr7-4-2 21534433 21549808 | 0.26 | 1.04 | 32.37 | 167.99 | 196.86 |

## Slide 25
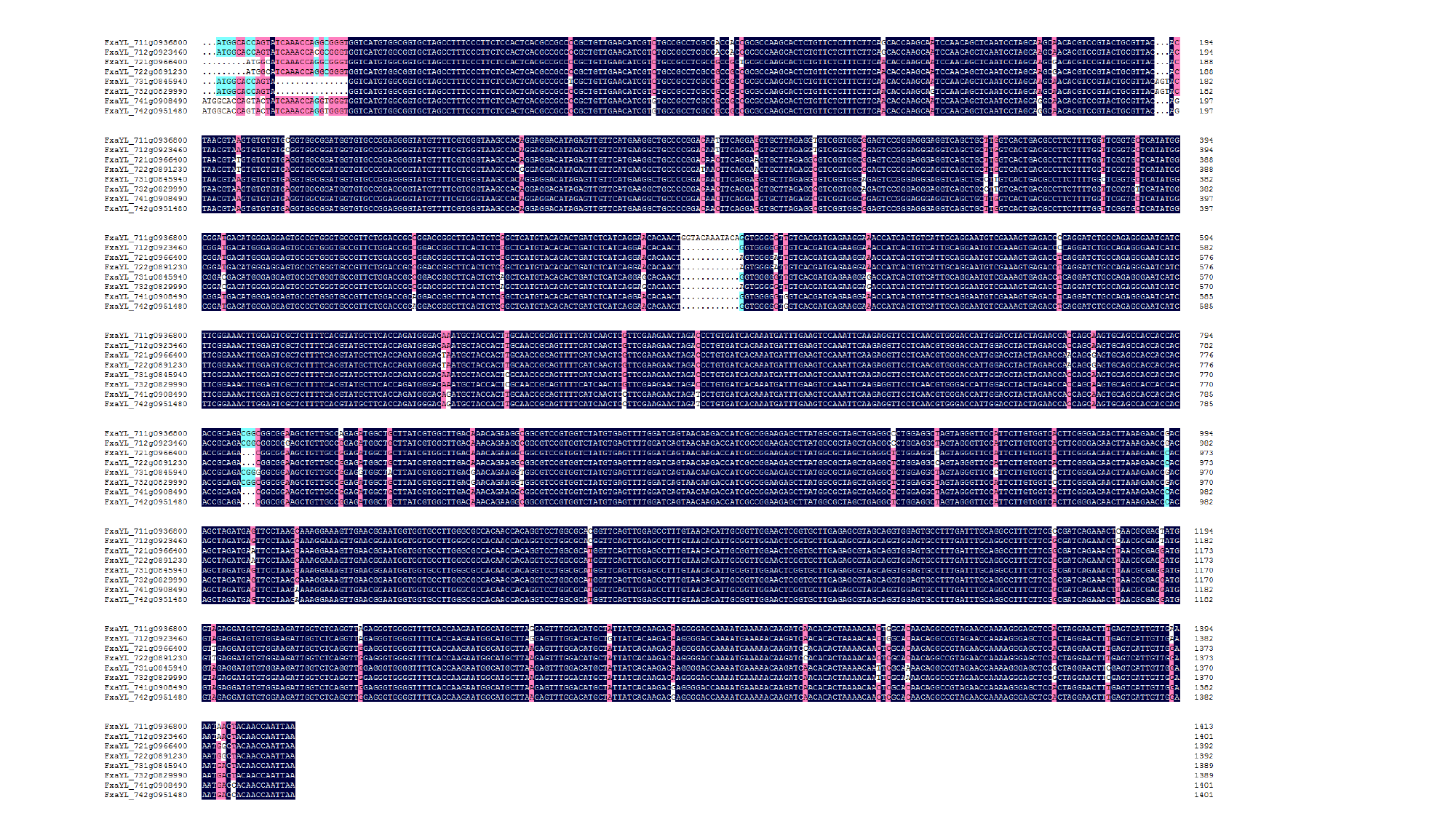

## Slide 26
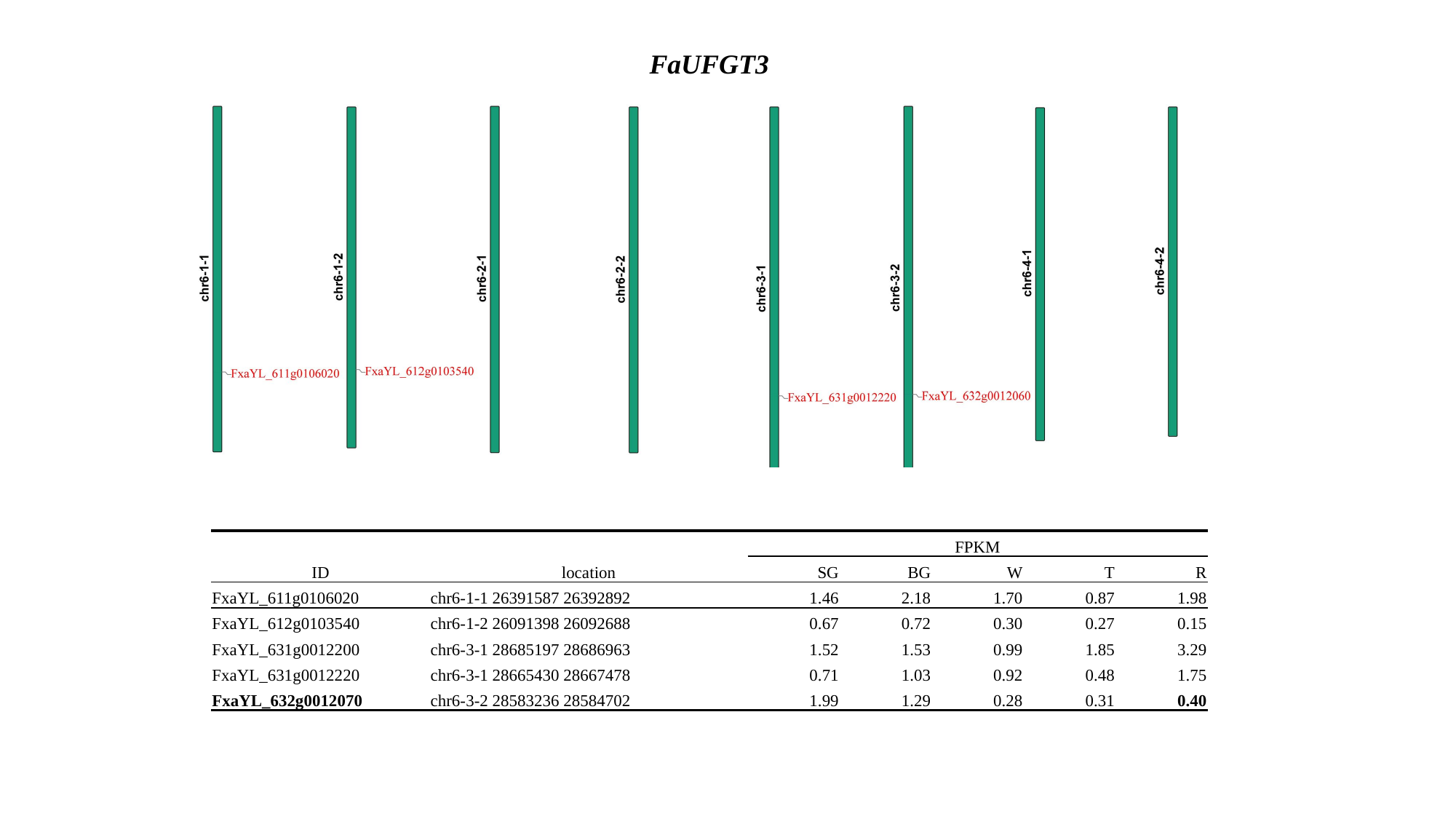

FaUFGT3
| | | FPKM | | | | |
| --- | --- | --- | --- | --- | --- | --- |
| ID | location | SG | BG | W | T | R |
| FxaYL\_611g0106020 | chr6-1-1 26391587 26392892 | 1.46 | 2.18 | 1.70 | 0.87 | 1.98 |
| FxaYL\_612g0103540 | chr6-1-2 26091398 26092688 | 0.67 | 0.72 | 0.30 | 0.27 | 0.15 |
| FxaYL\_631g0012200 | chr6-3-1 28685197 28686963 | 1.52 | 1.53 | 0.99 | 1.85 | 3.29 |
| FxaYL\_631g0012220 | chr6-3-1 28665430 28667478 | 0.71 | 1.03 | 0.92 | 0.48 | 1.75 |
| FxaYL\_632g0012070 | chr6-3-2 28583236 28584702 | 1.99 | 1.29 | 0.28 | 0.31 | 0.40 |

## Slide 27
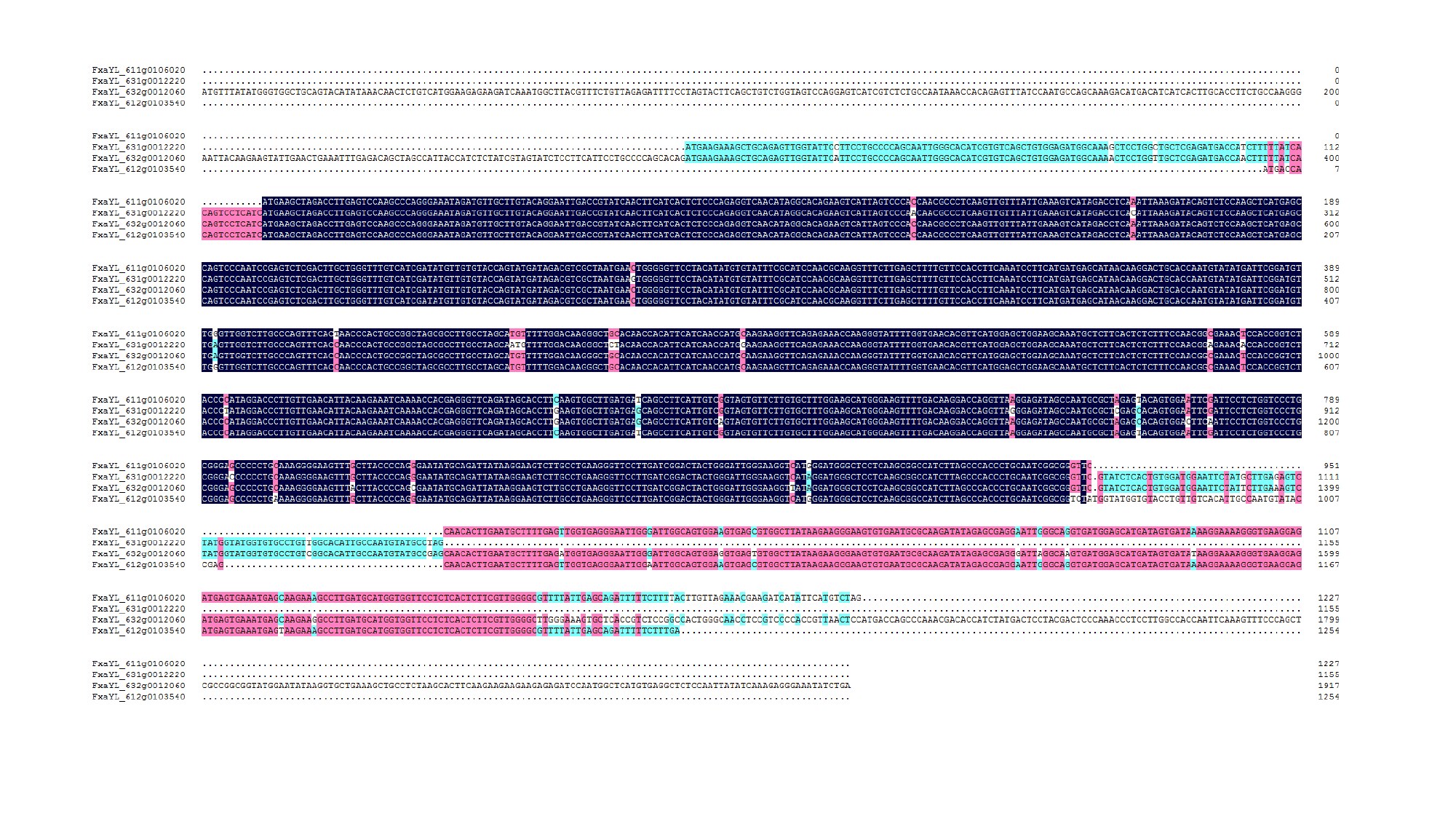

## Slide 28
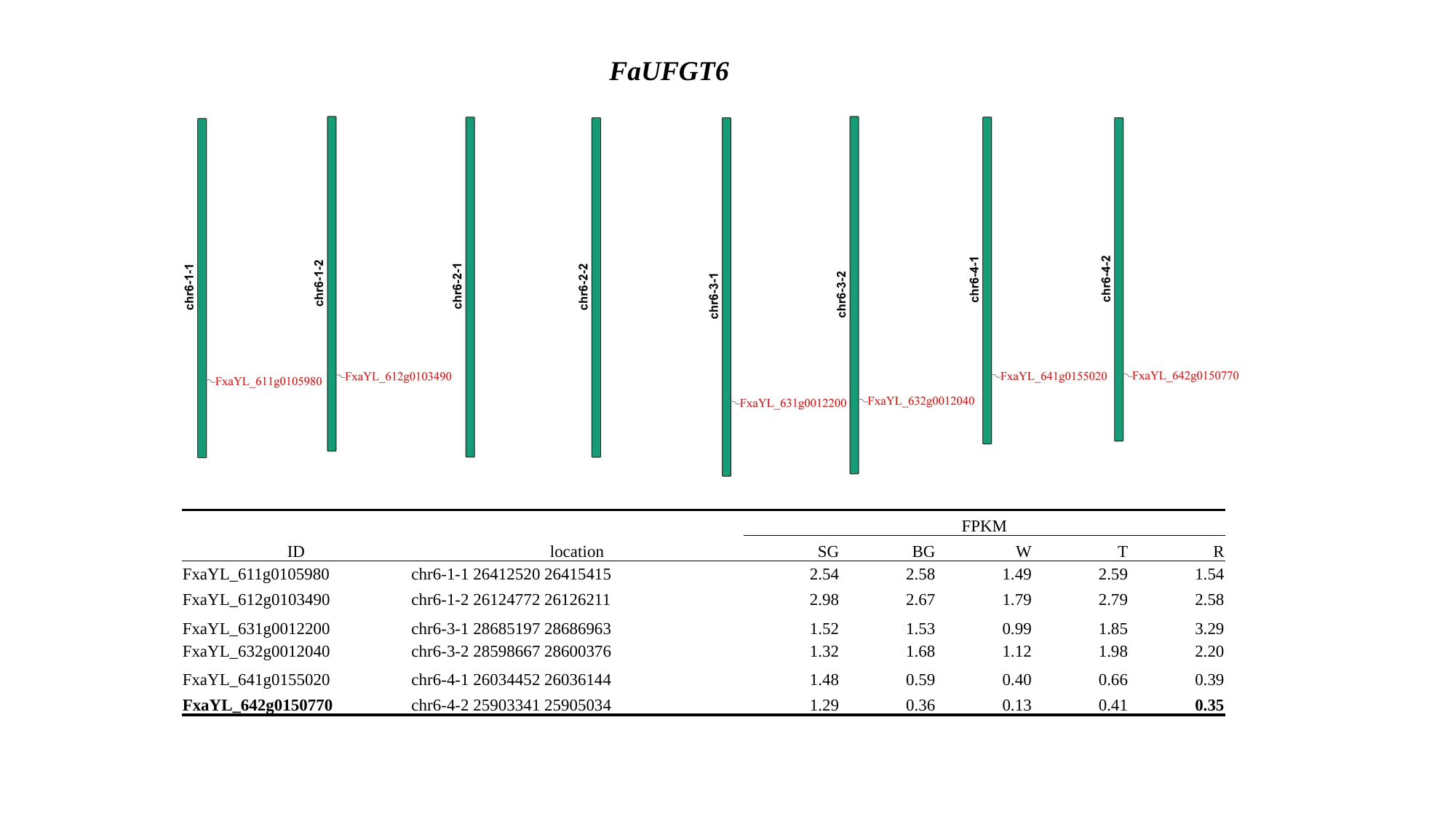

FaUFGT6
| | | FPKM | | | | |
| --- | --- | --- | --- | --- | --- | --- |
| ID | location | SG | BG | W | T | R |
| FxaYL\_611g0105980 | chr6-1-1 26412520 26415415 | 2.54 | 2.58 | 1.49 | 2.59 | 1.54 |
| FxaYL\_612g0103490 | chr6-1-2 26124772 26126211 | 2.98 | 2.67 | 1.79 | 2.79 | 2.58 |
| FxaYL\_631g0012200 | chr6-3-1 28685197 28686963 | 1.52 | 1.53 | 0.99 | 1.85 | 3.29 |
| FxaYL\_632g0012040 | chr6-3-2 28598667 28600376 | 1.32 | 1.68 | 1.12 | 1.98 | 2.20 |
| FxaYL\_641g0155020 | chr6-4-1 26034452 26036144 | 1.48 | 0.59 | 0.40 | 0.66 | 0.39 |
| FxaYL\_642g0150770 | chr6-4-2 25903341 25905034 | 1.29 | 0.36 | 0.13 | 0.41 | 0.35 |

## Slide 29
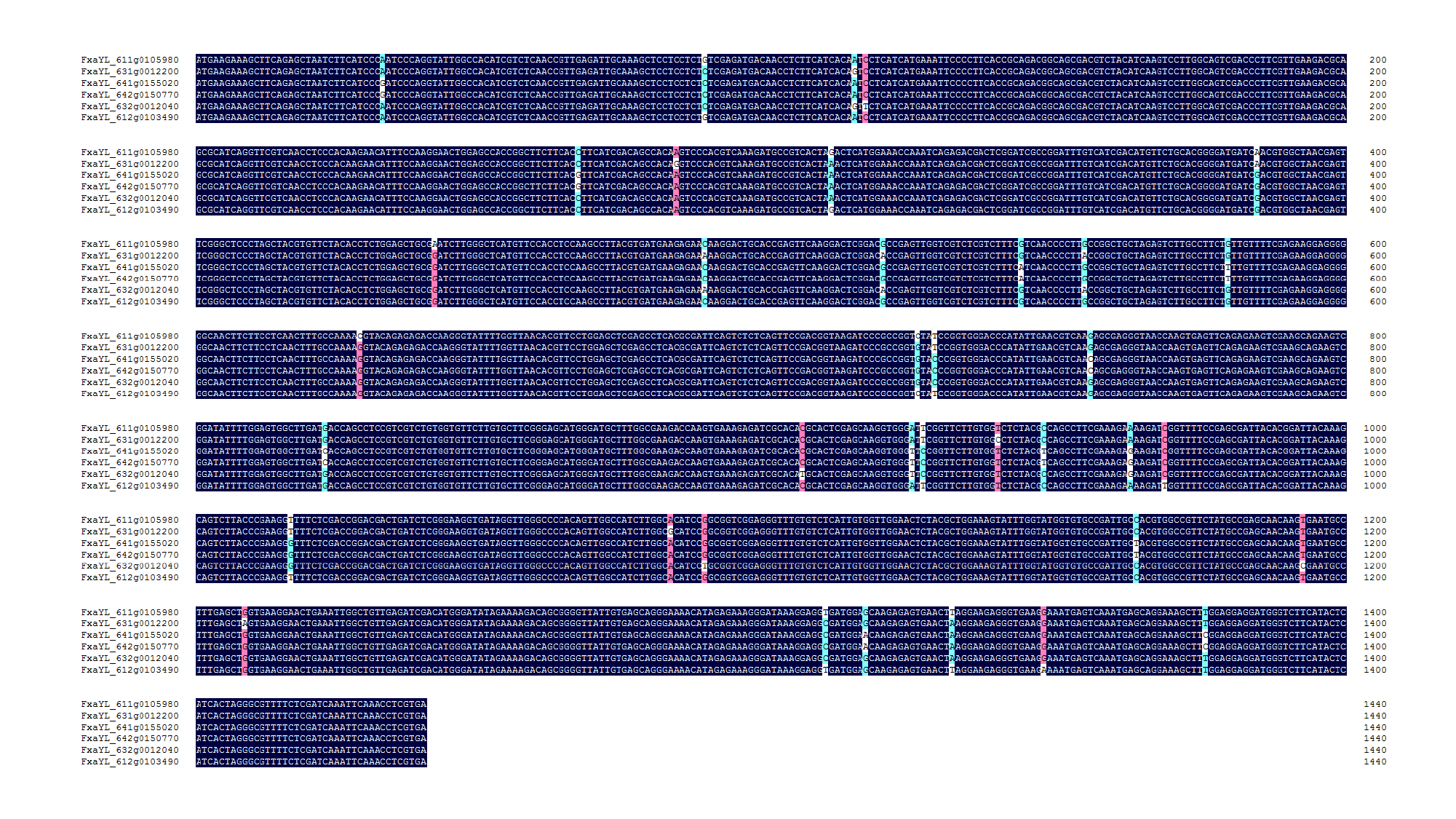

## Slide 30
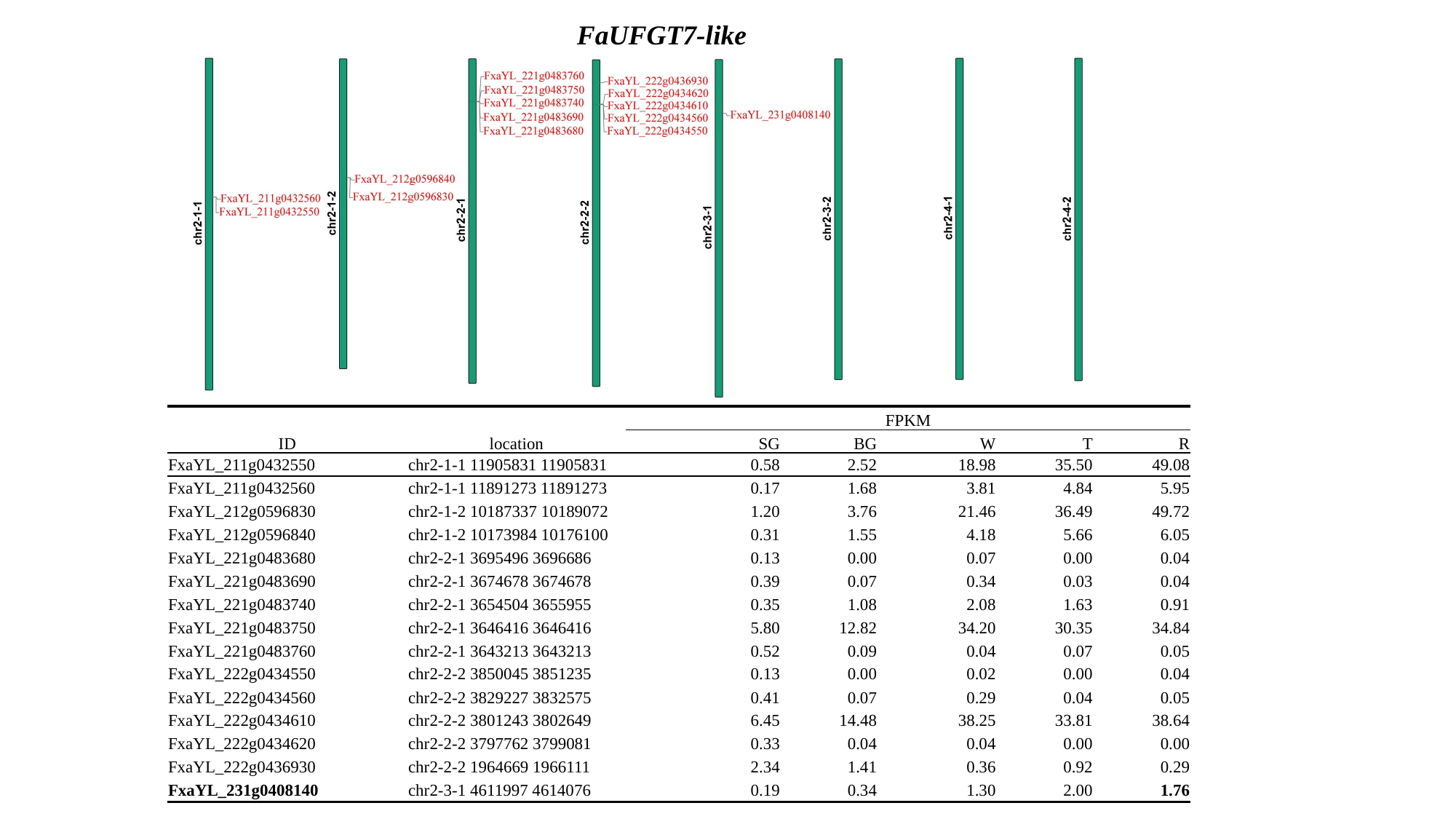

FaUFGT7-like
| | | FPKM | | | | |
| --- | --- | --- | --- | --- | --- | --- |
| ID | location | SG | BG | W | T | R |
| FxaYL\_211g0432550 | chr2-1-1 11905831 11905831 | 0.58 | 2.52 | 18.98 | 35.50 | 49.08 |
| FxaYL\_211g0432560 | chr2-1-1 11891273 11891273 | 0.17 | 1.68 | 3.81 | 4.84 | 5.95 |
| FxaYL\_212g0596830 | chr2-1-2 10187337 10189072 | 1.20 | 3.76 | 21.46 | 36.49 | 49.72 |
| FxaYL\_212g0596840 | chr2-1-2 10173984 10176100 | 0.31 | 1.55 | 4.18 | 5.66 | 6.05 |
| FxaYL\_221g0483680 | chr2-2-1 3695496 3696686 | 0.13 | 0.00 | 0.07 | 0.00 | 0.04 |
| FxaYL\_221g0483690 | chr2-2-1 3674678 3674678 | 0.39 | 0.07 | 0.34 | 0.03 | 0.04 |
| FxaYL\_221g0483740 | chr2-2-1 3654504 3655955 | 0.35 | 1.08 | 2.08 | 1.63 | 0.91 |
| FxaYL\_221g0483750 | chr2-2-1 3646416 3646416 | 5.80 | 12.82 | 34.20 | 30.35 | 34.84 |
| FxaYL\_221g0483760 | chr2-2-1 3643213 3643213 | 0.52 | 0.09 | 0.04 | 0.07 | 0.05 |
| FxaYL\_222g0434550 | chr2-2-2 3850045 3851235 | 0.13 | 0.00 | 0.02 | 0.00 | 0.04 |
| FxaYL\_222g0434560 | chr2-2-2 3829227 3832575 | 0.41 | 0.07 | 0.29 | 0.04 | 0.05 |
| FxaYL\_222g0434610 | chr2-2-2 3801243 3802649 | 6.45 | 14.48 | 38.25 | 33.81 | 38.64 |
| FxaYL\_222g0434620 | chr2-2-2 3797762 3799081 | 0.33 | 0.04 | 0.04 | 0.00 | 0.00 |
| FxaYL\_222g0436930 | chr2-2-2 1964669 1966111 | 2.34 | 1.41 | 0.36 | 0.92 | 0.29 |
| FxaYL\_231g0408140 | chr2-3-1 4611997 4614076 | 0.19 | 0.34 | 1.30 | 2.00 | 1.76 |

## Slide 31
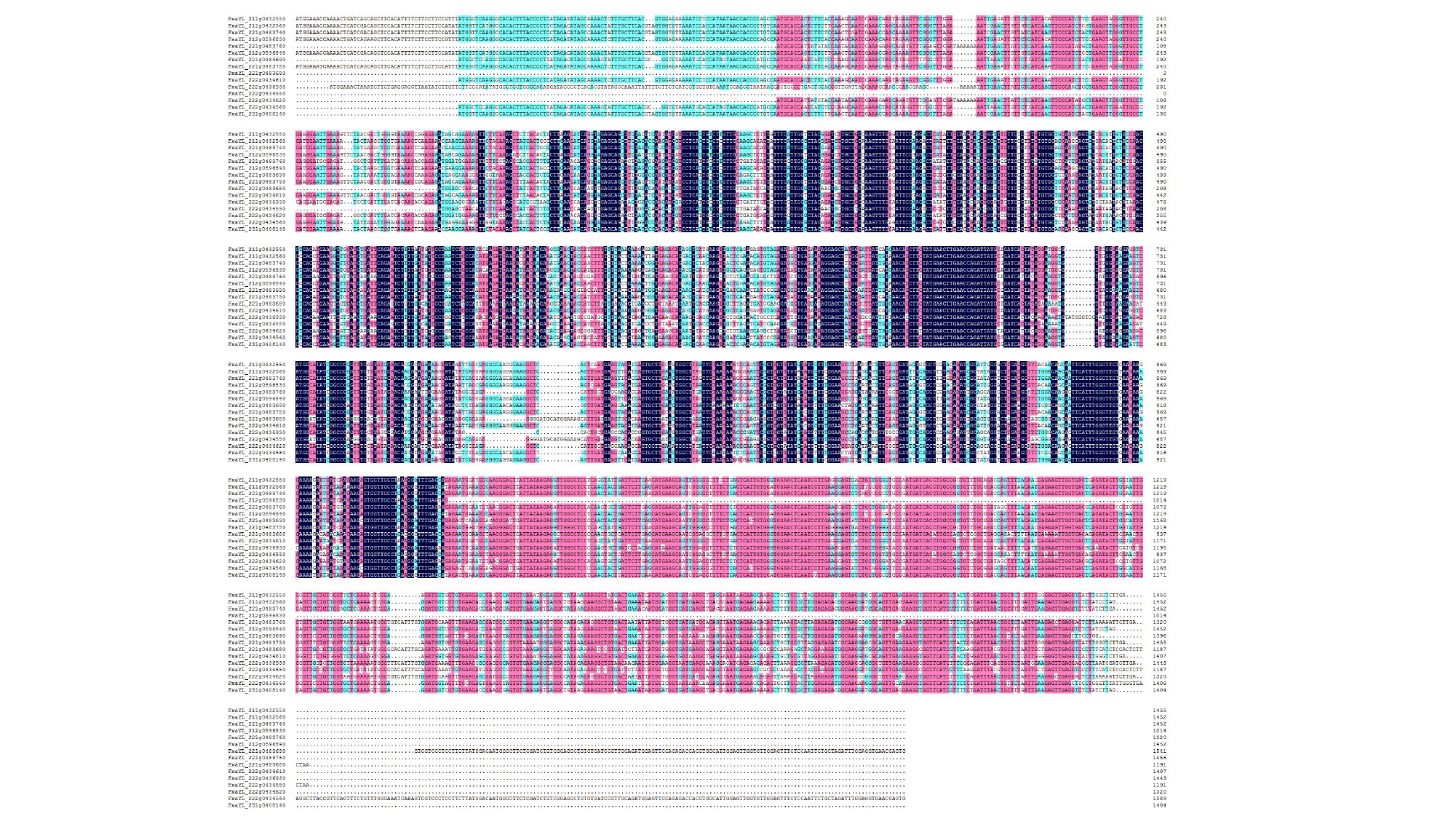

Supplement: Web_Material_uhad002 [file web_material_uhad002.zip › Supplementary File 1.pptx]
