# Supplementary material for: High-quality haplotype-resolved genome assembly of cultivated octoploid strawberry
Source: Hortic Res. 2023 Jan 4;10(1):uhad002. doi: 10.1093/hr/uhad002 (PMC10108017; doi:10.1093/hr/uhad002)
Supplement: Web_Material_uhad002 [file web_material_uhad002.zip › Supplementary Tables.old.docx]

**Table S1 Statistics of DNA sequence datasets**

| Name | Raw Reads (M) | Raw Bases (Gb) | Clean Reads (M) | Clean Bases (Gb) | Clean Q20 (%) | Clean Q30 (%) | Average Length (bp) | GC Content (%) |
| --- | --- | --- | --- | --- | --- | --- | --- | --- |
| SRR8358384 | NA | NA | 32.24 | 50.72 | 97.63 | 95.06 | 160 | 39.64 |
| SRR8358385 | NA | NA | 40.95 | 64.86 | 97.37 | 94.56 | 160 | 39.75 |
| *Fragaria* ***×*** *ananassa* | 82.19 | 123.28 | 80.59 | 117.91 | 97.2 | 92.32 | 150 | 40.26 |

**Table S2 Statistics of sample genome characteristics (K-mer=17)**

| Name | K-mer Number (M) | K-mer Depth | Genome Size (Mb) | Revised Genome Size (Mb) | Heterozygous Ratio (%) | Repeat (%) |
| --- | --- | --- | --- | --- | --- | --- |
| *Fragaria* ***×*** *ananassa* | 5678.51 | 66 | 860 | 850 | 1.04 | 73.98 |

**Table S3. Statistics of sequence assembly**

| Type | Contig Length(bp) | Contig Number | Scaffold Length(bp) | Scaffold Number |
| --- | --- | --- | --- | --- |
| N90 | 22,032,887 | 27 | 22,032,887 | 27 |
| N80 | 23,808,568 | 23 | 23,808,568 | 23 |
| N70 | 26,164,715 | 20 | 26,164,715 | 20 |
| N60 | 26,448,749 | 17 | 26,448,749 | 17 |
| N50 | 26,933,584 | 14 | 26,933,584 | 14 |
| Total length | 823,884,587 |  | 823,884,587 |  |
| Number(>=100bp) |  | 390 |  | 390 |
| Number(>=2kb) |  | 390 |  | 390 |
| Max length | 36,026,591 |  | 36,026,591 |  |

**Table S4. Statistics of mapping and coverage rates**

| Type | Second generation data | Third generation data |
| --- | --- | --- |
| Mapping rate(%) | 99.61 | 99.98 |
| Average sequencing depth | 95.29 | 44.10 |
| Coverage(%) | 99.94 | 99.86 |
| Coverage at least 4X(%) | 99.88 | 99.17 |
| Coverage at least 10X(%) | 99.76 | 98.47 |
| Coverage at least 20X(%) | 99.41 | 92.69 |

**Table S5. BUSCO assessment of *De novo* assembly**

| Type | Proteins | Percentage(%) |
| --- | --- | --- |
| Complete BUSCOs (C) | 1,343 | 93.26 |
| Complete and single-copy BUSCOs (S) | 99 | 6.88 |
| Complete and duplicated BUSCOs (D) | 1,244 | 86.39 |
| Fragmented BUSCOs (F) | 18 | 1.25 |
| Missing BUSCOs (M) | 79 | 5.49 |
| Total BUSCO groups searched | 1,440 | 100.00 |

**Table S6. Statistics of LAI index**

| Name | Start site | End site | Intact LTR rate(%) | Total LTR rate(%) | Raw LAI | LAI |
| --- | --- | --- | --- | --- | --- | --- |
| *Fragaria* ***×*** *ananassa* | 1 | 823,884,587 | 3.94 | 26.97 | 14.61 | 14.99 |

**Table S7. Statistics of homozygosity and heterozygosity**

| Name | Homozygous SNP rate(%) | Homozygous InDel  rate(%) | Heterozygous SNP rate(%) | Heterozygous InDel rate(%) |
| --- | --- | --- | --- | --- |
| *Fragaria* ***×*** *ananassa* | 0.000 | 0.000 | 0.612 | 0.094 |

**Table S8. Statistics of Hi-C assembly**

| Type | Haplotype 1 | Haplotype 2 |
| --- | --- | --- |
| Total Contigs Length (Mb) | 824.84 | 808.07 |
| Contigs Number | 628 | 278 |
| Contig N50 (Mb) | 26.70 | 27.50 |
| Total scaffolds Length (Mb) | 824.84 | 808.07 |
| Scaffolds Number | 647 | 316 |
| Scaffold N50 (Mb) | 27.31 | 27.50 |
| Anchored Length (Mb) | 783.67 | 778.08 |
| Chromosomes Number | 28 | 28 |
| Unanchored Length (Mb) | 41.17 | 29.99 |
| Unanchored Number | 619 | 288 |

**Table S9. BUSCO assessment of Hi-C assembly**

| Type | Haplotype 1 | | Haplotype 2 | |
| --- | --- | --- | --- | --- |
|  | Proteins | Percentage (%) | Proteins | Percentage (%) |
| Complete BUSCOs | 1,585 | 98.2 | 1,583 | 98.0 |
| Complete Single-Copy BUSCOs | 87 | 5.4 | 78 | 4.8 |
| Complete Duplicated BUSCOs | 1,498 | 92.8 | 1,505 | 93.2 |
| Fragmented BUSCOs | 4 | 0.2 | 3 | 0.2 |
| Missing BUSCOs | 25 | 1.6 | 28 | 1.8 |
| Total BUSCO | 1,614 | 100.0 | 1,614 | 100.0 |

**Table S10. Statistics of chromosomes contained in Hap1 and Hap2**

| Haplotypes | Chromosomes | Haplotypes | Chromosomes |
| --- | --- | --- | --- |
| Hap1 | chr1-1-1 | Hap2 | chr1-1-2 |
|  | chr1-2-1 |  | chr1-2-2 |
|  | chr1-3-1 |  | chr1-3-2 |
|  | chr1-4-1 |  | chr1-4-2 |
|  | chr2-1-1 |  | chr2-1-2 |
|  | chr2-2-1 |  | chr2-2-2 |
|  | chr2-3-1 |  | chr2-3-2 |
|  | chr2-4-1 |  | chr2-4-2 |
|  | chr3-1-1 |  | chr3-1-2 |
|  | chr3-2-1 |  | chr3-2-2 |
|  | chr3-3-1 |  | chr3-3-2 |
|  | chr3-4-1 |  | chr3-4-2 |
|  | chr4-1-1 |  | chr4-1-2 |
|  | chr4-2-1 |  | chr4-2-2 |
|  | chr4-3-1 |  | chr4-3-2 |
|  | chr4-4-1 |  | chr4-4-2 |
|  | chr5-1-1 |  | chr5-1-2 |
|  | chr5-2-1 |  | chr5-2-2 |
|  | chr5-3-1 |  | chr5-3-2 |
|  | chr5-4-1 |  | chr5-4-2 |
|  | chr6-1-1 |  | chr6-1-2 |
|  | chr6-2-1 |  | chr6-2-2 |
|  | chr6-3-1 |  | chr6-3-2 |
|  | chr6-4-1 |  | chr6-4-2 |
|  | chr7-1-1 |  | chr7-1-2 |
|  | chr7-2-1 |  | chr7-2-2 |
|  | chr7-3-1 |  | chr7-3-2 |
|  | chr7-4-1 |  | chr7-4-2 |

**Table S11. Statistics of repeat sequences**

| Type | Haplotype 1 | | Haplotype 2 | |
| --- | --- | --- | --- | --- |
|  | Repeat Size (bp) | Percentage of genome (%) | Repeat Size (bp) | Percentage of genome (%) |
| Trf | 54,887,176 | 6.65 | 52,900,393 | 6.55 |
| Repeatmasker | 205,523,479 | 24.92 | 201,996,281 | 25.00 |
| Proteinmask | 58,066,013 | 7.04 | 57,448,533 | 7.11 |
| *De novo* | 308,113,818 | 37.35 | 294,196,602 | 36.41 |
| Total | 371,599,699 | 45.05 | 353,542,770 | 43.75 |

**Table S12. Statistics of repetitive sequence classification**

| Type | Haplotype 1 | | Haplotype 2 | |
| --- | --- | --- | --- | --- |
|  | Length (bp) | Percentage of genome (%) | Length (bp) | Percentage of genome (%) |
| DNA | 109,648,634 | 13.29 | 98,103,835 | 12.14 |
| LINE | 13,618,172 | 1.65 | 12,798,543 | 1.58 |
| SINE | 1,488,932 | 0.18 | 447,347 | 0.06 |
| LTR | 199,559,880 | 24.19 | 201,215,189 | 24.90 |
| Satellite | 2,831,172 | 0.34 | 2,711,483 | 0.34 |
| Simple_repeat | 1,399 | 0.00 | 251 | 0.00 |
| Other | 985 | 0.00 | 632 | 0.00 |
| Unknown | 42,718,330 | 5.18 | 32,628,940 | 4.04 |
| Total | 355,601,928 | 43.11 | 337,462,998 | 41.76 |

**Table S13. Statistics of gene metrics**

| Name | Haplotype 1 | | | Haplotype 2 | | |
| --- | --- | --- | --- | --- | --- | --- |
| Gene set | Protein coding gene number | Average gene length (bp) | Average CDS length (bp) | Protein coding gene number | Average gene length (bp) | Average CDS length (bp) |
| GlimmmerHMM | 124,383 | 5,663 | 1,964 | 122,413 | 5,681 | 1,941 |
| AUGUSTUS | 88,667 | 2,876 | 1,291 | 79,795 | 3,182 | 1,386 |
| *Prunus_avium* | 113,191 | 4,449 | 1,119 | 113,011 | 4,519 | 1,118 |
| *Fragaria_vesca* | 124,338 | 4,012 | 1,163 | 124,463 | 4,056 | 1,163 |
| *Malus* ***×*** *domestica* | 115,173 | 4,353 | 1,112 | 115,086 | 4,502 | 1,116 |
| *Rosa_chinensis* | 134,862 | 4,610 | 1,157 | 135,095 | 4,805 | 1,157 |
| *Fragaria* ***×*** *ananassa*_ng | 316,035 | 3,817 | 758 | 313,627 | 3,852 | 764 |
| RNA-seq | 47,991 | 4,704 | 1,523 | 48,215 | 4,754 | 1,525 |
| ISO-seq | 43,583 | 7,157 | 1,575 | 44,778 | 7,305 | 1,575 |
| BUSCO | 4,297 | 4,398 | 1,726 | 4,321 | 4,390 | 1,730 |
| MAKER | 82,997 | 5,032 | 1,250 | 81,114 | 5,167 | 1,269 |
| HiCESAP | 106,049 | 4,089 | 1,377 | 103,213 | 4,243 | 1,395 |

**Table S14. Statistics of gene functional annotation**

| Type | Haplotype 1 | | Haplotype 2 | |
| --- | --- | --- | --- | --- |
|  | Number | Percent (%) | Number | Percent (%) |
| Total | 104,957 |  | 102,356 |  |
| Annotated | 93,210 | 88.81 | 91,802 | 89.69 |
| InterPro | 72,056 | 68.65 | 71,099 | 69.46 |
| GO | 49,940 | 47.58 | 49,371 | 48.23 |
| KEGG_ALL | 91,053 | 86.75 | 89,706 | 87.64 |
| KEGG_KO | 33,288 | 31.72 | 32,810 | 32.05 |
| Swissprot | 58,506 | 55.74 | 57,604 | 56.28 |
| TrEMBL | 90,161 | 85.90 | 88,845 | 86.80 |
| TF | 5,831 | 5.56 | 5,804 | 5.67 |
| Pfam | 70,644 | 67.31 | 69,705 | 68.10 |
| NR | 92,220 | 87.86 | 90,851 | 88.76 |
| KOG | 70,450 | 67.12 | 70,054 | 68.44 |
| Unannotated | 11,747 | 11.19 | 10,554 | 10.31 |

**Table S15. BUSCO assessment of annotation**

| Type | Haplotype 1 | | Haplotype 2 | |
| --- | --- | --- | --- | --- |
|  | Proteins | Percentage (%) | Proteins | Percentage (%) |
| Complete BUSCOs | 1,607 | 99.6 | 1,607 | 99.6 |
| Complete Single-Copy BUSCOs | 45 | 2.8 | 40 | 2.5 |
| Complete Duplicated BUSCOs | 1,562 | 96.8 | 1,567 | 97.1 |
| Fragmented BUSCOs | 0 | 0.0 | 1 | 0.1 |
| Missing BUSCOs | 7 | 0.4 | 6 | 0.3 |
| Total BUSCO groups searched | 1,614 | 100.0 | 1,614 | 100.0 |

**Table S16. Statistics of noncoding RNAs (ncRNAs)**

| Type | Haplotype 1 | | | | Haplotype 2 | | | |
| --- | --- | --- | --- | --- | --- | --- | --- | --- |
|  | Copy | Average length (bp) | Total length (bp) | Percentage of genome (%) | Copy | Average length (bp) | Total length (bp) | Percentage of genome (%) |
| mRNA | 2,546 | 127 | 48,134 | 0.005836 | 2,508 | 126 | 49,350 | 0.006107 |
| tRNA | 5,742 | 75 | 433,259 | 0.052526 | 3,707 | 75 | 279,499 | 0.034588 |
| rRNA | 6,451 | 453 | 2,920,663 | 0.354088 | 7,019 | 489 | 3,435,740 | 0.425176 |
| snRNA | 1,353 | 113 | 153,518 | 0.018612 | 1,331 | 114 | 151,939 | 0.018803 |

**Table S17. Statistics of RNA-sequence**

| Sample  name | Total raw  reads (M) | Total clean  reads (M) | Total clean  Base (G) | Clean  reads  Q20(%) | Clean  reads  Q30(%) | Clean  reads  ratio(%) |
| --- | --- | --- | --- | --- | --- | --- |
| SG fruit-1 | 39.19 | 38.93 | 5.79 | 97.85 | 93.74 | 99.34 |
| SG fruit-2 | 46.09 | 45.81 | 6.83 | 97.98 | 94.07 | 99.37 |
| SG fruit-3 | 39.87 | 39.63 | 5.90 | 97.84 | 93.73 | 99.40 |
| BG fruit-1 | 39.19 | 38.93 | 5.79 | 97.85 | 93.74 | 99.34 |
| BG fruit-2 | 46.09 | 45.81 | 6.83 | 97.98 | 94.07 | 99.37 |
| BG fruit-3 | 39.87 | 39.63 | 5.90 | 97.84 | 93.73 | 99.40 |
| W fruit-1 | 53.54 | 53.21 | 7.90 | 97.90 | 93.92 | 99.39 |
| W fruit-2 | 39.79 | 39.59 | 5.87 | 97.97 | 93.99 | 99.51 |
| W fruit-3 | 50.09 | 49.85 | 7.39 | 98.05 | 94.17 | 99.53 |
| T fruit-1 | 39.53 | 39.26 | 5.83 | 97.61 | 93.21 | 99.32 |
| T fuirt-2 | 42.22 | 41.94 | 6.24 | 97.61 | 93.17 | 99.32 |
| T fruit-3 | 42.36 | 42.15 | 6.27 | 97.91 | 93.87 | 99.51 |
| R fruit-1 | 41.06 | 40.85 | 6.05 | 97.81 | 93.65 | 99.49 |
| R fruit-2 | 46.72 | 46.43 | 6.89 | 97.68 | 93.35 | 99.38 |
| R fruit-3 | 40.09 | 39.88 | 5.92 | 97.81 | 93.65 | 99.47 |
| Root | 39.30 | 38.80 | 5.81 | 97.94 | 93.75 | 98.71 |
| Leave | 38.72 | 38.35 | 5.74 | 98.03 | 93.96 | 99.04 |
| Shoot tip | 124.89 | 119.49 | 17.89 | 97.32 | 93.42 | 95.68 |

SG=small green; BG=big green; W=white; T=turning; R=ripening.

The samples of R fruit-3, Root, Leave and Shoot tip were used for gene expression profile analysis in different tissues/organs.

**Table S18. Expression of *FaMYB10* gene throughout fruit development**

|  |  | FPKM | | | | |
| --- | --- | --- | --- | --- | --- | --- |
| ID | location | SG | BG | W | T | R |
| FxaYL_111g0826310 | chr1-1-1 12909065 12920603 | 0.00 | 0.06 | 0.01 | 0.08 | 0.20 |
| FxaYL_112g0700260 | chr1-1-2 12829775 12840893 | 0.00 | 0.06 | 0.00 | 0.06 | 0.33 |
| FxaYL_121g0707540 | chr1-2-1 13188780 13204621 | 0.21 | 0.40 | 11.29 | 75.79 | 92.89 |
| FxaYL_122g0790550 | chr1-2-2 12894713 12899940 | 0.97 | 1.28 | 34.65 | 232.32 | 284.92 |
| FxaYL_131g0769020 | chr1-3-1 13701963 13703662 | 0.04 | 0.00 | 0.30 | 0.94 | 1.09 |
| FxaYL_132g0761430 | chr1-3-2 13884105 13892409 | 0.04 | 0.00 | 0.22 | 0.80 | 0.36 |
| FxaYL_141g0885740 | chr1-4-1 12945884 12947911 | 0.00 | 0.00 | 0.05 | 0.34 | 3.31 |
| FxaYL_142g0880670 | chr1-4-2 12801199 12804404 | 0.00 | 0.00 | 0.07 | 0.33 | 3.79 |

**Table S19. Expression of *FaANS*** **gene throughout fruit development**

|  |  | FPKM | | | | |
| --- | --- | --- | --- | --- | --- | --- |
| ID | location | SG | BG | W | T | R |
| FxaYL_511g0695310 | chr5-1-1 745338 747363 | 53.77 | 68.14 | 251.60 | 904.84 | 823.23 |
| FxaYL_512g0645780 | chr5-1-2 780565 782696 | 50.68 | 63.93 | 238.96 | 884.19 | 803.59 |
| FxaYL_521g0758970 | chr5-2-1 688457 690080 | 2.09 | 0.22 | 0.85 | 10.35 | 7.61 |
| FxaYL_522g0613100 | chr5-2-2 810100 811396 | 1.93 | 0.26 | 3.66 | 40.77 | 33.75 |
| FxaYL_531g0590000 | chr5-3-1 778573 780363 | 4.53 | 1.18 | 50.33 | 584.72 | 519.55 |
| FxaYL_532g0366230 | chr5-3-2 957383 959165 | 4.30 | 1.07 | 44.59 | 522.55 | 463.72 |
| FxaYL_541g0621860 | chr5-4-1 623303 624533 | 0.04 | 0.07 | 0.78 | 4.45 | 4.13 |

**Table S20. Expression of *FaCHI3* gene throughout fruit development**

|  |  | FPKM | | | | |
| --- | --- | --- | --- | --- | --- | --- |
| ID | location | SG | BG | W | T | R |
| FxaYL_711g0943130 | chr7-1-1 18941027 18942672 | 0.37 | 0.32 | 4.19 | 60.89 | 35.38 |
| FxaYL_712g0929570 | chr7-1-2 18634450 18636122 | 0.37 | 0.30 | 3.87 | 57.77 | 33.46 |
| FxaYL_721g0973800 | chr7-2-1 18187394 18189957 | 16.04 | 16.72 | 110.02 | 402.67 | 333.43 |
| FxaYL_722g0898650 | chr7-2-2 18040819 18043027 | 19.46 | 20.50 | 137.73 | 513.54 | 423.91 |
| FxaYL_731g0852700 | chr7-3-1 19512658 19514219 | 23.00 | 30.28 | 174.63 | 662.74 | 467.54 |
| FxaYL_732g0836520 | chr7-3-2 19155419 19156980 | 23.03 | 29.81 | 170.95 | 645.69 | 454.76 |
| FxaYL_741g0914810 | chr7-4-1 18904896 18906451 | 8.51 | 13.50 | 129.25 | 432.12 | 319.10 |
| FxaYL_742g0957920 | chr7-4-2 17814584 17816139 | 9.32 | 14.34 | 130.77 | 431.20 | 318.28 |

**Table S21. Expression of *FaCHI1*** **gene throughout fruit development**

|  |  | FPKM | | | | |
| --- | --- | --- | --- | --- | --- | --- |
| ID | location | SG | BG | W | T | R |
| FxaYL_711g0946610 | chr7-1-1 16823351 16825321 | 9.01 | 4.76 | 41.88 | 223.08 | 163.48 |
| FxaYL_712g0932910 | chr7-1-2 16622997 16624954 | 9.27 | 4.92 | 43.09 | 228.42 | 167.67 |
| FxaYL_721g0978030 | chr7-2-1 15712798 15714577 | 63.47 | 36.53 | 87.01 | 394.21 | 305.43 |
| FxaYL_722g0902760 | chr7-2-2 15559002 15560782 | 63.27 | 36.50 | 76.94 | 319.98 | 249.66 |
| FxaYL_731g0856620 | chr7-3-1 17034511 17035404 | 0.00 | 0.00 | 0.00 | 0.00 | 0.00 |
| FxaYL_731g0856630 | chr7-3-1 17031285 17033476 | 1.52 | 0.64 | 1.44 | 5.37 | 4.19 |
| FxaYL_732g0840260 | chr7-3-2 16679456 16681647 | 1.50 | 0.59 | 1.30 | 5.15 | 3.84 |
| FxaYL_741g0918870 | chr7-4-1 16503908 16505793 | 7.32 | 3.19 | 23.33 | 140.29 | 97.40 |
| FxaYL_742g0961860 | chr7-4-2 15430520 15433959 | 2.26 | 0.90 | 11.04 | 72.07 | 51.07 |
